# Supplementary figures and images for: Ambient air pollution and cause-specific risk of hospital admission in China: A nationwide time-series study
Source: PLoS Med. 2020 Aug 6;17(8):e1003188. doi: 10.1371/journal.pmed.1003188 (PMC7410211; doi:10.1371/journal.pmed.1003188)

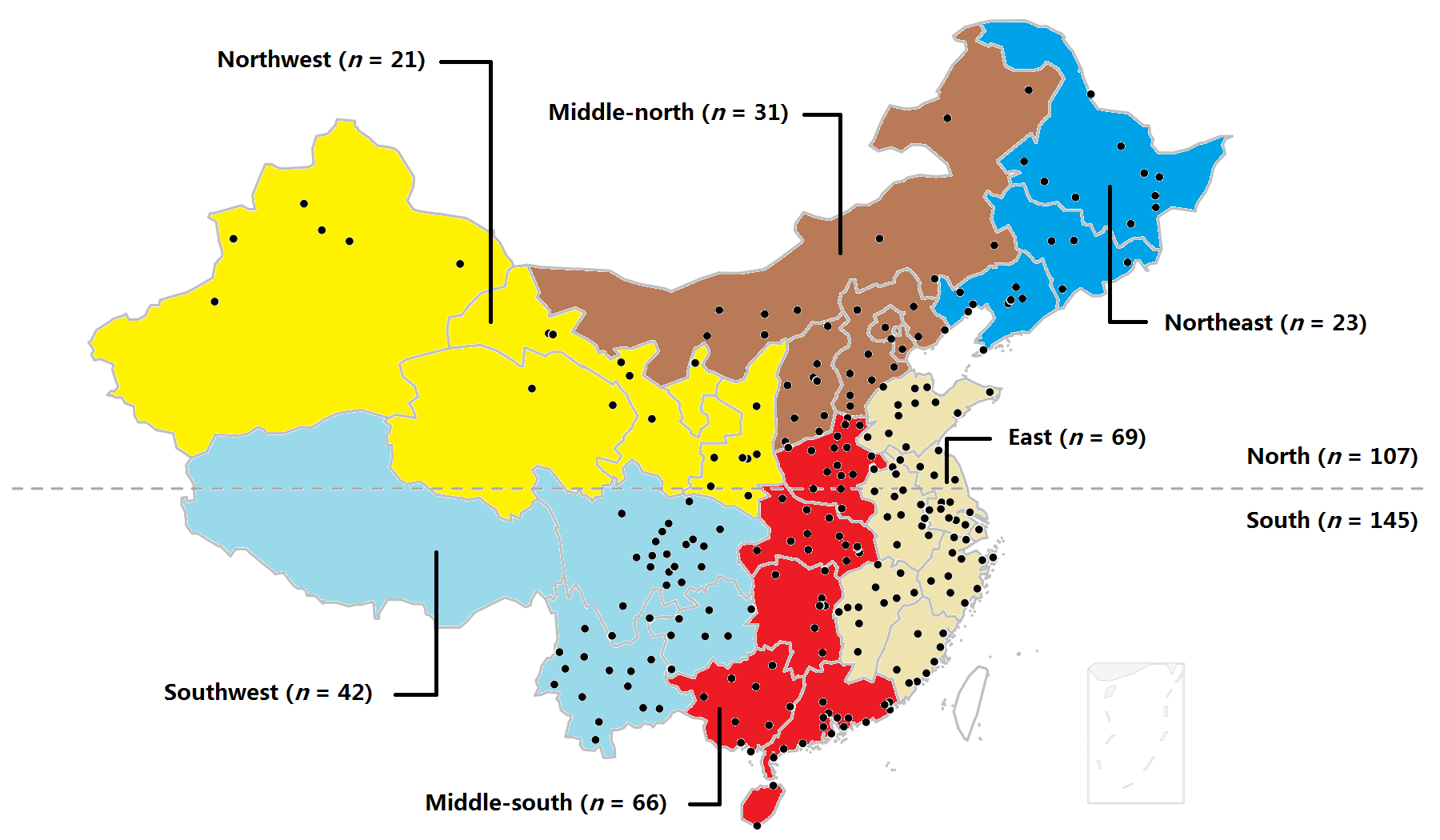

Supplement: S1 Fig — The base map was created using the open-source R package maps. (TIF) [file pmed.1003188.s001.tif]
