# Supplementary material for: Ambient air pollution and cause-specific risk of hospital admission in China: A nationwide time-series study
Source: PLoS Med. 2020 Aug 6;17(8):e1003188. doi: 10.1371/journal.pmed.1003188 (PMC7410211; doi:10.1371/journal.pmed.1003188)
Supplement: S2 Fig — (DOCX) [file pmed.1003188.s002.docx]

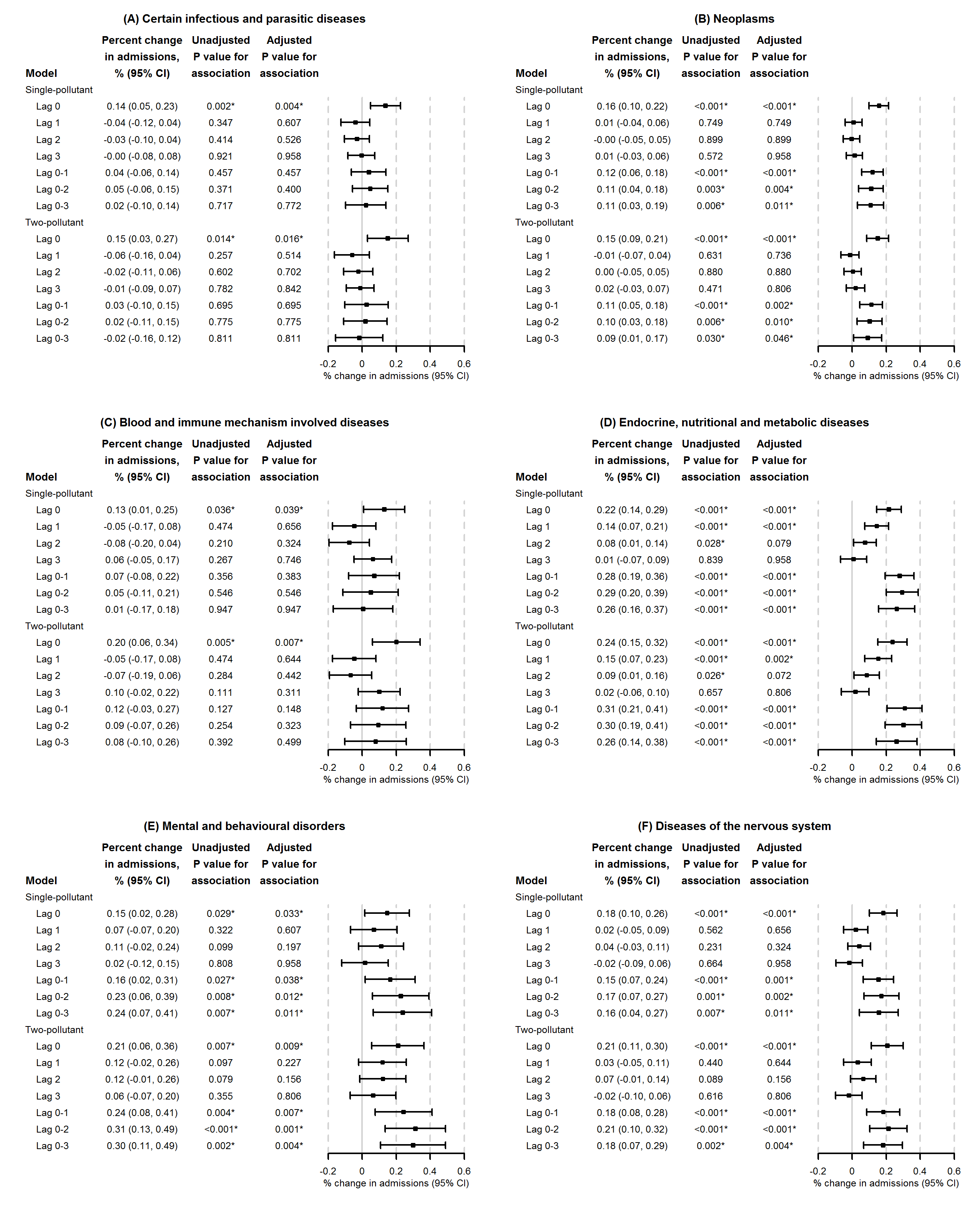


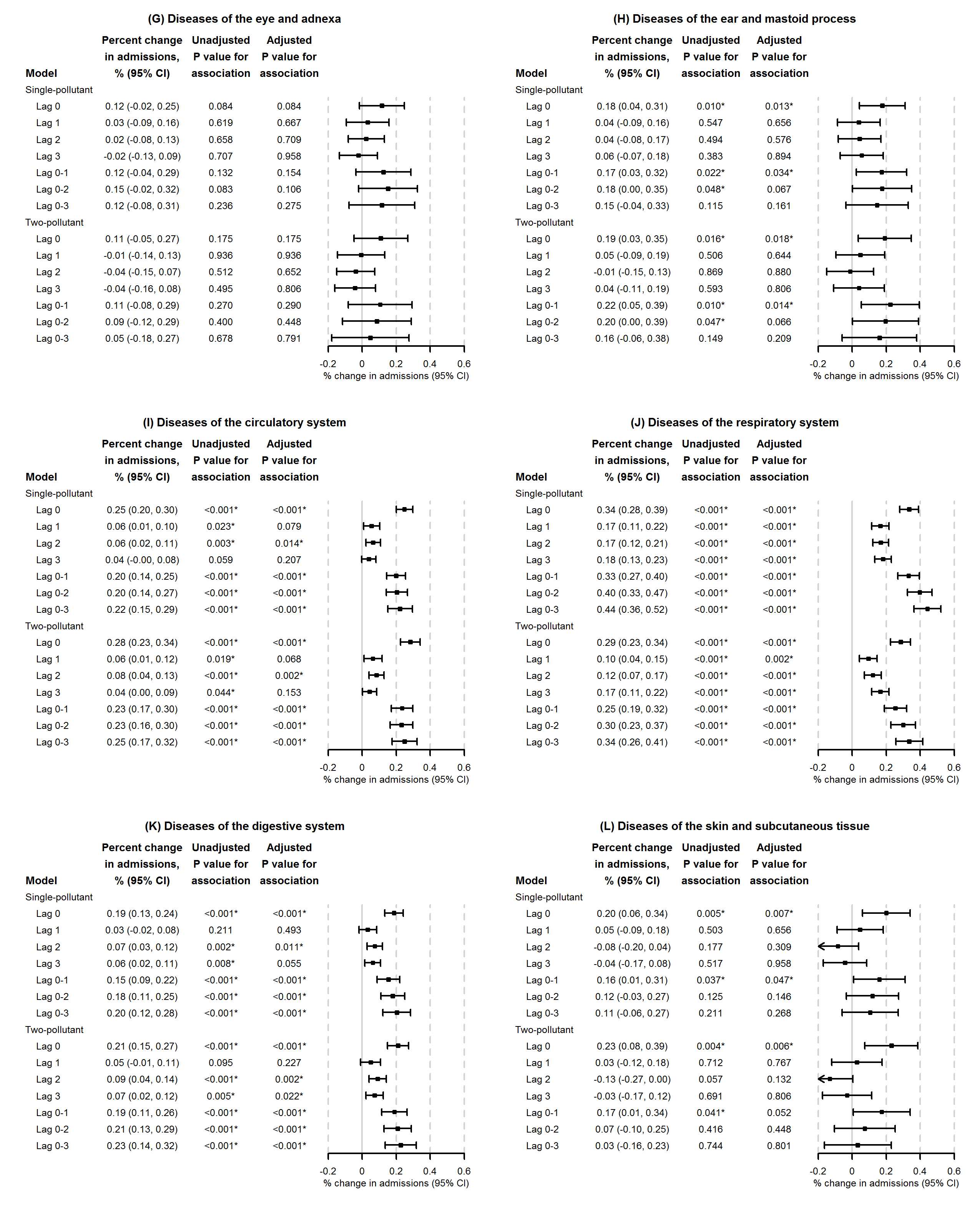


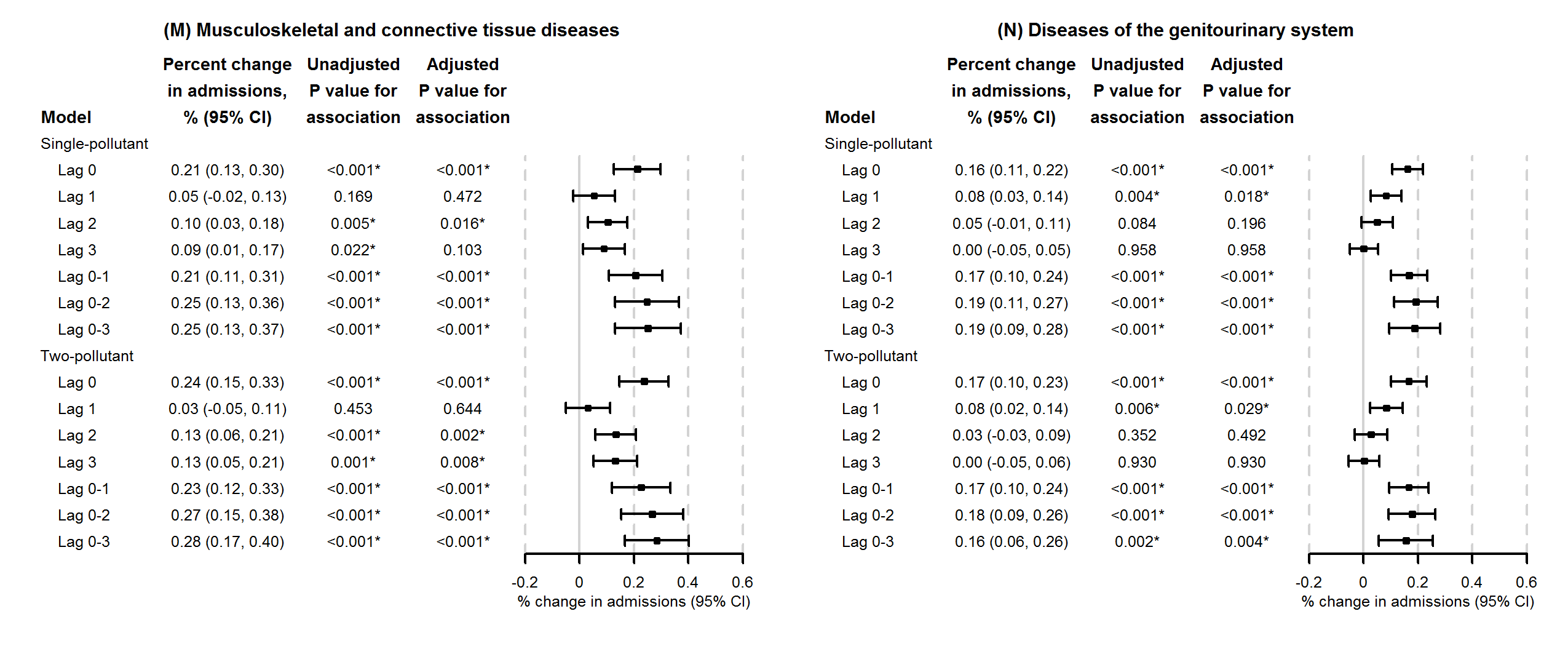


# S2 Fig. Percent change in hospital admissions per 10-μg/m^3^ increase in PM_2.5_ by major disease categories using different lag structures, on average across all cities.

Results are presented as point estimates and 95% CIs of the percentage increase in daily hospital admissions associated with a 10-μg/m^3^ increase in PM_2.5_. Major disease categories are based on the chapter division of the ICD-10 diagnostic coding system. “Lag 0”, “Lag 1”, “Lag 2”, and “Lag 3” indicate that the single-day exposures on the same day, the previous day, the previous 2 days, and the previous 3 days were used as the exposure metric of PM_2.5_, respectively. “Lag 0-1”, “Lag 0-2”, and “Lag 0-3” indicate that the 2-day, the 3-day, and the 4-day moving average exposures were used as the exposure metric of PM_2.5_, respectively. In single-pollutant models, the effects of PM_2.5_ were estimated without adjustment for co-pollutants; in two-pollutant models, the effects of PM_2.5_ were estimated after adjustment for O_3_. The Benjamini-Hochberg procedure was applied to adjust the *P* values across 14 major disease categories; both unadjusted and adjusted *P* values are reported.

* Statistically significant estimate (*P* < 0.05).
