# Supplementary material for: Ambient air pollution and cause-specific risk of hospital admission in China: A nationwide time-series study
Source: PLoS Med. 2020 Aug 6;17(8):e1003188. doi: 10.1371/journal.pmed.1003188 (PMC7410211; doi:10.1371/journal.pmed.1003188)
Supplement: S5 Fig — (DOCX) [file pmed.1003188.s005.docx]

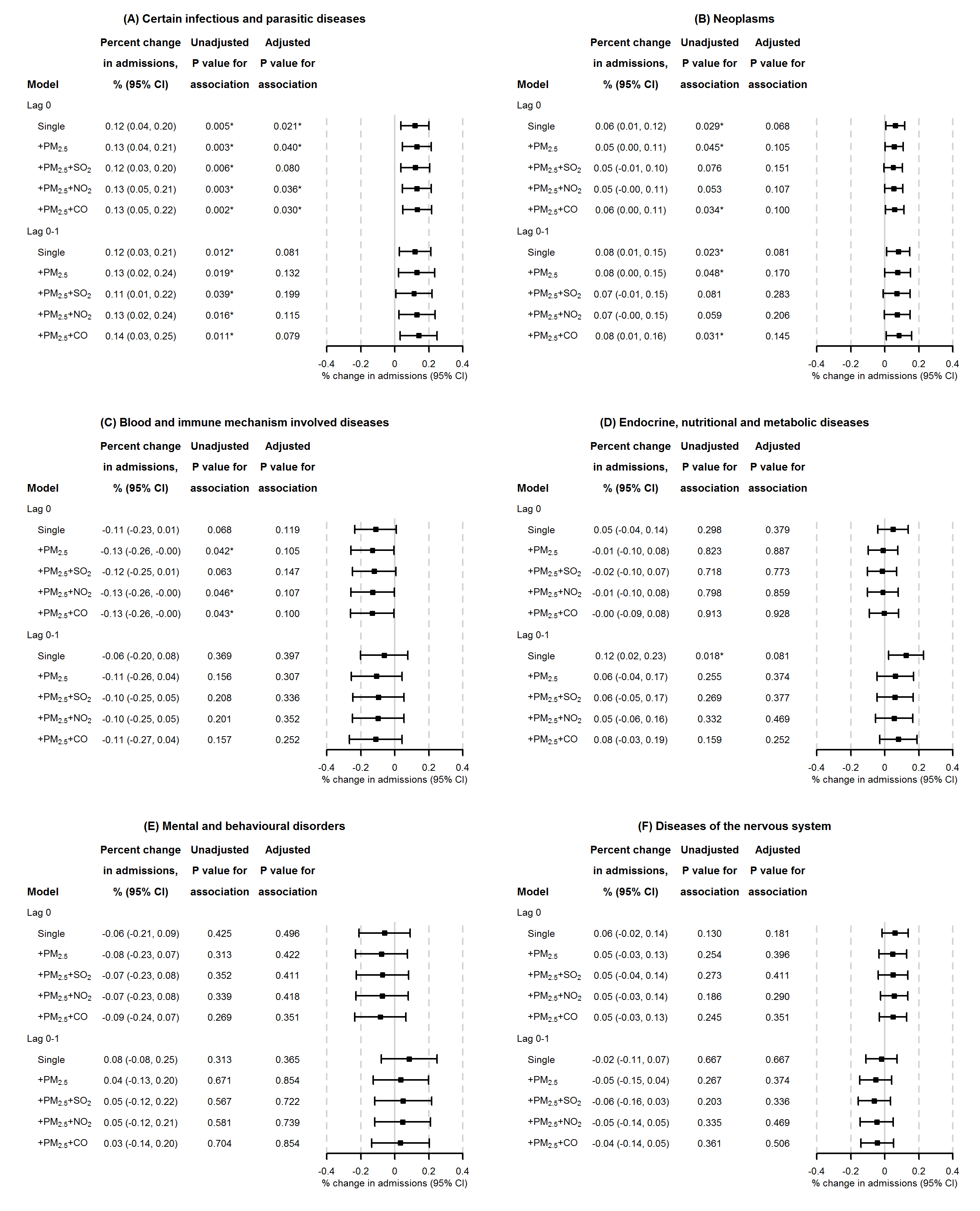


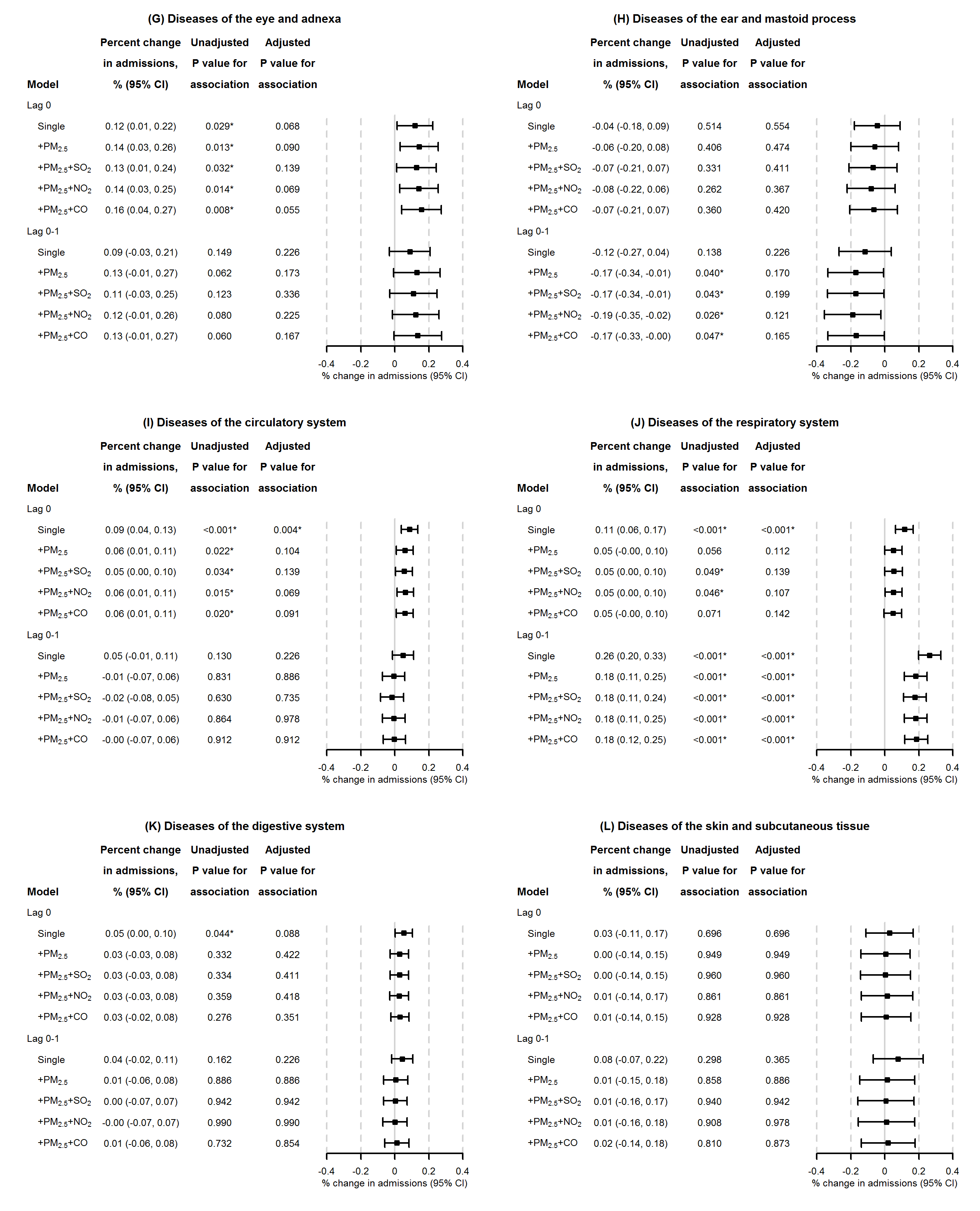


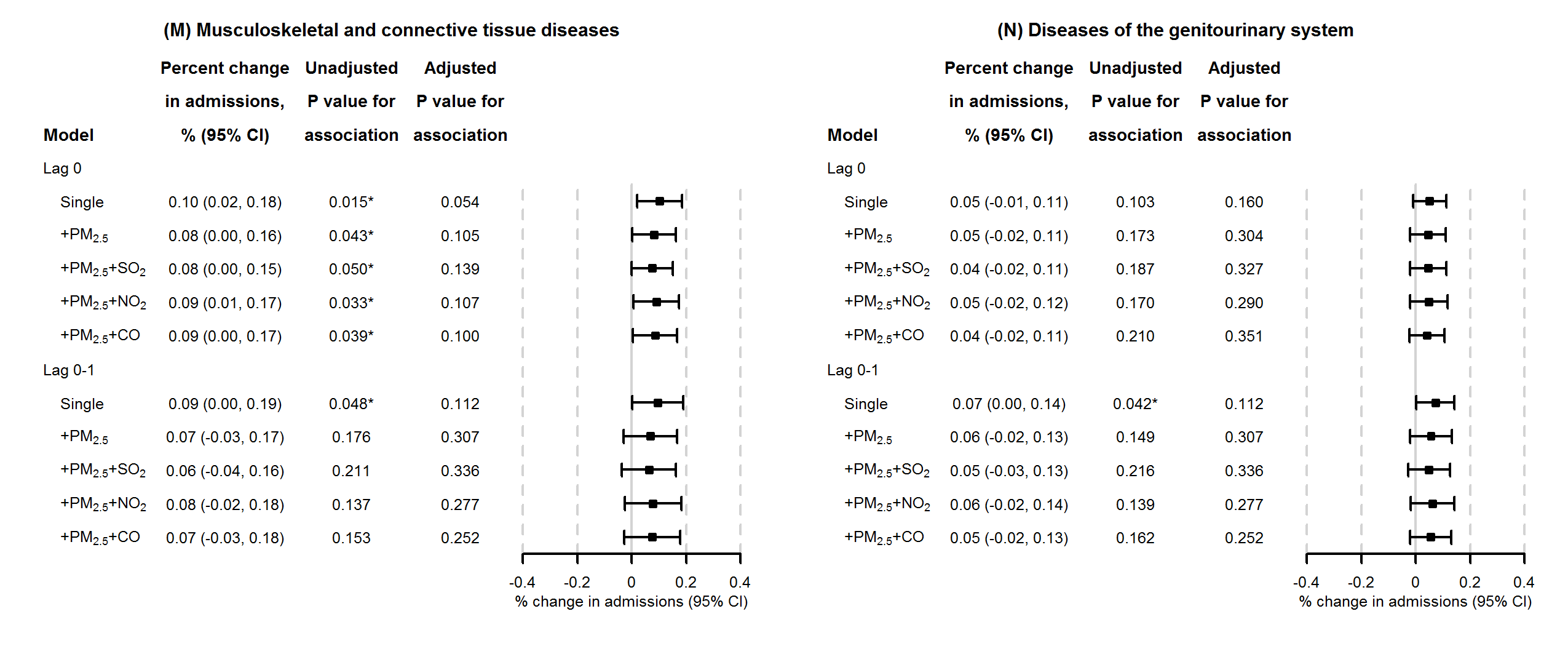


# S5 Fig. Percent change in hospital admissions per 10-μg/m^3^ increase in O_3_ by major disease categories using three-pollutant models, on average across all cities.

Results are presented as point estimates and 95% CIs of the percentage increase in daily hospital admissions associated with a 10-μg/m^3^ increase in O_3_. Major disease categories are based on the chapter division of the ICD-10 diagnostic coding system. “Lag 0” indicates that the single-day exposure on the same day was used as the exposure metric of O_3_. “Lag 0-1” indicates that the 2-day moving average exposure was used as the exposure metric of O_3_. In single-pollutant models, the effects of O_3_ were estimated without adjustment for co-pollutants; in two-pollutant models (denoted by “+PM_2.5_”), the effects of O_3_ were estimated after adjustment for PM_2.5_; in three-pollutant models (denoted by “+PM_2.5_+SO_2_”, “+PM_2.5_+NO_2_”, and “+PM_2.5_+CO”), the effects of O_3_ were estimated after adjustment for PM_2.5_ and one of SO_2_, NO_2_, and CO, respectively. The Benjamini-Hochberg procedure was applied to adjust the *P* values across 14 major disease categories; both unadjusted and adjusted *P* values are reported.

* Statistically significant estimate (*P* < 0.05).
