# Supplementary material for: Ambient air pollution and cause-specific risk of hospital admission in China: A nationwide time-series study
Source: PLoS Med. 2020 Aug 6;17(8):e1003188. doi: 10.1371/journal.pmed.1003188 (PMC7410211; doi:10.1371/journal.pmed.1003188)
Supplement: S7 Fig — (DOCX) [file pmed.1003188.s007.docx]

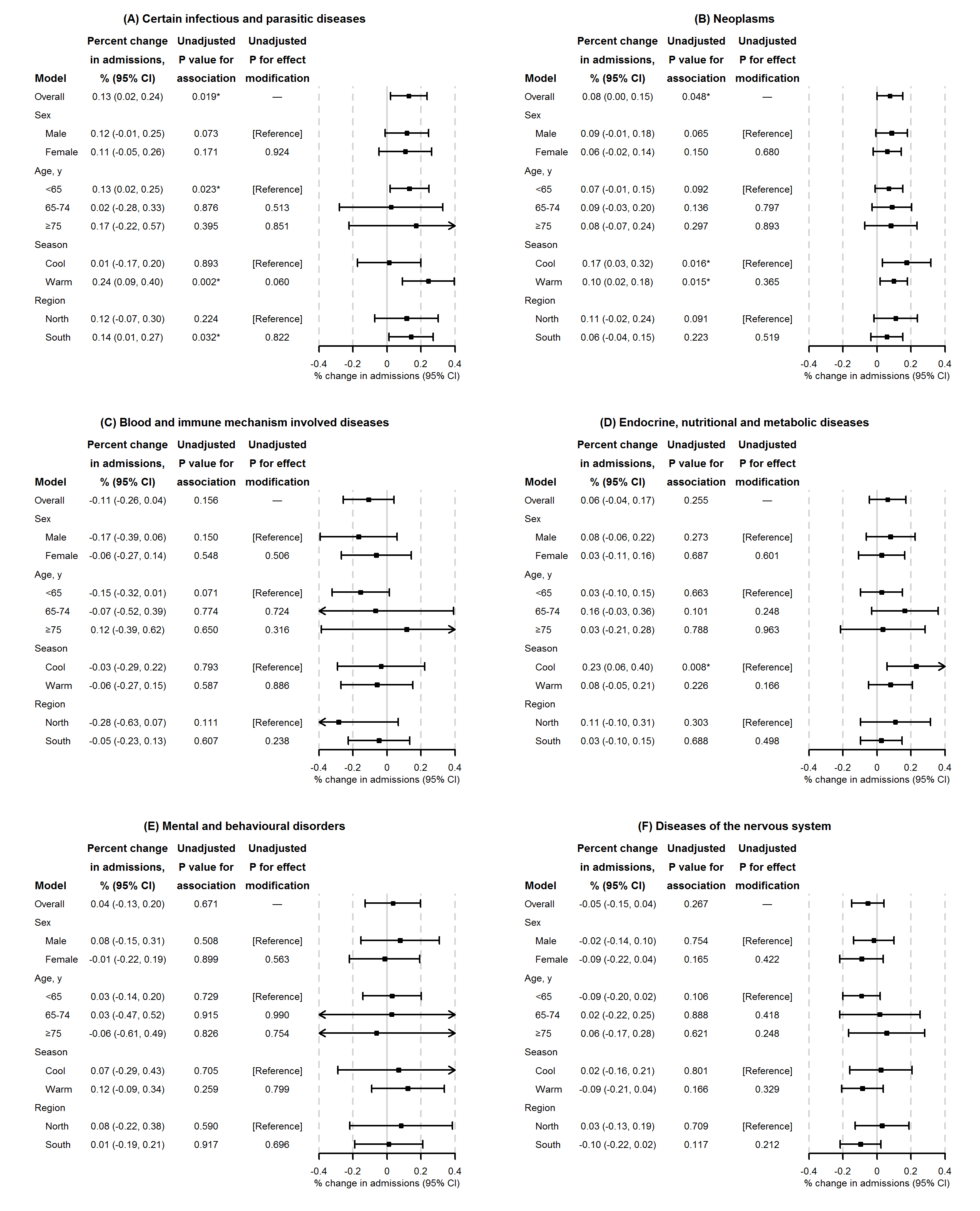


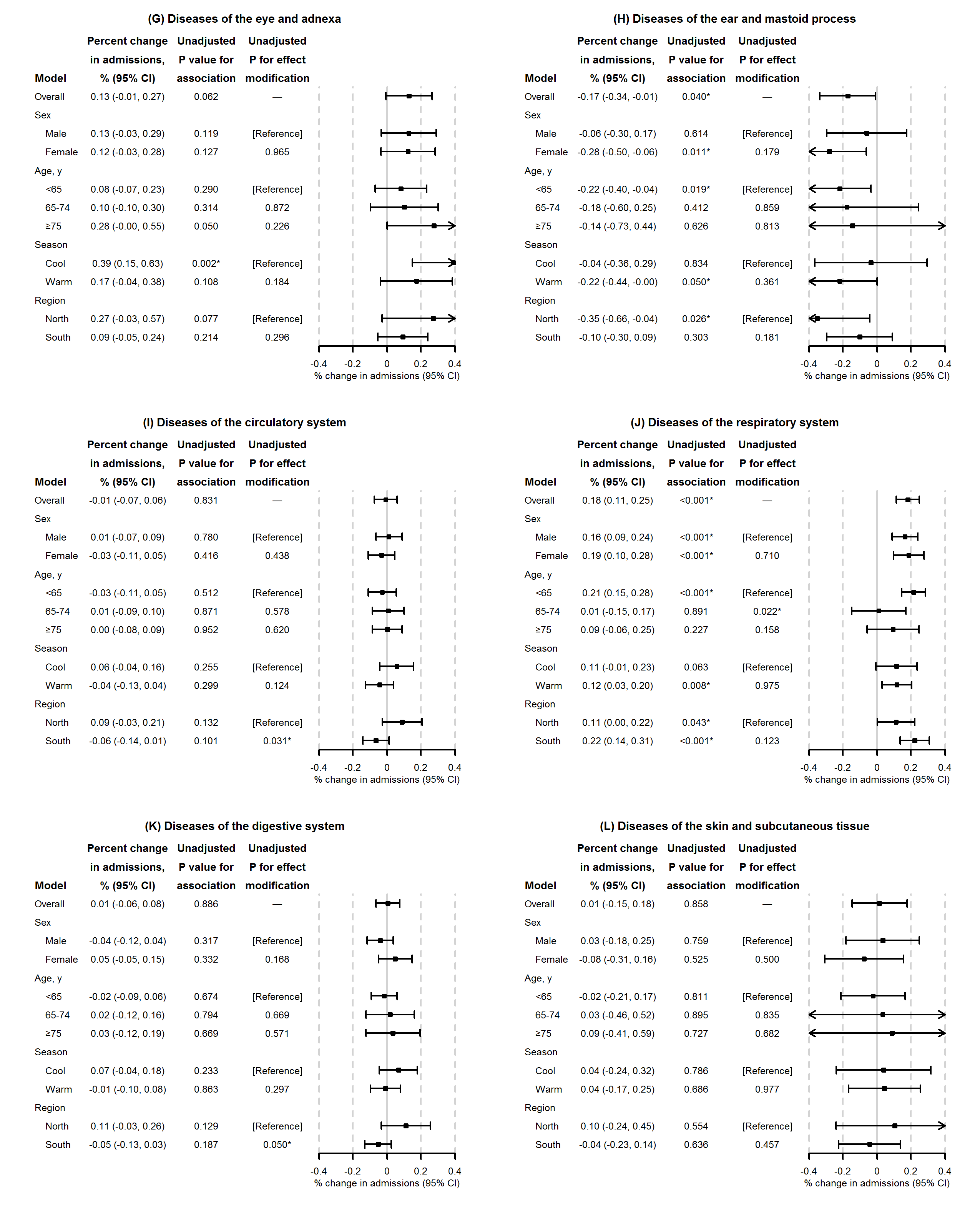


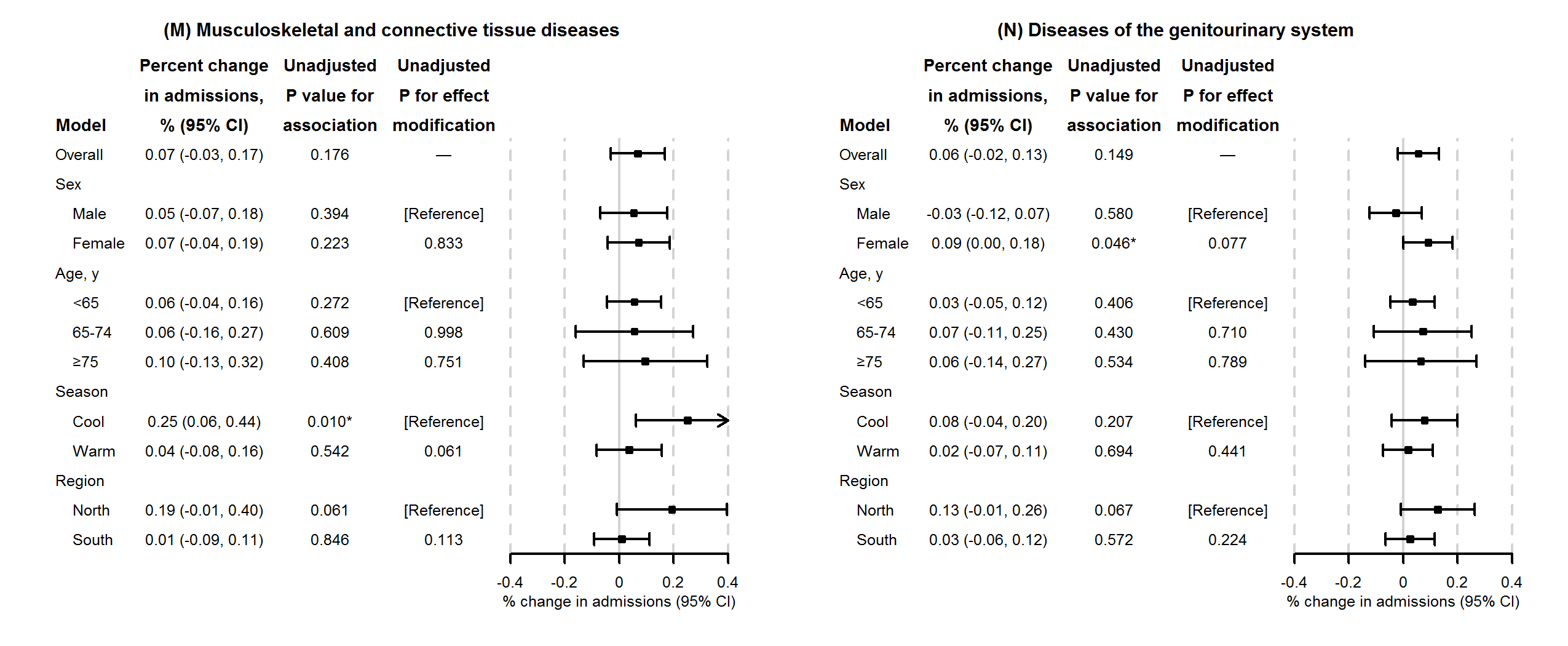


# S7 Fig. Percent change in hospital admissions per 10-μg/m^3^ increase in O_3_ by major disease categories according to study subgroups, on average across all cities.

Results are presented as point estimates and 95% CIs of the percentage increase in daily hospital admissions associated with a 10-μg/m^3^ increase in O_3_. Major disease categories are based on the chapter division of the ICD-10 diagnostic coding system. The 2-day moving average exposure (lag 0-1) was used as the exposure metric of O_3_. The effects of O_3_ were estimated after adjustment for PM_2.5_. The cool season is from October to next March; the warm season is from April to September. The two regions of China (North and South) are divided by the Huai River-Qinling Mountain line. The *P* values were not adjusted for multiple comparisons.

* Statistically significant estimate (*P* < 0.05).
