# Supplementary material for: Ambient air pollution and cause-specific risk of hospital admission in China: A nationwide time-series study
Source: PLoS Med. 2020 Aug 6;17(8):e1003188. doi: 10.1371/journal.pmed.1003188 (PMC7410211; doi:10.1371/journal.pmed.1003188)
Supplement: S9 Fig — (DOCX) [file pmed.1003188.s009.docx]

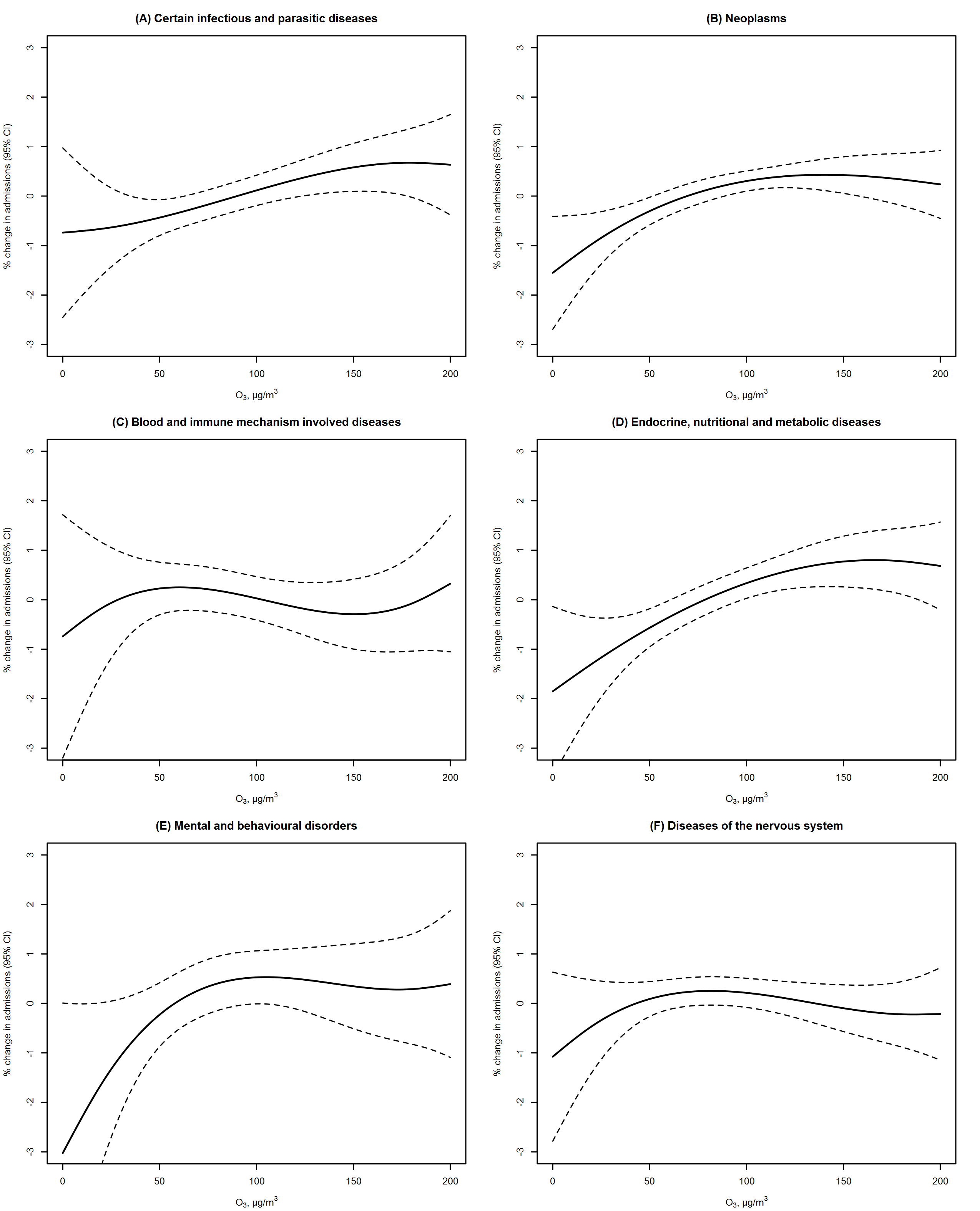


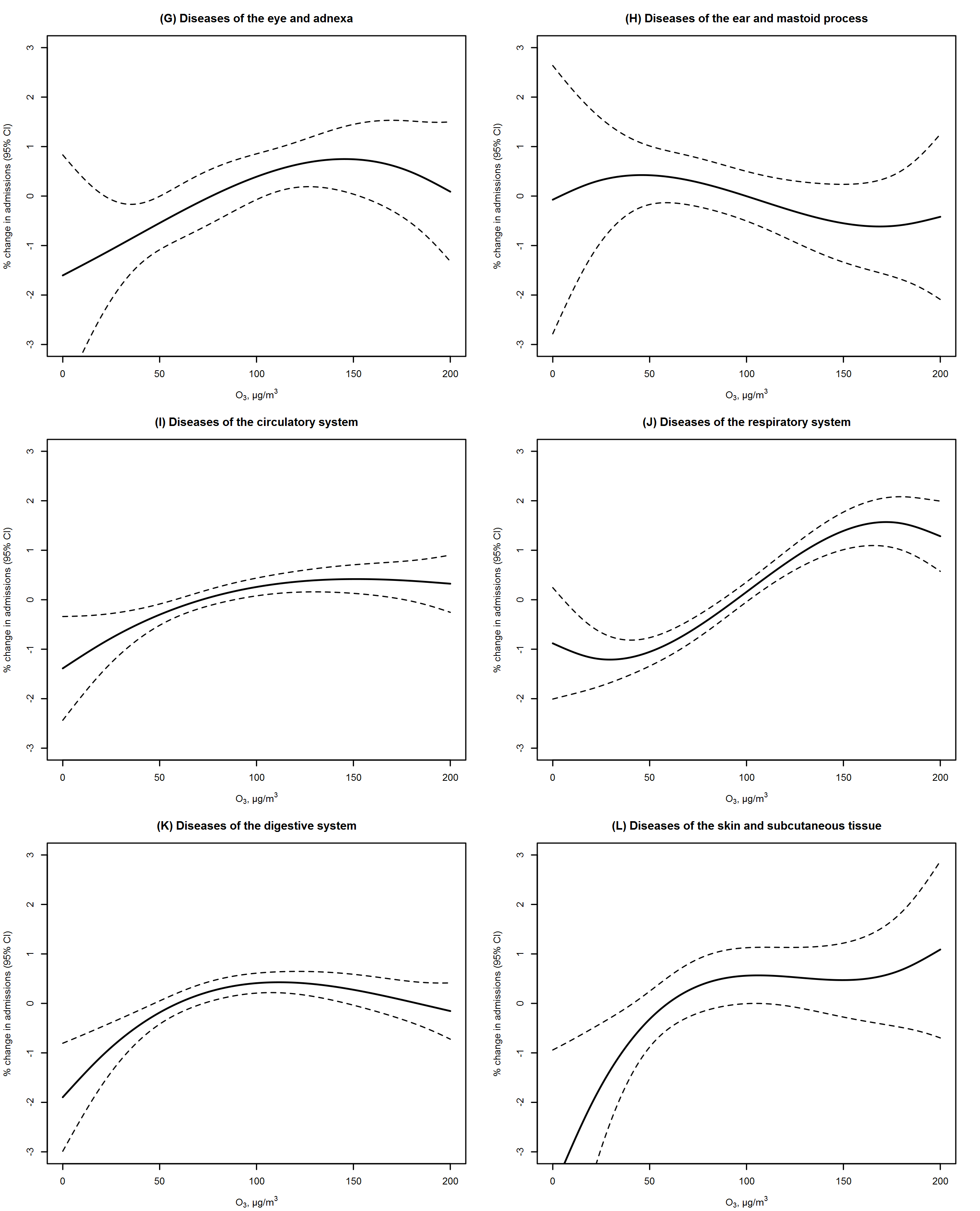


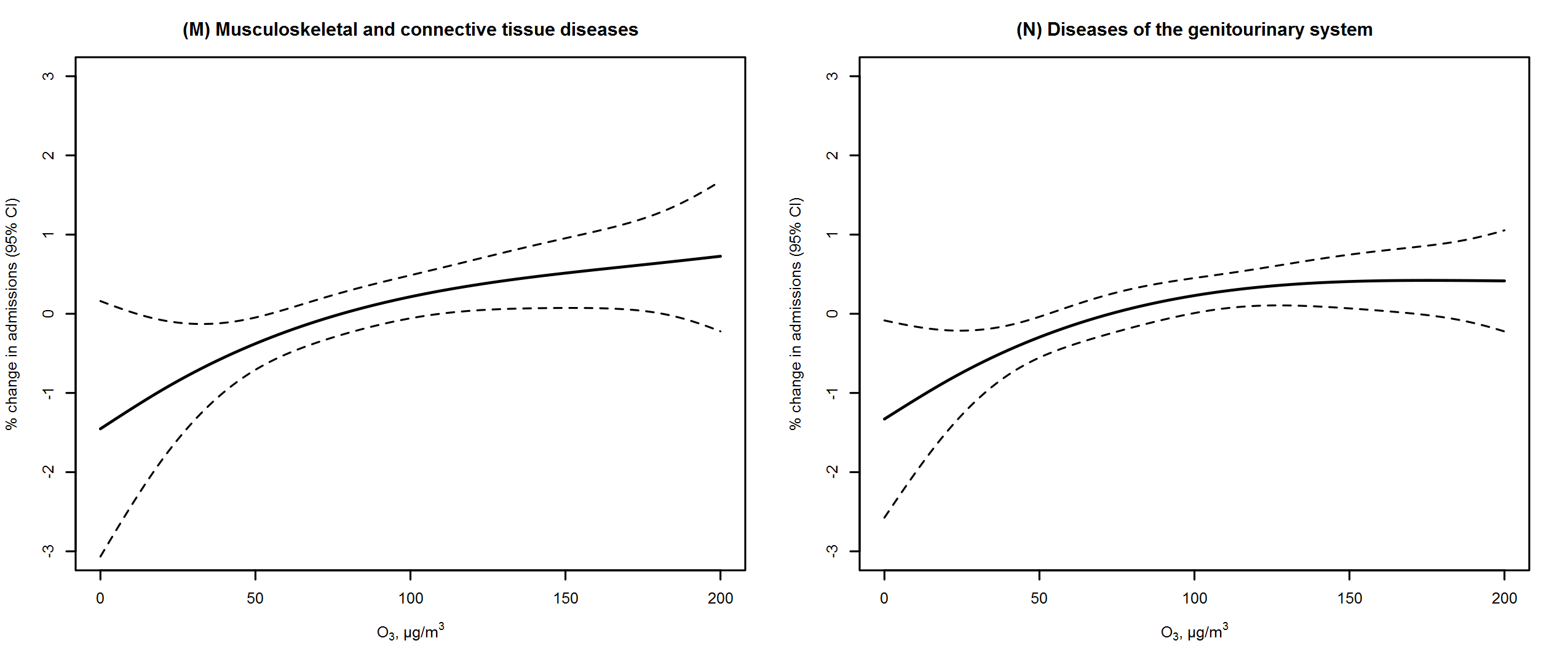


# S9 Fig. Exposure-response curves of the effects of O_3_ on hospital admissions by major disease categories, on average across all cities.

The vertical scale represents the relative change from the mean effect of O_3_ on daily hospital admissions, with solid lines indicating point estimates and dashed lines indicating 95% CIs. Major disease categories are based on the chapter division of the ICD-10 diagnostic coding system. The 2-day moving average exposure (lag 0-1) was used as the exposure metric of O_3_. The national average exposure-response curves were developed using multivariate meta-analysis approaches.
