# Supplementary material for: Ambient air pollution and cause-specific risk of hospital admission in China: A nationwide time-series study
Source: PLoS Med. 2020 Aug 6;17(8):e1003188. doi: 10.1371/journal.pmed.1003188 (PMC7410211; doi:10.1371/journal.pmed.1003188)
Supplement: S12 Fig — (DOCX) [file pmed.1003188.s012.docx]

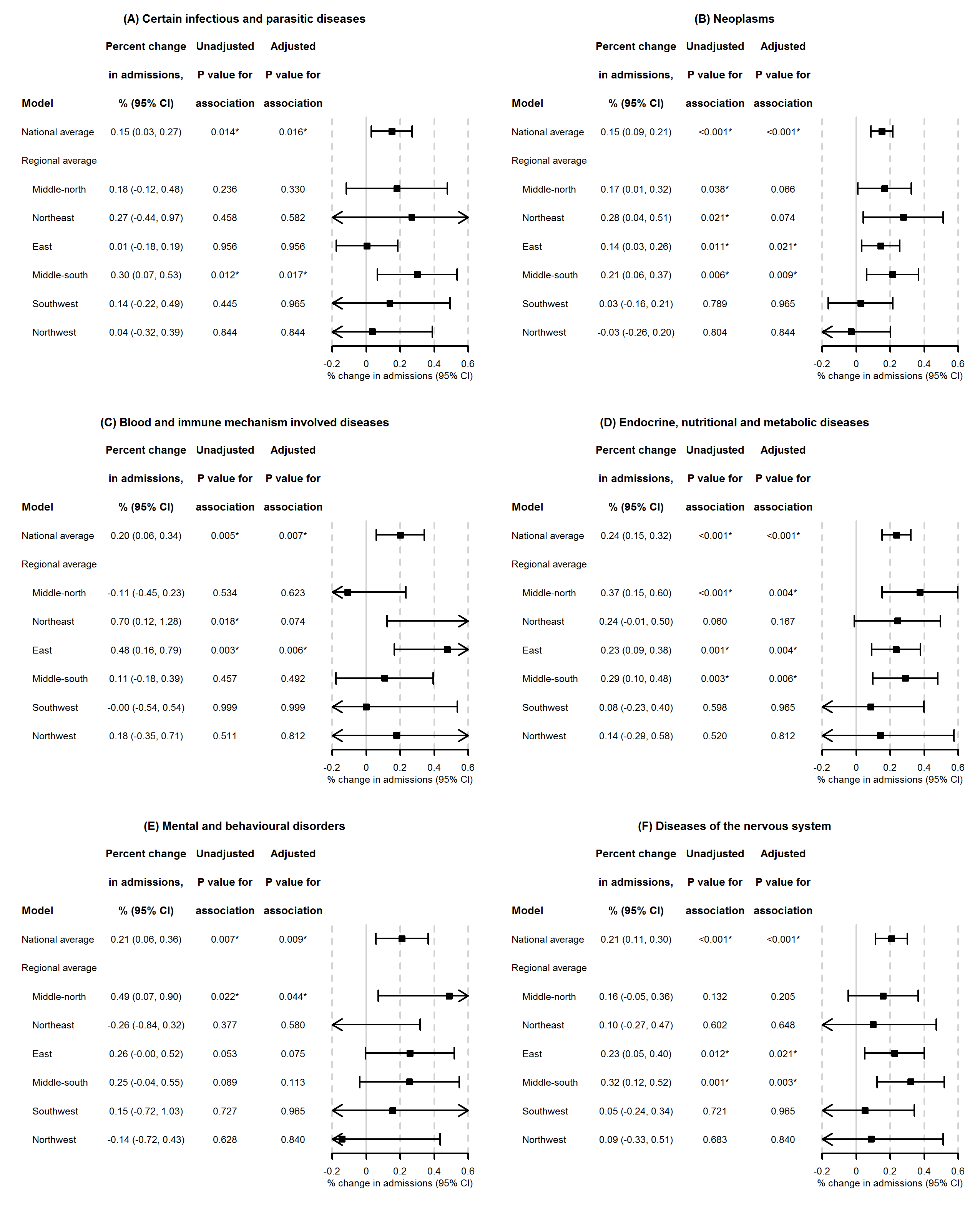


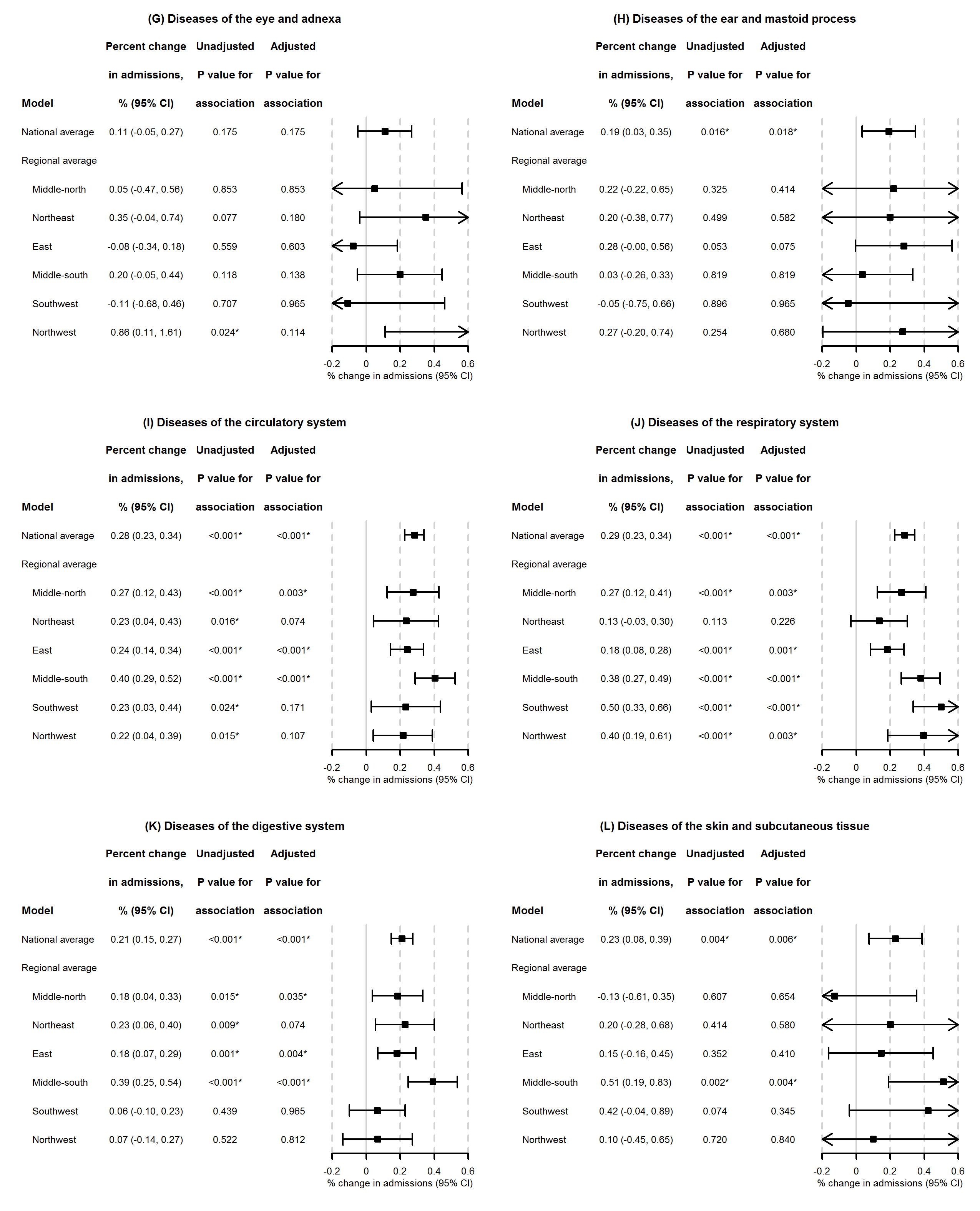


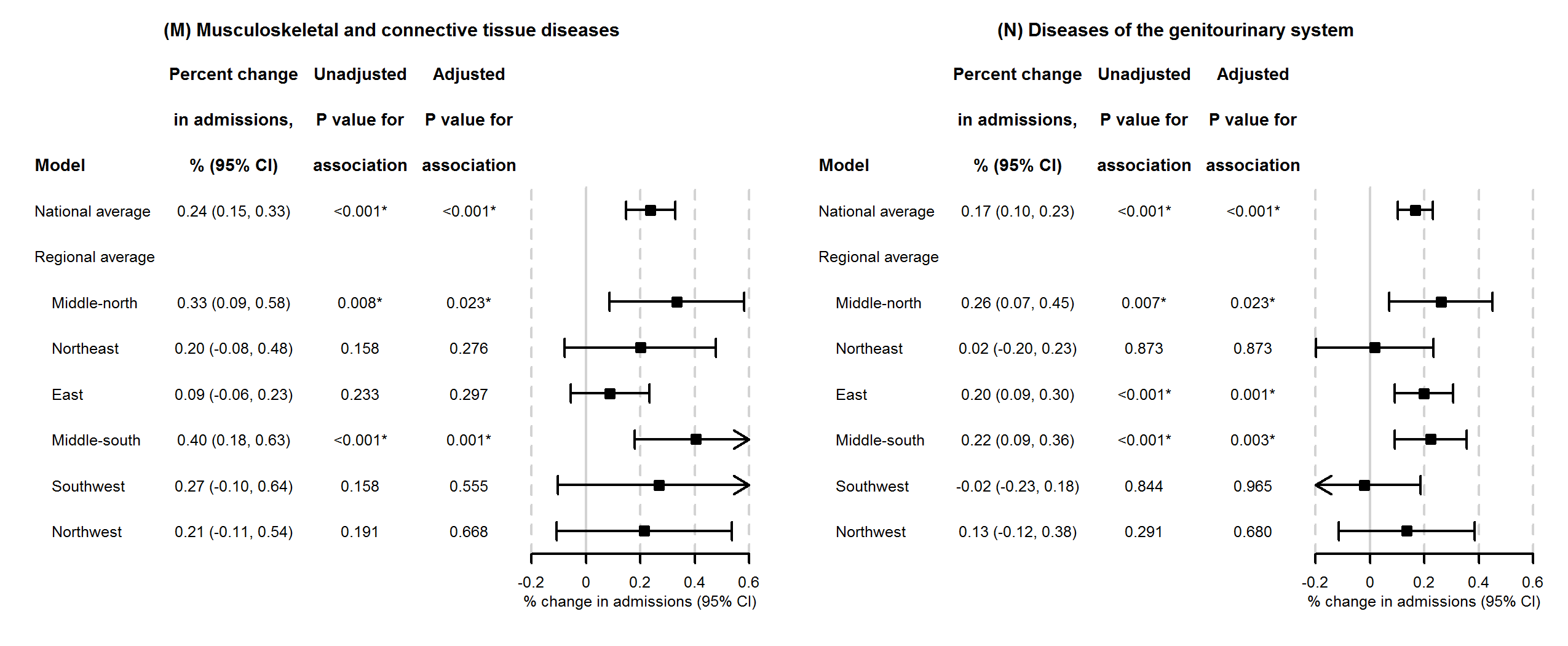


# S12 Fig. Percent change in hospital admissions per 10-μg/m^3^ increase in PM_2.5_ by major disease categories in six regions of China, on average across all cities.

Results are presented as point estimates and 95% CIs of the percentage increase in daily hospital admissions associated with a 10-μg/m^3^ increase in PM_2.5_. Major disease categories are based on the chapter division of the ICD-10 diagnostic coding system. The single-day exposure on the same day (lag 0) was used as the exposure metric of PM_2.5_. The effects of PM_2.5_ were estimated after adjustment for O_3_. The six regions of China (Middle-north, Northeast, East, Middle-south, Southwest, and Northwest) are illustrated in S1 Fig. The Benjamini-Hochberg procedure was applied to adjust the *P* values across 14 major disease categories; both unadjusted and adjusted *P* values are reported.

* Statistically significant estimate (*P* < 0.05).
