# Supplementary material for: Ambient air pollution and cause-specific risk of hospital admission in China: A nationwide time-series study
Source: PLoS Med. 2020 Aug 6;17(8):e1003188. doi: 10.1371/journal.pmed.1003188 (PMC7410211; doi:10.1371/journal.pmed.1003188)
Supplement: S1 Table — (DOCX) [file pmed.1003188.s015.docx]

# S1 Table. City-specific population and coverage rates of class 3 hospitals and hospital beds by the Hospital Quality Monitoring System (HQMS) in 252 Chinese cities.

|  |  |  |  | Class 3 hospital† | | |  | Hospital bed† | | |
| --- | --- | --- | --- | --- | --- | --- | --- | --- | --- | --- |
| City name |  | Population, thousand* |  | Total number | Covered number  by the HQMS | Coverage rate, % |  | Total number | Covered number  by the HQMS | Coverage rate, % |
| Beijing |  | 13,334 |  | 44 | 37 | 84.1 |  | 37,768 | 34,186 | 90.5 |
| Tianjin |  | 10,167 |  | 35 | 16 | 45.7 |  | 22,983 | 13,120 | 57.1 |
| Shijiazhuang |  | 10,249 |  | 14 | 8 | 57.1 |  | 15,489 | 10,502 | 67.8 |
| Tangshan |  | 7,532 |  | 6 | 3 | 50.0 |  | 5,742 | 3,522 | 61.3 |
| Qinhuangdao |  | 2,951 |  | 4 | 2 | 50.0 |  | 2,805 | 2,002 | 71.4 |
| Handan |  | 10,295 |  | 6 | 4 | 66.7 |  | 5,226 | 4,466 | 85.5 |
| Xingtai |  | 7,729 |  | 5 | 4 | 80.0 |  | 3,126 | 2,526 | 80.8 |
| Baoding |  | 11,966 |  | 8 | 5 | 62.5 |  | 5,500 | 3,800 | 69.1 |
| Zhangjiakou |  | 4,686 |  | 2 | 1 | 50.0 |  | 2,000 | 700 | 35.0 |
| Chengde |  | 3,807 |  | 2 | 2 | 100.0 |  | 2,450 | 2,450 | 100.0 |
| Cangzhou |  | 7,684 |  | 4 | 3 | 75.0 |  | 4,536 | 3,276 | 72.2 |
| Langfang |  | 4,504 |  | 4 | 1 | 25.0 |  | 2,237 | 818 | 36.6 |
| Hengshui |  | 4,526 |  | 2 | 1 | 50.0 |  | 1,546 | 960 | 62.1 |
| Taiyuan |  | 3,697 |  | 15 | 13 | 86.7 |  | 14,065 | 12,940 | 92.0 |
| Datong |  | 3,392 |  | 4 | 4 | 100.0 |  | 3,650 | 3,650 | 100.0 |
| Changzhi |  | 3,392 |  | 5 | 4 | 80.0 |  | 4,000 | 3,500 | 87.5 |
| Jincheng |  | 2,189 |  | 2 | 2 | 100.0 |  | 1,800 | 1,800 | 100.0 |
| Jinzhong |  | 3,305 |  | 3 | 2 | 66.7 |  | 1,602 | 1,100 | 68.7 |
| Yuncheng |  | 5,252 |  | 2 | 2 | 100.0 |  | 1,500 | 1,500 | 100.0 |
| Xinzhou |  | 3,125 |  | 1 | 1 | 100.0 |  | 600 | 600 | 100.0 |
| Linfen |  | 4,290 |  | 3 | 2 | 66.7 |  | 2,800 | 2,200 | 78.6 |
| Lvliang |  | 3,907 |  | 2 | 1 | 50.0 |  | 1,900 | 800 | 42.1 |
| Hohhot |  | 2,379 |  | 12 | 8 | 66.7 |  | 7,890 | 6,700 | 84.9 |
| Baotou |  | 2,237 |  | 11 | 9 | 81.8 |  | 6,388 | 5,563 | 87.1 |
| Wuhai |  | 554 |  | 1 | 1 | 100.0 |  | 500 | 500 | 100.0 |
| Chifeng |  | 4,658 |  | 5 | 4 | 80.0 |  | 4,480 | 3,580 | 79.9 |
| Tongliao |  | 3,194 |  | 3 | 2 | 66.7 |  | 1,900 | 1,900 | 100.0 |
| Ordos |  | 1,560 |  | 3 | 1 | 33.3 |  | 1,350 | 1,200 | 88.9 |
| Hulun Buir |  | 2,659 |  | 4 | 2 | 50.0 |  | 3,050 | 2,700 | 88.5 |
| Bayan Nur |  | 1,786 |  | 2 | 1 | 50.0 |  | 1,560 | 1,300 | 83.3 |
| Xilingol League |  | NA |  | 2 | 1 | 50.0 |  | 800 | 800 | 100.0 |
| Shenyang |  | 7,308 |  | 27 | 8 | 29.6 |  | 24,734 | 12,231 | 49.5 |
| Dalian |  | 5,943 |  | 18 | 5 | 27.8 |  | 14,780 | 7,250 | 49.1 |
| Anshan |  | 3,482 |  | 13 | 3 | 23.1 |  | 5,208 | 1,886 | 36.2 |
| Benxi |  | 1,520 |  | 3 | 1 | 33.3 |  | 2,705 | 1,200 | 44.4 |
| Jinzhou |  | 3,053 |  | 7 | 1 | 14.3 |  | 6,247 | 1,800 | 28.8 |
| Liaoyang |  | 1,799 |  | 2 | 2 | 100.0 |  | 2,200 | 2,200 | 100.0 |
| Chaoyang |  | 3,406 |  | 4 | 2 | 50.0 |  | 2,926 | 1,406 | 48.1 |
| Huludao |  | 2,807 |  | 2 | 1 | 50.0 |  | 1,058 | 400 | 37.8 |
| Changchun |  | 7,546 |  | 25 | 8 | 32.0 |  | 10,652 | 8,167 | 76.7 |
| Jilin |  | 4,277 |  | 6 | 2 | 33.3 |  | 3,727 | 2,473 | 66.4 |
| Tonghua |  | 2,222 |  | 4 | 1 | 25.0 |  | 2,652 | 1,002 | 37.8 |
| Yanbian Korean Autonomous Prefecture |  | NA |  | 2 | 2 | 100.0 |  | 1,374 | 1,374 | 100.0 |
| Harbin |  | 9,873 |  | 25 | 14 | 56.0 |  | 21,927 | 16,151 | 73.7 |
| Qiqihar |  | 5,532 |  | 6 | 1 | 16.7 |  | 6,560 | 2,600 | 39.6 |
| Jixi |  | 1,836 |  | 3 | 1 | 33.3 |  | 1,906 | 700 | 36.7 |
| Shuangyashan |  | 1,490 |  | 3 | 2 | 66.7 |  | 2,150 | 1,070 | 49.8 |
| Daqing |  | 2,760 |  | 6 | 3 | 50.0 |  | 5,035 | 2,935 | 58.3 |
| Yichun (in Heilongjiang Province) |  | 1,220 |  | 2 | 1 | 50.0 |  | 1,600 | 800 | 50.0 |
| Jiamusi |  | 2,414 |  | 8 | 3 | 37.5 |  | 6,280 | 2,400 | 38.2 |
| Qitaihe |  | 882 |  | 2 | 2 | 100.0 |  | 1,416 | 1,416 | 100.0 |
| Mudanjiang |  | 2,640 |  | 10 | 4 | 40.0 |  | 5,963 | 2,643 | 44.3 |
| Heihe |  | 1,705 |  | 4 | 3 | 75.0 |  | 2,402 | 1,901 | 79.1 |
| Daxing'anling Prefecture |  | NA |  | 1 | 1 | 100.0 |  | 500 | 500 | 100.0 |
| Shanghai |  | 14,387 |  | 39 | 30 | 76.9 |  | 31,788 | 27,948 | 87.9 |
| Nanjing |  | 6,487 |  | 21 | 20 | 95.2 |  | 17,203 | 17,123 | 99.5 |
| Wuxi |  | 4,771 |  | 8 | 8 | 100.0 |  | 7,100 | 7,100 | 100.0 |
| Xuzhou |  | 10,235 |  | 10 | 8 | 80.0 |  | 9,560 | 7,660 | 80.1 |
| Changzhou |  | 3,686 |  | 6 | 5 | 83.3 |  | 5,380 | 4,580 | 85.1 |
| Suzhou (in Jiangsu Province) |  | 6,611 |  | 13 | 12 | 92.3 |  | 11,917 | 11,117 | 93.3 |
| Nantong |  | 7,676 |  | 8 | 6 | 75.0 |  | 6,519 | 5,479 | 84.0 |
| Lianyungang |  | 5,265 |  | 4 | 4 | 100.0 |  | 3,394 | 3,394 | 100.0 |
| Huai'an |  | 5,603 |  | 6 | 6 | 100.0 |  | 4,118 | 4,118 | 100.0 |
| Yancheng |  | 8,285 |  | 6 | 5 | 83.3 |  | 5,450 | 4,900 | 89.9 |
| Yangzhou |  | 4,613 |  | 3 | 3 | 100.0 |  | 3,327 | 3,327 | 100.0 |
| Zhenjiang |  | 2,721 |  | 5 | 5 | 100.0 |  | 4,470 | 4,470 | 100.0 |
| Taizhou (in Jiangsu Province) |  | 5,085 |  | 2 | 2 | 100.0 |  | 2,718 | 2,718 | 100.0 |
| Suqian |  | 5,807 |  | 2 | 2 | 100.0 |  | 1,700 | 1,700 | 100.0 |
| Hangzhou |  | 7,158 |  | 25 | 13 | 52.0 |  | 21,535 | 14,310 | 66.4 |
| Ningbo |  | 5,838 |  | 13 | 3 | 23.1 |  | 11,086 | 3,566 | 32.2 |
| Wenzhou |  | 8,137 |  | 10 | 5 | 50.0 |  | 10,510 | 6,810 | 64.8 |
| Jiaxing |  | 3,481 |  | 6 | 3 | 50.0 |  | 3,600 | 2,400 | 66.7 |
| Huzhou |  | 2,638 |  | 4 | 3 | 75.0 |  | 3,070 | 2,570 | 83.7 |
| Shaoxing |  | 4,430 |  | 8 | 2 | 25.0 |  | 5,100 | 1,600 | 31.4 |
| Jinhua |  | 4,751 |  | 9 | 5 | 55.6 |  | 5,850 | 4,100 | 70.1 |
| Quzhou |  | 2,557 |  | 3 | 2 | 66.7 |  | 1,750 | 1,250 | 71.4 |
| Zhoushan |  | 975 |  | 3 | 1 | 33.3 |  | 1,700 | 500 | 29.4 |
| Taizhou (in Zhejiang Province) |  | 5,971 |  | 6 | 3 | 50.0 |  | 4,900 | 2,500 | 51.0 |
| Lishui |  | 2,657 |  | 4 | 2 | 50.0 |  | 3,000 | 1,900 | 63.3 |
| Hefei |  | 7,128 |  | 12 | 11 | 91.7 |  | 11,930 | 11,730 | 98.3 |
| Wuhu |  | 3,845 |  | 4 | 1 | 25.0 |  | 3,548 | 1,500 | 42.3 |
| Bengbu |  | 3,711 |  | 4 | 4 | 100.0 |  | 2,948 | 2,948 | 100.0 |
| Huainan |  | 2,434 |  | 2 | 1 | 50.0 |  | 1,800 | 1,000 | 55.6 |
| Ma'anshan |  | 2,272 |  | 2 | 2 | 100.0 |  | 1,375 | 1,375 | 100.0 |
| Huaibei |  | 2,153 |  | 2 | 2 | 100.0 |  | 2,287 | 2,287 | 100.0 |
| Anqing |  | 6,209 |  | 2 | 2 | 100.0 |  | 2,650 | 2,650 | 100.0 |
| Chuzhou |  | 4,496 |  | 1 | 1 | 100.0 |  | 1,000 | 1,000 | 100.0 |
| Fuyang |  | 10,514 |  | 3 | 3 | 100.0 |  | 2,950 | 2,950 | 100.0 |
| Suzhou (in Anhui Province) |  | 6,423 |  | 2 | 2 | 100.0 |  | 2,000 | 2,000 | 100.0 |
| Lu'an |  | 7,205 |  | 1 | 1 | 100.0 |  | 1,100 | 1,100 | 100.0 |
| Bozhou |  | 6,344 |  | 1 | 1 | 100.0 |  | 860 | 860 | 100.0 |
| Xuancheng |  | 2,798 |  | 1 | 1 | 100.0 |  | 800 | 800 | 100.0 |
| Fuzhou (in Fujian Province) |  | 6,749 |  | 21 | 13 | 61.9 |  | 19,070 | 14,815 | 77.7 |
| Xiamen |  | 2,034 |  | 16 | 13 | 81.2 |  | 12,691 | 10,011 | 78.9 |
| Putian |  | 3,412 |  | 2 | 2 | 100.0 |  | 2,100 | 2,100 | 100.0 |
| Quanzhou |  | 7,162 |  | 11 | 5 | 45.5 |  | 7,570 | 3,930 | 51.9 |
| Zhangzhou |  | 4,974 |  | 2 | 1 | 50.0 |  | 2,560 | 2,560 | 100.0 |
| Nanping |  | 3,192 |  | 3 | 2 | 66.7 |  | 2,300 | 1,800 | 78.3 |
| Longyan |  | 3,071 |  | 4 | 4 | 100.0 |  | 2,825 | 2,825 | 100.0 |
| Ningde |  | 3,522 |  | 4 | 3 | 75.0 |  | 3,670 | 3,310 | 90.2 |
| Nanchang |  | 5,177 |  | 19 | 16 | 84.2 |  | 13,820 | 12,240 | 88.6 |
| Jingdezhen |  | 1,678 |  | 3 | 3 | 100.0 |  | 1,728 | 1,728 | 100.0 |
| Pingxiang |  | 1,982 |  | 3 | 3 | 100.0 |  | 2,613 | 2,613 | 100.0 |
| Jiujiang |  | 5,131 |  | 4 | 4 | 100.0 |  | 2,738 | 2,738 | 100.0 |
| Xinyu |  | 1,223 |  | 2 | 2 | 100.0 |  | 1,550 | 1,550 | 100.0 |
| Yingtan |  | 1,269 |  | 1 | 1 | 100.0 |  | 750 | 750 | 100.0 |
| Ganzhou |  | 9,542 |  | 6 | 6 | 100.0 |  | 4,820 | 4,820 | 100.0 |
| Ji'an |  | 5,267 |  | 3 | 3 | 100.0 |  | 2,595 | 2,595 | 100.0 |
| Yichun (in Jiangxi Province) |  | 5,956 |  | 4 | 3 | 75.0 |  | 2,680 | 2,230 | 83.2 |
| Fuzhou (in Jiangxi Province) |  | 4,275 |  | 1 | 1 | 100.0 |  | 522 | 522 | 100.0 |
| Shangrao |  | 7,731 |  | 2 | 2 | 100.0 |  | 1,327 | 1,327 | 100.0 |
| Jinan |  | 6,216 |  | 18 | 9 | 50.0 |  | 17,363 | 14,281 | 82.2 |
| Qingdao |  | 7,806 |  | 13 | 4 | 30.8 |  | 7,290 | 3,373 | 46.3 |
| Zibo |  | 4,280 |  | 7 | 2 | 28.6 |  | 3,717 | 2,200 | 59.2 |
| ZaoZhuang |  | 4,013 |  | 5 | 1 | 20.0 |  | 3,208 | 1,100 | 34.3 |
| Dongying |  | 1,891 |  | 3 | 2 | 66.7 |  | 2,935 | 2,135 | 72.7 |
| Weifang |  | 8,883 |  | 5 | 2 | 40.0 |  | 4,008 | 1,983 | 49.5 |
| Jining |  | 8,601 |  | 4 | 2 | 50.0 |  | 7,088 | 4,288 | 60.5 |
| Tai'an |  | 5,623 |  | 4 | 1 | 25.0 |  | 4,044 | 1,866 | 46.1 |
| Weihai |  | 2,548 |  | 3 | 3 | 100.0 |  | 2,400 | 2,400 | 100.0 |
| Dezhou |  | 5,832 |  | 2 | 1 | 50.0 |  | 1,860 | 1,300 | 69.9 |
| Binzhou |  | 3,867 |  | 3 | 2 | 66.7 |  | 3,945 | 3,445 | 87.3 |
| Heze |  | 9,906 |  | 3 | 2 | 66.7 |  | 2,200 | 2,200 | 100.0 |
| Zhengzhou |  | 9,378 |  | 17 | 15 | 88.2 |  | 23,110 | 23,110 | 100.0 |
| Kaifeng |  | 5,538 |  | 5 | 1 | 20.0 |  | 4,680 | 1,000 | 21.4 |
| Luoyang |  | 6,962 |  | 8 | 6 | 75.0 |  | 6,610 | 5,500 | 83.2 |
| Pingdingshan |  | 5,571 |  | 3 | 2 | 66.7 |  | 3,000 | 1,800 | 60.0 |
| Anyang |  | 6,114 |  | 6 | 3 | 50.0 |  | 4,931 | 2,881 | 58.4 |
| Hebi |  | 1,669 |  | 1 | 1 | 100.0 |  | 820 | 820 | 100.0 |
| Xinxiang |  | 6,305 |  | 6 | 5 | 83.3 |  | 7,607 | 7,607 | 100.0 |
| Puyang |  | 4,245 |  | 2 | 2 | 100.0 |  | 2,400 | 2,400 | 100.0 |
| Xuchang |  | 4,998 |  | 2 | 1 | 50.0 |  | 2,500 | 1,500 | 60.0 |
| Luohe |  | 2,667 |  | 2 | 2 | 100.0 |  | 2,055 | 2,055 | 100.0 |
| Sanmenxia |  | 2,278 |  | 2 | 2 | 100.0 |  | 2,200 | 2,200 | 100.0 |
| Nanyang |  | 11,814 |  | 6 | 5 | 83.3 |  | 7,050 | 6,450 | 91.5 |
| Shangqiu |  | 9,497 |  | 1 | 1 | 100.0 |  | 1,400 | 1,400 | 100.0 |
| Xinyang |  | 8,904 |  | 1 | 1 | 100.0 |  | 1,200 | 1,200 | 100.0 |
| Zhoukou |  | 12,369 |  | 1 | 1 | 100.0 |  | 1,350 | 1,350 | 100.0 |
| Zhumadian |  | 9,206 |  | 3 | 2 | 66.7 |  | 3,800 | 2,900 | 76.3 |
| Wuhan |  | 8,273 |  | 34 | 33 | 97.1 |  | 47,997 | 46,071 | 96.0 |
| Huangshi |  | 2,651 |  | 5 | 3 | 60.0 |  | 3,798 | 2,688 | 70.8 |
| Shiyan |  | 3,470 |  | 5 | 3 | 60.0 |  | 6,600 | 5,650 | 85.6 |
| Yichang |  | 4,004 |  | 8 | 6 | 75.0 |  | 6,910 | 6,310 | 91.3 |
| Xiangyang |  | 5,955 |  | 5 | 5 | 100.0 |  | 5,850 | 5,850 | 100.0 |
| Ezhou |  | 1,102 |  | 4 | 1 | 25.0 |  | 1,943 | 900 | 46.3 |
| Jingmen |  | 3,003 |  | 5 | 4 | 80.0 |  | 5,270 | 4,970 | 94.3 |
| Xiaogan |  | 5,257 |  | 4 | 2 | 50.0 |  | 3,125 | 2,400 | 76.8 |
| Jingzhou |  | 6,585 |  | 8 | 6 | 75.0 |  | 8,060 | 6,500 | 80.6 |
| Huanggang |  | 7,414 |  | 5 | 3 | 60.0 |  | 3,550 | 2,250 | 63.4 |
| Xianning |  | 2,965 |  | 1 | 1 | 100.0 |  | 1,000 | 1,000 | 100.0 |
| Suizhou |  | 2,571 |  | 4 | 2 | 50.0 |  | 2,430 | 1,800 | 74.1 |
| Enshi Tujia and Miao Autonomous Prefecture |  | NA |  | 3 | 3 | 100.0 |  | 3,500 | 3,500 | 100.0 |
| Xiantao |  | NA |  | 1 | 1 | 100.0 |  | 2,200 | 2,200 | 100.0 |
| Qianjiang |  | NA |  | 2 | 2 | 100.0 |  | 1,770 | 1,770 | 100.0 |
| Tianmen |  | NA |  | 1 | 1 | 100.0 |  | 940 | 940 | 100.0 |
| Changsha |  | 6,714 |  | 19 | 9 | 47.4 |  | 23,900 | 15,270 | 63.9 |
| Zhuzhou |  | 3,961 |  | 4 | 2 | 50.0 |  | 2,796 | 1,610 | 57.6 |
| Xiangtan |  | 2,915 |  | 3 | 3 | 100.0 |  | 2,134 | 2,134 | 100.0 |
| Hengyang |  | 7,915 |  | 6 | 3 | 50.0 |  | 6,765 | 4,115 | 60.8 |
| Yueyang |  | 5,633 |  | 3 | 2 | 66.7 |  | 2,479 | 1,050 | 42.4 |
| Zhangjiajie |  | 1,721 |  | 1 | 1 | 100.0 |  | 800 | 800 | 100.0 |
| Chenzhou |  | 5,188 |  | 1 | 1 | 100.0 |  | 2,000 | 2,000 | 100.0 |
| Guangzhou |  | 8,424 |  | 31 | 29 | 93.5 |  | 30,816 | 29,183 | 94.7 |
| Shenzhen |  | 3,322 |  | 21 | 19 | 90.5 |  | 16,520 | 14,420 | 87.3 |
| Zhuhai |  | 1,102 |  | 3 | 2 | 66.7 |  | 3,300 | 2,300 | 69.7 |
| Shantou |  | 5,466 |  | 7 | 5 | 71.4 |  | 5,730 | 4,230 | 73.8 |
| Foshan |  | 3,856 |  | 8 | 6 | 75.0 |  | 6,776 | 5,576 | 82.3 |
| Jiangmen |  | 3,934 |  | 3 | 3 | 100.0 |  | 5,700 | 5,700 | 100.0 |
| Zhanjiang |  | 8,190 |  | 3 | 2 | 66.7 |  | 4,306 | 3,406 | 79.1 |
| Maoming |  | 7,724 |  | 2 | 2 | 100.0 |  | 3,600 | 3,600 | 100.0 |
| Huizhou |  | 3,485 |  | 1 | 1 | 100.0 |  | 1,500 | 1,500 | 100.0 |
| Shanwei |  | 3,591 |  | 1 | 1 | 100.0 |  | 550 | 550 | 100.0 |
| Yangjiang |  | 2,894 |  | 1 | 1 | 100.0 |  | 1,200 | 1,200 | 100.0 |
| Qingyuan |  | 4,123 |  | 1 | 1 | 100.0 |  | 1,420 | 1,420 | 100.0 |
| Dongguan |  | 1,914 |  | 6 | 6 | 100.0 |  | 7,191 | 7,191 | 100.0 |
| Chaozhou |  | 2,688 |  | 2 | 2 | 100.0 |  | 2,000 | 2,000 | 100.0 |
| Jieyang |  | 6,942 |  | 3 | 1 | 33.3 |  | 2,820 | 1,100 | 39.0 |
| Nanning |  | 7,297 |  | 12 | 8 | 66.7 |  | 8,763 | 7,983 | 91.1 |
| Liuzhou |  | 3,779 |  | 7 | 5 | 71.4 |  | 5,404 | 5,004 | 92.6 |
| Guilin |  | 5,265 |  | 4 | 4 | 100.0 |  | 2,957 | 2,957 | 100.0 |
| Wuzhou |  | 3,403 |  | 4 | 3 | 75.0 |  | 2,381 | 1,881 | 79.0 |
| Beihai |  | 1,693 |  | 1 | 1 | 100.0 |  | 1,050 | 1,050 | 100.0 |
| Qinzhou |  | 4,020 |  | 2 | 1 | 50.0 |  | 1,961 | 606 | 30.9 |
| Yulin |  | 7,080 |  | 2 | 2 | 100.0 |  | 1,694 | 1,694 | 100.0 |
| Baise |  | 4,120 |  | 2 | 2 | 100.0 |  | 2,240 | 2,240 | 100.0 |
| Hechi |  | 4,199 |  | 2 | 1 | 50.0 |  | 1,505 | 1,000 | 66.4 |
| Haikou |  | 1,653 |  | 6 | 6 | 100.0 |  | 9,616 | 8,416 | 87.5 |
| Sanya |  | 586 |  | 2 | 2 | 100.0 |  | 2,000 | 2,000 | 100.0 |
| Danzhou |  | NA |  | 2 | 2 | 100.0 |  | 2,000 | 2,000 | 100.0 |
| Chongqing |  | 33,752 |  | 14 | 12 | 85.7 |  | 16,105 | 12,635 | 78.5 |
| Chengdu |  | 12,107 |  | 33 | 18 | 54.5 |  | 24,268 | 17,347 | 71.5 |
| Zigong |  | 3,300 |  | 5 | 5 | 100.0 |  | 5,860 | 5,860 | 100.0 |
| Panzhihua |  | 1,119 |  | 5 | 4 | 80.0 |  | 3,250 | 2,800 | 86.2 |
| Luzhou |  | 5,089 |  | 4 | 3 | 75.0 |  | 2,890 | 2,890 | 100.0 |
| Deyang |  | 3,925 |  | 5 | 4 | 80.0 |  | 3,320 | 2,820 | 84.9 |
| Mianyang |  | 5,488 |  | 11 | 10 | 90.9 |  | 7,302 | 7,302 | 100.0 |
| Guangyuan |  | 3,101 |  | 6 | 5 | 83.3 |  | 5,400 | 4,800 | 88.9 |
| Suining |  | 3,804 |  | 3 | 3 | 100.0 |  | 2,000 | 2,000 | 100.0 |
| Neijiang |  | 4,260 |  | 5 | 3 | 60.0 |  | 3,602 | 2,699 | 74.9 |
| Leshan |  | 3,557 |  | 4 | 4 | 100.0 |  | 3,298 | 3,298 | 100.0 |
| Nanchong |  | 7,590 |  | 5 | 3 | 60.0 |  | 6,482 | 4,750 | 73.3 |
| Meishan |  | 3,530 |  | 3 | 1 | 33.3 |  | 2,920 | 800 | 27.4 |
| Yibin |  | 5,543 |  | 2 | 2 | 100.0 |  | 2,915 | 2,915 | 100.0 |
| Guang'an |  | 4,717 |  | 2 | 1 | 50.0 |  | 1,400 | 800 | 57.1 |
| Dazhou |  | 6,881 |  | 4 | 3 | 75.0 |  | 4,650 | 4,200 | 90.3 |
| Ya'an |  | 1,572 |  | 1 | 1 | 100.0 |  | 800 | 800 | 100.0 |
| Ziyang |  | 5,073 |  | 4 | 3 | 75.0 |  | 3,405 | 2,906 | 85.3 |
| Ngawa Tibetan and Qiang Autonomous Prefecture |  | NA |  | 1 | 1 | 100.0 |  | 600 | 600 | 100.0 |
| Liangshan Yi Autonomous Prefecture |  | NA |  | 3 | 1 | 33.3 |  | 1,434 | 600 | 41.8 |
| Guiyang |  | 3,829 |  | 24 | 8 | 33.3 |  | 15,755 | 8,236 | 52.3 |
| Liupanshui |  | 3,283 |  | 6 | 1 | 16.7 |  | 5,151 | 800 | 15.5 |
| Zunyi |  | 7,870 |  | 8 | 3 | 37.5 |  | 6,762 | 3,827 | 56.6 |
| Bijie |  | 8,808 |  | 1 | 1 | 100.0 |  | 800 | 800 | 100.0 |
| Tongren |  | 4,323 |  | 5 | 2 | 40.0 |  | 3,100 | 1,600 | 51.6 |
| Qianxinan Buyi and Miao Autonomous Prefecture |  | NA |  | 2 | 2 | 100.0 |  | 2,300 | 2,300 | 100.0 |
| Qiandongnan Miao and Dong Autonomous Prefecture |  | NA |  | 2 | 2 | 100.0 |  | 1,835 | 1,835 | 100.0 |
| Kunming |  | 5,505 |  | 21 | 12 | 57.1 |  | 14,273 | 12,694 | 88.9 |
| Qujing |  | 6,465 |  | 4 | 3 | 75.0 |  | 3,580 | 3,080 | 86.0 |
| Yuxi |  | 2,160 |  | 3 | 3 | 100.0 |  | 1,951 | 1,951 | 100.0 |
| Baoshan |  | 2,588 |  | 2 | 2 | 100.0 |  | 1,410 | 1,410 | 100.0 |
| Zhaotong |  | 5,944 |  | 2 | 2 | 100.0 |  | 2,700 | 2,700 | 100.0 |
| Lijiang |  | 1,212 |  | 1 | 1 | 100.0 |  | 800 | 800 | 100.0 |
| Pu'er |  | 2,538 |  | 2 | 1 | 50.0 |  | 1,600 | 1,200 | 75.0 |
| Lincang |  | 2,378 |  | 1 | 1 | 100.0 |  | 1,060 | 1,060 | 100.0 |
| Chuxiong Yi Autonomous Prefecture |  | NA |  | 2 | 1 | 50.0 |  | 1,310 | 810 | 61.8 |
| Honghe Hani and Yi Autonomous Prefecture |  | NA |  | 3 | 3 | 100.0 |  | 1,950 | 1,950 | 100.0 |
| Wenshan Zhuang and Miao Autonomous Prefecture |  | NA |  | 2 | 2 | 100.0 |  | 1,350 | 1,350 | 100.0 |
| Xishuangbanna Dai Autonomous Prefecture |  | NA |  | 2 | 2 | 100.0 |  | 1,540 | 1,540 | 100.0 |
| Dali Bai Autonomous Prefecture |  | NA |  | 2 | 2 | 100.0 |  | 2,085 | 2,085 | 100.0 |
| Dehong Dai and Jingpo Autonomous Prefecture |  | NA |  | 1 | 1 | 100.0 |  | 930 | 930 | 100.0 |
| Nujiang Lisu Autonomous Prefecture |  | NA |  | 1 | 1 | 100.0 |  | 200 | 200 | 100.0 |
| Xi'an |  | 8,153 |  | 23 | 14 | 60.9 |  | 18,919 | 14,439 | 76.3 |
| Baoji |  | 3,838 |  | 3 | 3 | 100.0 |  | 2,318 | 2,318 | 100.0 |
| Xianyang |  | 5,267 |  | 4 | 3 | 75.0 |  | 3,448 | 2,548 | 73.9 |
| Weinan |  | 5,614 |  | 1 | 1 | 100.0 |  | 1,000 | 1,000 | 100.0 |
| Yan'an |  | 2,343 |  | 2 | 2 | 100.0 |  | 2,600 | 2,600 | 100.0 |
| Hanzhong |  | 3,841 |  | 2 | 2 | 100.0 |  | 1,520 | 1,520 | 100.0 |
| Ankang |  | 3,062 |  | 2 | 1 | 50.0 |  | 2,200 | 1,200 | 54.5 |
| Lanzhou |  | 3,747 |  | 11 | 7 | 63.6 |  | 14,074 | 10,112 | 71.8 |
| Jiayuguan |  | 241 |  | 2 | 1 | 50.0 |  | 606 | 606 | 100.0 |
| Jinchang |  | 470 |  | 1 | 1 | 100.0 |  | 616 | 616 | 100.0 |
| Wuwei |  | 1,889 |  | 5 | 2 | 40.0 |  | 3,100 | 1,400 | 45.2 |
| Jiuquan |  | 1,112 |  | 1 | 1 | 100.0 |  | 680 | 680 | 100.0 |
| Xining |  | 2,026 |  | 15 | 10 | 66.7 |  | 10,442 | 7,803 | 74.7 |
| Haixi Mongolian and Tibetan Autonomous Prefecture |  | NA |  | 2 | 2 | 100.0 |  | 1,003 | 1,003 | 100.0 |
| Yinchuan |  | 1,960 |  | 3 | 2 | 66.7 |  | 3,600 | 2,800 | 77.8 |
| Urumqi |  | 2,669 |  | 11 | 7 | 63.6 |  | 10,586 | 8,661 | 81.8 |
| Karamay |  | 390 |  | 1 | 1 | 100.0 |  | 600 | 600 | 100.0 |
| Hami |  | NA |  | 2 | 2 | 100.0 |  | 1,180 | 1,180 | 100.0 |
| Aksu Prefecture |  | NA |  | 2 | 1 | 50.0 |  | 1,910 | 1,260 | 66.0 |
| Ili Kazak Autonomous Prefecture |  | NA |  | 3 | 3 | 100.0 |  | 2,550 | 2,550 | 100.0 |
| Shihezi |  | NA |  | 2 | 1 | 50.0 |  | 2,160 | 800 | 37.0 |

* Collected from China City Statistical Yearbook 2015.

† Data by December 31, 2017. It should be noted that there were 9 cities whose coverage rates of class 3 hospitals were less than 100% but coverage rates of hospital beds were equal to 100%, because there were 62 (4.2%) hospitals whose numbers of beds were not available and thus excluded from summing the numbers of beds for relevant cities.
