# Supplementary material for: Ambient air pollution and cause-specific risk of hospital admission in China: A nationwide time-series study
Source: PLoS Med. 2020 Aug 6;17(8):e1003188. doi: 10.1371/journal.pmed.1003188 (PMC7410211; doi:10.1371/journal.pmed.1003188)
Supplement: S2 Table — (DOCX) [file pmed.1003188.s016.docx]

# S2 Table. Annual number of hospital admissions and average cost per hospitalization in China.

| Variable | Annual number of admissions | Average cost per hospitalization, ¥ |
| --- | --- | --- |
| Certain infectious and parasitic diseases | 2,406,268 | 5,522.75 |
| Neoplasms | 4,630,568 | 15,429.78 |
| Diseases of the blood and blood-forming organs and certain disorders involving the immune mechanism | 612,197 | 6,749.78 |
| Endocrine, nutritional and metabolic diseases | 2,283,872 | 8,011.76 |
| Mental and behavioural disorders | 451,828 | 6,972.43 |
| Diseases of the nervous system | 2,290,297 | 7,468.72 |
| Diseases of the eye and adnexa | 1,673,281 | 5,949.80 |
| Diseases of the ear and mastoid process | 649,438 | 5,707.09 |
| Diseases of the circulatory system | 11,632,157 | 10,221.93 |
| Diseases of the respiratory system | 10,536,190 | 5,666.32 |
| Diseases of the digestive system | 7,655,511 | 8,180.63 |
| Diseases of the skin and subcutaneous tissue | 611,311 | 5,926.53 |
| Diseases of the musculoskeletal system and connective tissue | 2,573,560 | 10,469.26 |
| Diseases of the genitourinary system | 4,532,674 | 8,163.76 |

Collected from China Health and Family Planning Statistical Yearbook 2017. ¥ = Chinese yuan.
