# Supplementary material for: Ambient air pollution and cause-specific risk of hospital admission in China: A nationwide time-series study
Source: PLoS Med. 2020 Aug 6;17(8):e1003188. doi: 10.1371/journal.pmed.1003188 (PMC7410211; doi:10.1371/journal.pmed.1003188)
Supplement: S3 Table — (DOCX) [file pmed.1003188.s017.docx]

# S3 Table. Demographic characteristics of cause-specific hospital admissions in 252 Chinese cities, 2013-2017.

| Variable | Nationwide | North | South |
| --- | --- | --- | --- |
| Certain infectious and parasitic diseases | | | |
| Total | 4,392,755 | 1,400,885 | 2,991,870 |
| Sex |  |  |  |
| Male (%) | 2,694,622 (61.3) | 838,044 (59.8) | 1,856,578 (62.1) |
| Female (%) | 1,698,133 (38.7) | 562,841 (40.2) | 1,135,292 (37.9) |
| Age, y |  |  |  |
| <65 (%) | 3,826,385 (87.1) | 1,229,680 (87.8) | 2,596,705 (86.8) |
| 65-74 (%) | 333,226 (7.6) | 98,777 (7.1) | 234,449 (7.8) |
| ≥75 (%) | 233,144 (5.3) | 72,428 (5.2) | 160,716 (5.4) |
|  |  |  |  |
| Neoplasms | | | |
| Total | 15,801,214 | 6,206,185 | 9,595,029 |
| Sex |  |  |  |
| Male (%) | 7,096,157 (44.9) | 2,679,350 (43.2) | 4,416,807 (46.0) |
| Female (%) | 8,705,057 (55.1) | 3,526,835 (56.8) | 5,178,222 (54.0) |
| Age, y |  |  |  |
| <65 (%) | 11,839,328 (74.9) | 4,652,214 (75.0) | 7,187,114 (74.9) |
| 65-74 (%) | 2,657,533 (16.8) | 1,049,372 (16.9) | 1,608,161 (16.8) |
| ≥75 (%) | 1,304,353 (8.3) | 504,599 (8.1) | 799,754 (8.3) |
|  |  |  |  |
| Diseases of the blood and blood-forming organs and certain disorders involving the immune mechanism | | | |
| Total | 1,490,481 | 565,252 | 925,229 |
| Sex |  |  |  |
| Male (%) | 738,866 (49.6) | 276,230 (48.9) | 462,636 (50.0) |
| Female (%) | 751,615 (50.4) | 289,022 (51.1) | 462,593 (50.0) |
| Age, y |  |  |  |
| <65 (%) | 1,241,265 (83.3) | 471,843 (83.5) | 769,422 (83.2) |
| 65-74 (%) | 135,646 (9.1) | 50,841 (9.0) | 84,805 (9.2) |
| ≥75 (%) | 113,570 (7.6) | 42,568 (7.5) | 71,002 (7.7) |
|  |  |  |  |
| Endocrine, nutritional and metabolic diseases | | | |
| Total | 4,956,619 | 2,007,744 | 2,948,875 |
| Sex |  |  |  |
| Male (%) | 2,349,899 (47.4) | 978,757 (48.7) | 1,371,142 (46.5) |
| Female (%) | 2,606,720 (52.6) | 1,028,987 (51.3) | 1,577,733 (53.5) |
| Age, y |  |  |  |
| <65 (%) | 3,629,230 (73.2) | 1,506,479 (75.0) | 2,122,751 (72.0) |
| 65-74 (%) | 829,785 (16.7) | 323,253 (16.1) | 506,532 (17.2) |
| ≥75 (%) | 497,604 (10.0) | 178,012 (8.9) | 319,592 (10.8) |
|  |  |  |  |
| Mental and behavioural disorders | | | |
| Total | 1,443,003 | 473,610 | 969,393 |
| Sex |  |  |  |
| Male (%) | 680,447 (47.2) | 209,312 (44.2) | 471,135 (48.6) |
| Female (%) | 762,556 (52.8) | 264,298 (55.8) | 498,258 (51.4) |
| Age, y |  |  |  |
| <65 (%) | 1,219,630 (84.5) | 409,716 (86.5) | 809,914 (83.5) |
| 65-74 (%) | 128,468 (8.9) | 40,190 (8.5) | 88,278 (9.1) |
| ≥75 (%) | 94,905 (6.6) | 23,704 (5.0) | 71,201 (7.3) |
|  |  |  |  |
| Diseases of the nervous system | | | |
| Total | 4,433,661 | 1,902,487 | 2,531,174 |
| Sex |  |  |  |
| Male (%) | 2,354,721 (53.1) | 1,005,007 (52.8) | 1,349,714 (53.3) |
| Female (%) | 2,078,940 (46.9) | 897,480 (47.2) | 1,181,460 (46.7) |
| Age, y |  |  |  |
| <65 (%) | 3,017,454 (68.1) | 1,332,279 (70.0) | 1,685,175 (66.6) |
| 65-74 (%) | 776,333 (17.5) | 325,965 (17.1) | 450,368 (17.8) |
| ≥75 (%) | 639,874 (14.4) | 244,243 (12.8) | 395,631 (15.6) |
|  |  |  |  |
| Diseases of the eye and adnexa | | | |
| Total | 4,457,851 | 1,744,632 | 2,713,219 |
| Sex |  |  |  |
| Male (%) | 2,065,288 (46.3) | 811,270 (46.5) | 1,254,018 (46.2) |
| Female (%) | 2,392,563 (53.7) | 933,362 (53.5) | 1,459,201 (53.8) |
| Age, y |  |  |  |
| <65 (%) | 2,458,002 (55.1) | 993,984 (57.0) | 1,464,018 (54.0) |
| 65-74 (%) | 1,098,286 (24.6) | 412,825 (23.7) | 685,461 (25.3) |
| ≥75 (%) | 901,563 (20.2) | 337,823 (19.4) | 563,740 (20.8) |
|  |  |  |  |
| Diseases of the ear and mastoid process | | | |
| Total | 1,219,916 | 456,841 | 763,075 |
| Sex |  |  |  |
| Male (%) | 552,983 (45.3) | 208,391 (45.6) | 344,592 (45.2) |
| Female (%) | 666,933 (54.7) | 248,450 (54.4) | 418,483 (54.8) |
| Age, y |  |  |  |
| <65 (%) | 980,677 (80.4) | 372,604 (81.6) | 608,073 (79.7) |
| 65-74 (%) | 158,311 (13.0) | 56,600 (12.4) | 101,711 (13.3) |
| ≥75 (%) | 80,928 (6.6) | 27,637 (6.0) | 53,291 (7.0) |
|  |  |  |  |
| Diseases of the circulatory system | | | |
| Total | 20,297,186 | 9,291,866 | 11,005,320 |
| Sex |  |  |  |
| Male (%) | 11,636,063 (57.3) | 5,337,141 (57.4) | 6,298,922 (57.2) |
| Female (%) | 8,661,123 (42.7) | 3,954,725 (42.6) | 4,706,398 (42.8) |
| Age, y |  |  |  |
| <65 (%) | 10,344,670 (51.0) | 5,052,620 (54.4) | 5,292,050 (48.1) |
| 65-74 (%) | 5,027,650 (24.8) | 2,276,806 (24.5) | 2,750,844 (25.0) |
| ≥75 (%) | 4,924,866 (24.3) | 1,962,440 (21.1) | 2,962,426 (26.9) |
|  |  |  |  |
| Diseases of the respiratory system | | | |
| Total | 14,993,789 | 5,241,592 | 9,752,197 |
| Sex |  |  |  |
| Male (%) | 9,097,347 (60.7) | 3,130,424 (59.7) | 5,966,923 (61.2) |
| Female (%) | 5,896,442 (39.3) | 2,111,168 (40.3) | 3,785,274 (38.8) |
| Age, y |  |  |  |
| <65 (%) | 11,156,312 (74.4) | 3,930,921 (75.0) | 7,225,391 (74.1) |
| 65-74 (%) | 1,602,558 (10.7) | 536,007 (10.2) | 1,066,551 (10.9) |
| ≥75 (%) | 2,234,919 (14.9) | 774,664 (14.8) | 1,460,255 (15.0) |
|  |  |  |  |
| Diseases of the digestive system | | | |
| Total | 13,531,339 | 4,653,348 | 8,877,991 |
| Sex |  |  |  |
| Male (%) | 7,755,483 (57.3) | 2,652,147 (57.0) | 5,103,336 (57.5) |
| Female (%) | 5,775,856 (42.7) | 2,001,201 (43.0) | 3,774,655 (42.5) |
| Age, y |  |  |  |
| <65 (%) | 10,076,517 (74.5) | 3,445,336 (74.0) | 6,631,181 (74.7) |
| 65-74 (%) | 1,986,199 (14.7) | 686,905 (14.8) | 1,299,294 (14.6) |
| ≥75 (%) | 1,468,623 (10.9) | 521,107 (11.2) | 947,516 (10.7) |
|  |  |  |  |
| Diseases of the skin and subcutaneous tissue | | | |
| Total | 1,308,892 | 432,235 | 876,657 |
| Sex |  |  |  |
| Male (%) | 732,876 (56.0) | 237,334 (54.9) | 495,542 (56.5) |
| Female (%) | 576,016 (44.0) | 194,901 (45.1) | 381,115 (43.5) |
| Age, y |  |  |  |
| <65 (%) | 1,067,230 (81.5) | 359,549 (83.2) | 707,681 (80.7) |
| 65-74 (%) | 130,861 (10.0) | 39,316 (9.1) | 91,545 (10.4) |
| ≥75 (%) | 110,801 (8.5) | 33,370 (7.7) | 77,431 (8.8) |
|  |  |  |  |
| Diseases of the musculoskeletal system and connective tissue | | | |
| Total | 5,577,996 | 1,883,754 | 3,694,242 |
| Sex |  |  |  |
| Male (%) | 2,315,131 (41.5) | 770,298 (40.9) | 1,544,833 (41.8) |
| Female (%) | 3,262,865 (58.5) | 1,113,456 (59.1) | 2,149,409 (58.2) |
| Age, y |  |  |  |
| <65 (%) | 4,096,183 (73.4) | 1,436,548 (76.3) | 2,659,635 (72.0) |
| 65-74 (%) | 908,860 (16.3) | 294,779 (15.6) | 614,081 (16.6) |
| ≥75 (%) | 572,953 (10.3) | 152,427 (8.1) | 420,526 (11.4) |
|  |  |  |  |
| Diseases of the genitourinary system | | | |
| Total | 9,325,491 | 3,115,778 | 6,209,713 |
| Sex |  |  |  |
| Male (%) | 3,964,260 (42.5) | 1,283,287 (41.2) | 2,680,973 (43.2) |
| Female (%) | 5,361,231 (57.5) | 1,832,491 (58.8) | 3,528,740 (56.8) |
| Age, y |  |  |  |
| <65 (%) | 7,555,921 (81.0) | 2,522,203 (80.9) | 5,033,718 (81.1) |
| 65-74 (%) | 1,047,311 (11.2) | 346,952 (11.1) | 700,359 (11.3) |
| ≥75 (%) | 722,259 (7.7) | 246,623 (7.9) | 475,636 (7.7) |
|  |  |  |  |
| Other ICD-10 codes related to the above diseases* | | | |
| Total | 14,108,674 | 5,657,723 | 8,450,951 |
| Sex |  |  |  |
| Male (%) | 6,591,719 (46.7) | 2,506,155 (44.3) | 4,085,564 (48.3) |
| Female (%) | 7,516,955 (53.3) | 3,151,568 (55.7) | 4,365,387 (51.7) |
| Age, y |  |  |  |
| <65 (%) | 11,146,505 (79.0) | 4,447,735 (78.6) | 6,698,770 (79.3) |
| ≥65 (%) | 2,962,169 (21.0) | 1,209,988 (21.4) | 1,752,181 (20.7) |

The hospital admissions are grouped into 14 major disease categories by primary discharge diagnosis codes, based on the chapter division of the ICD-10 diagnostic coding system. The two regions of China (North and South) are divided by the Huai River-Qinling Mountain line.

* There are some ICD-10 codes covered by the 188 minor disease categories (based on the Clinical Classifications Software [CCS]) but not by the 14 major disease categories, and vice versa. This is because the two levels of disease categories form an approximate but not strict hierarchical structure. For example, ICD-10 codes R16.0 and R16.2 (“hepatomegaly [with splenomegaly], not elsewhere classified”) were not used in the analyses for major disease categories, since the ICD-10 chapter “symptoms, signs, and abnormal clinical and laboratory findings, not elsewhere classified” was not included in the scope of this study; however, these codes were used in the analyses for minor disease categories, since the CCS category “other liver diseases” was included in the scope of this study. In fact, CCS has its own hierarchy, where the first-level classification corresponds to the chapter division of the ICD-9 diagnostic coding system. However, since ICD-10 is an updated version of ICD-9 (and is arguably more advanced), we decided to define our major disease categories based on the chapter division of the ICD-10 diagnostic coding system.
