# Supplementary material for: Ambient air pollution and cause-specific risk of hospital admission in China: A nationwide time-series study
Source: PLoS Med. 2020 Aug 6;17(8):e1003188. doi: 10.1371/journal.pmed.1003188 (PMC7410211; doi:10.1371/journal.pmed.1003188)
Supplement: S4 Table — (DOCX) [file pmed.1003188.s018.docx]

# S4 Table. Summary statistics of annual-average levels of ambient air pollutants and weather conditions in 252 Chinese cities, 2013-2017.

|  |  |  | Percentile | | |  |
| --- | --- | --- | --- | --- | --- | --- |
| Variable | Mean±SD | Minimum | 25th | 50th | 75th | Maximum |
| PM_2.5_, μg/m^3^ | 50.6±18.2 | 14.9 | 37.0 | 50.1 | 61.3 | 104.9 |
| Cool season | 64.1±24.3 | 16.5 | 45.2 | 63.7 | 79.2 | 140.4 |
| Warm season | 37.1±13.0 | 11.8 | 27.4 | 36.6 | 44.8 | 76.2 |
| North region | 58.7±20.1 | 15.9 | 42.5 | 59.3 | 73.3 | 104.9 |
| South region | 44.6±14.0 | 14.9 | 32.0 | 43.7 | 55.7 | 72.1 |
| O_3_, μg/m^3^ | 87.2±11.1 | 60.5 | 80.3 | 88.3 | 95.8 | 112.8 |
| Cool season | 68.7±11.8 | 35.2 | 60.4 | 68.7 | 76.2 | 100.3 |
| Warm season | 105.4±20.1 | 58.1 | 88.8 | 106.9 | 120.5 | 155.8 |
| North region | 90.2±11.5 | 62.8 | 82.6 | 90.7 | 98.2 | 112.8 |
| South region | 85.0±10.3 | 60.5 | 77.7 | 84.8 | 92.6 | 106.9 |
| SO_2_, μg/m^3^ | 24.0±14.0 | 2.8 | 14.5 | 20.1 | 27.8 | 78.9 |
| Cool season | 30.8±20.9 | 2.8 | 16.4 | 24.3 | 37.6 | 121.0 |
| Warm season | 17.3±8.4 | 2.7 | 11.2 | 15.5 | 21.7 | 57.8 |
| North region | 33.3±16.0 | 7.2 | 21.4 | 28.2 | 42.7 | 78.9 |
| South region | 17.2±6.3 | 2.8 | 12.3 | 16.7 | 21.9 | 37.2 |
| NO_2_, μg/m^3^ | 31.4±10.7 | 10.2 | 22.7 | 31.3 | 38.3 | 60.7 |
| Cool season | 37.5±13.1 | 11.8 | 26.8 | 37.1 | 45.9 | 73.0 |
| Warm season | 25.3±8.8 | 8.4 | 18.2 | 25.0 | 31.2 | 52.7 |
| North region | 35.8±10.9 | 13.5 | 27.4 | 36.1 | 43.4 | 60.7 |
| South region | 28.1±9.4 | 10.2 | 20.7 | 27.7 | 34.4 | 50.5 |
| CO, mg/m^3^ | 1.1±0.3 | 0.5 | 0.8 | 1.0 | 1.2 | 2.4 |
| Cool season | 1.3±0.4 | 0.5 | 1.0 | 1.1 | 1.5 | 2.9 |
| Warm season | 0.9±0.3 | 0.4 | 0.7 | 0.9 | 1.0 | 1.9 |
| North region | 1.2±0.4 | 0.5 | 0.9 | 1.2 | 1.5 | 2.4 |
| South region | 0.9±0.2 | 0.5 | 0.8 | 0.9 | 1.0 | 1.6 |
| Temperature, °C | 14.7±5.4 | -1.6 | 11.3 | 15.8 | 18.1 | 24.9 |
| Cool season | 7.4±7.6 | -15.9 | 2.6 | 8.7 | 12.1 | 21.6 |
| Warm season | 22.0±3.5 | 12.0 | 19.6 | 23.0 | 24.3 | 28.0 |
| North region | 10.2±4.4 | -1.6 | 6.8 | 10.1 | 13.9 | 23.0 |
| South region | 18.0±3.2 | 6.6 | 16.5 | 17.7 | 19.8 | 24.9 |
| Relative humidity, % | 69.2±10.3 | 33.2 | 61.7 | 72.4 | 77.3 | 90.8 |
| Cool season | 67.4±10.5 | 32.9 | 59.3 | 70.4 | 75.8 | 88.5 |
| Warm season | 70.9±10.8 | 32.6 | 65.2 | 74.0 | 78.2 | 93.0 |
| North region | 60.5±8.8 | 33.2 | 56.4 | 61.6 | 66.8 | 79.0 |
| South region | 75.5±5.6 | 52.6 | 73.6 | 76.9 | 78.8 | 90.8 |

The cool season is from October to next March; the warm season is from April to September. The two regions of China (North and South) are divided by the Huai River-Qinling Mountain line.
