# Supplementary material for: Ambient air pollution and cause-specific risk of hospital admission in China: A nationwide time-series study
Source: PLoS Med. 2020 Aug 6;17(8):e1003188. doi: 10.1371/journal.pmed.1003188 (PMC7410211; doi:10.1371/journal.pmed.1003188)
Supplement: S5 Table — (DOCX) [file pmed.1003188.s019.docx]

# S5 Table. Pearson correlation coefficients among daily levels of ambient air pollutants and weather conditions, on average across all cities.

| Variable | PM_2.5_ | O_3_ | SO_2_ | NO_2_ | CO | Temperature | Relative humidity |
| --- | --- | --- | --- | --- | --- | --- | --- |
| PM_2.5_ | 1.00 | -0.04 | 0.54 | 0.63 | 0.63 | -0.38 | -0.11 |
| O_3_ |  | 1.00 | -0.09 | -0.15 | -0.22 | 0.49 | -0.31 |
| SO_2_ |  |  | 1.00 | 0.56 | 0.48 | -0.36 | -0.22 |
| NO_2_ |  |  |  | 1.00 | 0.57 | -0.41 | -0.12 |
| CO |  |  |  |  | 1.00 | -0.39 | 0.04 |
| Temperature |  |  |  |  |  | 1.00 | 0.10 |
| Relative humidity |  |  |  |  |  |  | 1.00 |
