# Supplementary material for: Ambient air pollution and cause-specific risk of hospital admission in China: A nationwide time-series study
Source: PLoS Med. 2020 Aug 6;17(8):e1003188. doi: 10.1371/journal.pmed.1003188 (PMC7410211; doi:10.1371/journal.pmed.1003188)
Supplement: S19 Table — (DOCX) [file pmed.1003188.s033.docx]

# S19 Table. Percent change in hospital admissions per 10-μg/m^3^ increase in O_3_ (lag 0-3 days) by minor disease categories, on average across all cities.

|  |  |  |  |  | Percent change in admissions per  10-μg/m^3^ increase in O_3_, % | | | | | | |
| --- | --- | --- | --- | --- | --- | --- | --- | --- | --- | --- | --- |
|  |  |  |  |  | Single-pollutant model | | |  | Two-pollutant model | | |
| CCS code | Disease category |  | Total no. of admissions |  | Point estimate  (95% CI) | Unadjusted  *P* value | Adjusted  *P* value |  | Point estimate  (95% CI) | Unadjusted  *P* value | Adjusted  *P* value |
| 1 | Tuberculosis |  | 911,001 |  | -0.11 (-0.35, 0.12) | 0.348 | 0.782 |  | -0.14 (-0.39, 0.10) | 0.250 | 0.819 |
| 2 | Septicemia (except in labor) |  | 247,832 |  | 0.29 (-0.10, 0.68) | 0.147 | 0.701 |  | 0.16 (-0.26, 0.57) | 0.465 | 0.917 |
| 3 | Bacterial infection; unspecified site |  | 108,626 |  | 0.40 (-0.24, 1.05) | 0.218 | 0.701 |  | 0.30 (-0.38, 0.98) | 0.388 | 0.869 |
| 4 | Mycoses |  | 102,580 |  | 0.17 (-0.42, 0.76) | 0.573 | 0.844 |  | 0.04 (-0.59, 0.66) | 0.906 | 0.979 |
| 5 | HIV infection |  | 69,110 |  | 0.34 (-0.37, 1.05) | 0.343 | 0.782 |  | 0.47 (-0.29, 1.23) | 0.228 | 0.819 |
| 6 | Hepatitis |  | 812,827 |  | 0.15 (-0.11, 0.41) | 0.269 | 0.779 |  | 0.04 (-0.21, 0.29) | 0.735 | 0.947 |
| 7 | Viral infection |  | 1,287,445 |  | -0.07 (-0.30, 0.16) | 0.543 | 0.844 |  | 0.03 (-0.23, 0.29) | 0.805 | 0.968 |
| 8 | Other infections; including parasitic |  | 191,575 |  | 0.09 (-0.35, 0.52) | 0.696 | 0.882 |  | 0.12 (-0.35, 0.58) | 0.628 | 0.947 |
| 9 | Sexually transmitted infections (not HIV or hepatitis) |  | 88,870 |  | -0.06 (-0.94, 0.83) | 0.902 | 0.969 |  | -0.21 (-1.15, 0.73) | 0.659 | 0.947 |
| 10 | Immunizations and screening for infectious disease |  | 9,495 |  | 0.15 (-1.84, 2.14) | 0.880 | 0.962 |  | -0.51 (-2.61, 1.59) | 0.635 | 0.947 |
| 11 | Cancer of head and neck |  | 502,628 |  | -0.20 (-0.51, 0.12) | 0.220 | 0.701 |  | -0.18 (-0.51, 0.16) | 0.299 | 0.819 |
| 12 | Cancer of esophagus |  | 510,443 |  | -0.11 (-0.40, 0.17) | 0.438 | 0.796 |  | -0.15 (-0.45, 0.15) | 0.333 | 0.819 |
| 13 | Cancer of stomach |  | 854,446 |  | 0.10 (-0.13, 0.33) | 0.377 | 0.782 |  | 0.06 (-0.18, 0.30) | 0.612 | 0.947 |
| 14 | Cancer of colon |  | 495,169 |  | 0.11 (-0.21, 0.43) | 0.502 | 0.836 |  | 0.09 (-0.24, 0.43) | 0.586 | 0.947 |
| 15 | Cancer of rectum and anus |  | 505,433 |  | 0.02 (-0.27, 0.31) | 0.894 | 0.969 |  | 0.01 (-0.30, 0.31) | 0.965 | 0.979 |
| 16 | Cancer of liver and intrahepatic bile duct |  | 779,960 |  | -0.04 (-0.27, 0.19) | 0.733 | 0.904 |  | -0.08 (-0.32, 0.16) | 0.512 | 0.930 |
| 17 | Cancer of pancreas |  | 191,736 |  | 0.19 (-0.26, 0.64) | 0.404 | 0.794 |  | 0.05 (-0.42, 0.53) | 0.830 | 0.968 |
| 18 | Cancer of other GI organs; peritoneum |  | 227,609 |  | -0.12 (-0.54, 0.29) | 0.563 | 0.844 |  | -0.12 (-0.56, 0.32) | 0.588 | 0.947 |
| 19 | Cancer of bronchus; lung |  | 1,872,596 |  | 0.17 (0.00, 0.34) | 0.045* | 0.427 |  | 0.17 (-0.02, 0.36) | 0.078 | 0.569 |
| 20 | Cancer; other respiratory and intrathoracic |  | 14,943 |  | 1.72 (0.10, 3.35) | 0.037* | 0.405 |  | 1.53 (-0.18, 3.24) | 0.079 | 0.569 |
| 21 | Cancer of bone and connective tissue |  | 94,158 |  | -0.14 (-0.79, 0.50) | 0.666 | 0.869 |  | -0.31 (-0.99, 0.37) | 0.368 | 0.834 |
| 22 | Melanomas of skin |  | 24,963 |  | -0.27 (-1.54, 1.01) | 0.683 | 0.873 |  | -0.10 (-1.45, 1.25) | 0.886 | 0.979 |
| 23 | Other non-epithelial cancer of skin |  | 77,862 |  | 0.44 (-0.37, 1.24) | 0.284 | 0.782 |  | 0.49 (-0.34, 1.32) | 0.245 | 0.819 |
| 24 | Cancer of breast |  | 1,031,596 |  | 0.07 (-0.17, 0.31) | 0.581 | 0.844 |  | 0.05 (-0.19, 0.28) | 0.702 | 0.947 |
| 25 | Cancer of uterus |  | 173,071 |  | -0.30 (-0.77, 0.18) | 0.227 | 0.710 |  | -0.28 (-0.79, 0.22) | 0.276 | 0.819 |
| 26 | Cancer of cervix |  | 600,054 |  | 0.22 (-0.08, 0.53) | 0.156 | 0.701 |  | 0.22 (-0.07, 0.51) | 0.143 | 0.759 |
| 27 | Cancer of ovary |  | 202,125 |  | -0.14 (-0.59, 0.31) | 0.552 | 0.844 |  | -0.30 (-0.78, 0.17) | 0.213 | 0.819 |
| 28 | Cancer of other female genital organs |  | 38,206 |  | 0.46 (-0.56, 1.47) | 0.376 | 0.782 |  | 0.53 (-0.55, 1.60) | 0.339 | 0.819 |
| 29 | Cancer of prostate |  | 204,959 |  | -0.04 (-0.47, 0.39) | 0.842 | 0.957 |  | -0.06 (-0.52, 0.39) | 0.786 | 0.967 |
| 30 | Cancer of testis |  | 12,452 |  | -0.65 (-2.44, 1.14) | 0.479 | 0.811 |  | -0.72 (-2.63, 1.18) | 0.457 | 0.913 |
| 31 | Cancer of other male genital organs |  | 18,617 |  | -0.55 (-2.06, 0.96) | 0.473 | 0.811 |  | -0.58 (-2.18, 1.02) | 0.480 | 0.926 |
| 32 | Cancer of bladder |  | 212,065 |  | 0.14 (-0.31, 0.59) | 0.529 | 0.844 |  | 0.13 (-0.38, 0.64) | 0.612 | 0.947 |
| 33 | Cancer of kidney and renal pelvis |  | 165,670 |  | -0.15 (-0.64, 0.34) | 0.548 | 0.844 |  | -0.12 (-0.64, 0.39) | 0.638 | 0.947 |
| 34 | Cancer of other urinary organs |  | 26,281 |  | 0.12 (-1.12, 1.36) | 0.850 | 0.957 |  | 0.04 (-1.26, 1.33) | 0.957 | 0.979 |
| 35 | Cancer of brain and nervous system |  | 137,704 |  | 0.17 (-0.38, 0.71) | 0.546 | 0.844 |  | 0.11 (-0.47, 0.68) | 0.714 | 0.947 |
| 36 | Cancer of thyroid |  | 563,513 |  | 0.08 (-0.22, 0.37) | 0.608 | 0.844 |  | 0.12 (-0.19, 0.43) | 0.443 | 0.895 |
| 37 | Hodgkin’s disease |  | 34,210 |  | -0.00 (-1.07, 1.06) | 0.995 | 1.000 |  | -0.25 (-1.37, 0.87) | 0.658 | 0.947 |
| 38 | Non-Hodgkin’s lymphoma |  | 356,137 |  | 0.05 (-0.32, 0.41) | 0.802 | 0.942 |  | -0.04 (-0.40, 0.32) | 0.845 | 0.969 |
| 39 | Leukemias |  | 645,532 |  | 0.06 (-0.21, 0.33) | 0.658 | 0.869 |  | 0.05 (-0.22, 0.33) | 0.695 | 0.947 |
| 40 | Multiple myeloma |  | 146,549 |  | 0.40 (-0.17, 0.96) | 0.168 | 0.701 |  | 0.41 (-0.18, 0.99) | 0.175 | 0.819 |
| 41 | Cancer; other and unspecified primary |  | 129,460 |  | 0.39 (-0.16, 0.94) | 0.161 | 0.701 |  | 0.34 (-0.23, 0.92) | 0.244 | 0.819 |
| 42 | Secondary malignancies |  | 486,077 |  | 0.13 (-0.16, 0.43) | 0.378 | 0.782 |  | 0.07 (-0.27, 0.41) | 0.689 | 0.947 |
| 43 | Malignant neoplasm without specification of site |  | 61,211 |  | -0.36 (-1.14, 0.42) | 0.369 | 0.782 |  | -0.01 (-0.84, 0.81) | 0.979 | 0.984 |
| 44 | Neoplasms of unspecified nature or uncertain behavior |  | 508,677 |  | 0.10 (-0.20, 0.39) | 0.522 | 0.844 |  | 0.01 (-0.28, 0.31) | 0.926 | 0.979 |
| 45 | Maintenance chemotherapy; radiotherapy |  | 12,173,649 |  | -0.07 (-0.18, 0.05) | 0.241 | 0.720 |  | -0.07 (-0.20, 0.06) | 0.292 | 0.819 |
| 46 | Benign neoplasm of uterus |  | 1,010,719 |  | -0.00 (-0.26, 0.26) | 1.000 | 1.000 |  | -0.05 (-0.31, 0.22) | 0.735 | 0.947 |
| 47 | Other and unspecified benign neoplasm |  | 2,884,383 |  | -0.11 (-0.29, 0.07) | 0.217 | 0.701 |  | -0.11 (-0.30, 0.08) | 0.253 | 0.819 |
| 48 | Thyroid disorders |  | 1,080,281 |  | -0.12 (-0.33, 0.08) | 0.241 | 0.720 |  | -0.14 (-0.36, 0.08) | 0.219 | 0.819 |
| 49 | Diabetes mellitus without complication |  | 1,559,966 |  | 0.03 (-0.15, 0.21) | 0.741 | 0.904 |  | -0.06 (-0.25, 0.14) | 0.579 | 0.947 |
| 50 | Diabetes mellitus with complications |  | 1,601,337 |  | 0.14 (-0.06, 0.35) | 0.172 | 0.701 |  | -0.04 (-0.26, 0.18) | 0.727 | 0.947 |
| 51 | Other endocrine disorders |  | 391,190 |  | -0.05 (-0.45, 0.34) | 0.796 | 0.941 |  | -0.10 (-0.51, 0.31) | 0.635 | 0.947 |
| 52 | Nutritional deficiencies |  | 35,728 |  | -0.84 (-2.03, 0.34) | 0.163 | 0.701 |  | -0.86 (-2.19, 0.47) | 0.204 | 0.819 |
| 53 | Disorders of lipid metabolism |  | 68,063 |  | -0.27 (-1.19, 0.65) | 0.561 | 0.844 |  | -0.45 (-1.39, 0.49) | 0.348 | 0.819 |
| 54 | Gout and other crystal arthropathies |  | 189,684 |  | -0.13 (-0.55, 0.29) | 0.536 | 0.844 |  | -0.26 (-0.71, 0.20) | 0.270 | 0.819 |
| 55 | Fluid and electrolyte disorders |  | 78,150 |  | -0.29 (-0.96, 0.37) | 0.391 | 0.783 |  | -0.33 (-1.04, 0.38) | 0.359 | 0.831 |
| 57 | Immunity disorders |  | 12,701 |  | -0.72 (-2.33, 0.90) | 0.384 | 0.783 |  | -0.57 (-2.29, 1.15) | 0.517 | 0.930 |
| 58 | Other nutritional; endocrine; and metabolic disorders |  | 229,833 |  | -0.26 (-0.67, 0.15) | 0.213 | 0.701 |  | -0.44 (-0.88, 0.00) | 0.052 | 0.513 |
| 59 | Deficiency and other anemia |  | 682,025 |  | 0.28 (0.01, 0.55) | 0.041* | 0.405 |  | 0.23 (-0.06, 0.51) | 0.115 | 0.719 |
| 60 | Acute posthemorrhagic anemia |  | 2,401 |  | 0.09 (-12.28, 12.45) | 0.989 | 1.000 |  | -1.67 (-15.61, 12.28) | 0.815 | 0.968 |
| 62 | Coagulation and hemorrhagic disorders |  | 592,711 |  | -0.30 (-0.58, -0.03) | 0.032* | 0.405 |  | -0.29 (-0.58, 0.00) | 0.050 | 0.513 |
| 63 | Diseases of white blood cells |  | 70,776 |  | -0.06 (-0.79, 0.66) | 0.867 | 0.957 |  | 0.08 (-0.71, 0.87) | 0.839 | 0.968 |
| 64 | Other hematologic conditions |  | 78,999 |  | -0.57 (-1.25, 0.10) | 0.096 | 0.602 |  | -0.66 (-1.38, 0.06) | 0.074 | 0.569 |
| 65 | Mental retardation |  | 30,747 |  | 0.11 (-0.99, 1.22) | 0.845 | 0.957 |  | 0.78 (-0.47, 2.04) | 0.221 | 0.819 |
| 66 | Alcohol-related mental disorders |  | 63,812 |  | -0.34 (-1.19, 0.51) | 0.432 | 0.796 |  | -0.45 (-1.35, 0.46) | 0.334 | 0.819 |
| 67 | Substance-related mental disorders |  | 9,443 |  | -0.19 (-2.35, 1.98) | 0.866 | 0.957 |  | -0.14 (-2.43, 2.14) | 0.901 | 0.979 |
| 68 | Senility and organic mental disorders |  | 197,581 |  | 0.17 (-0.28, 0.62) | 0.464 | 0.811 |  | 0.13 (-0.35, 0.61) | 0.603 | 0.947 |
| 69 | Affective disorders |  | 310,099 |  | -0.18 (-0.55, 0.19) | 0.332 | 0.782 |  | -0.27 (-0.68, 0.14) | 0.197 | 0.819 |
| 70 | Schizophrenia and related disorders |  | 348,859 |  | -0.22 (-0.65, 0.21) | 0.317 | 0.782 |  | -0.17 (-0.64, 0.29) | 0.468 | 0.917 |
| 71 | Other psychoses |  | 15,470 |  | 1.74 (0.08, 3.40) | 0.040* | 0.405 |  | 1.67 (-0.17, 3.51) | 0.075 | 0.569 |
| 72 | Anxiety; somatoform; dissociative; and personality disorders |  | 441,926 |  | 0.08 (-0.24, 0.41) | 0.606 | 0.844 |  | 0.06 (-0.29, 0.42) | 0.720 | 0.947 |
| 73 | Preadult disorders |  | 12,140 |  | -1.14 (-2.82, 0.55) | 0.187 | 0.701 |  | -1.24 (-3.49, 1.01) | 0.280 | 0.819 |
| 74 | Other mental conditions |  | 56,465 |  | 0.25 (-0.75, 1.26) | 0.624 | 0.853 |  | 0.08 (-1.03, 1.18) | 0.894 | 0.979 |
| 76 | Meningitis (except that caused by tuberculosis or sexually transmitted disease) |  | 76,103 |  | -0.12 (-0.81, 0.57) | 0.739 | 0.904 |  | -0.19 (-0.93, 0.55) | 0.615 | 0.947 |
| 77 | Encephalitis (except that caused by tuberculosis or sexually transmitted disease) |  | 366,528 |  | -0.18 (-0.56, 0.19) | 0.331 | 0.782 |  | -0.10 (-0.48, 0.29) | 0.622 | 0.947 |
| 78 | Other CNS infection and poliomyelitis |  | 75,956 |  | -0.03 (-0.81, 0.75) | 0.947 | 0.989 |  | -0.02 (-0.80, 0.77) | 0.969 | 0.979 |
| 79 | Parkinson’s disease |  | 158,499 |  | 0.00 (-0.55, 0.56) | 0.995 | 1.000 |  | -0.08 (-0.67, 0.51) | 0.792 | 0.967 |
| 80 | Multiple sclerosis |  | 19,147 |  | -0.23 (-1.69, 1.23) | 0.760 | 0.922 |  | 0.54 (-1.02, 2.10) | 0.500 | 0.929 |
| 81 | Other hereditary and degenerative nervous system conditions |  | 241,382 |  | -0.02 (-0.45, 0.40) | 0.917 | 0.974 |  | -0.05 (-0.51, 0.40) | 0.814 | 0.968 |
| 82 | Paralysis |  | 188,113 |  | -0.29 (-0.92, 0.33) | 0.356 | 0.782 |  | -0.51 (-1.14, 0.12) | 0.115 | 0.719 |
| 83 | Epilepsy; convulsions |  | 707,298 |  | -0.19 (-0.43, 0.05) | 0.113 | 0.685 |  | -0.31 (-0.58, -0.04) | 0.026* | 0.374 |
| 84 | Headache; including migraine |  | 313,017 |  | -0.05 (-0.44, 0.33) | 0.782 | 0.937 |  | -0.07 (-0.47, 0.34) | 0.737 | 0.947 |
| 85 | Coma; stupor; and brain damage |  | 83,333 |  | -0.19 (-0.93, 0.55) | 0.611 | 0.844 |  | -0.17 (-0.95, 0.61) | 0.666 | 0.947 |
| 86 | Cataract |  | 2,083,086 |  | 0.02 (-0.20, 0.24) | 0.858 | 0.957 |  | 0.93 (-0.09, 1.95) | 0.075 | 0.569 |
| 87 | Retinal detachments; defects; vascular occlusion; and retinopathy |  | 563,655 |  | 0.16 (-0.25, 0.56) | 0.445 | 0.796 |  | 0.13 (-0.28, 0.55) | 0.523 | 0.930 |
| 88 | Glaucoma |  | 357,287 |  | -0.17 (-0.54, 0.20) | 0.375 | 0.782 |  | -0.20 (-0.58, 0.18) | 0.304 | 0.819 |
| 89 | Blindness and vision defects |  | 18,362 |  | -0.85 (-2.42, 0.72) | 0.287 | 0.782 |  | -1.02 (-2.71, 0.68) | 0.241 | 0.819 |
| 90 | Inflammation; infection of eye (except that caused by tuberculosis or sexually transmitteddisease) |  | 297,381 |  | 0.19 (-0.23, 0.62) | 0.378 | 0.782 |  | 0.23 (-0.23, 0.69) | 0.324 | 0.819 |
| 91 | Other eye disorders |  | 1,155,446 |  | -0.23 (-0.48, 0.02) | 0.072 | 0.540 |  | -0.25 (-0.53, 0.03) | 0.076 | 0.569 |
| 92 | Otitis media and related conditions |  | 264,907 |  | -0.13 (-0.54, 0.29) | 0.549 | 0.844 |  | -0.17 (-0.59, 0.25) | 0.425 | 0.888 |
| 93 | Conditions associated with dizziness or vertigo |  | 576,577 |  | 0.05 (-0.22, 0.32) | 0.715 | 0.890 |  | -0.03 (-0.32, 0.26) | 0.835 | 0.968 |
| 94 | Other ear and sense organ disorders |  | 480,097 |  | -0.23 (-0.52, 0.06) | 0.127 | 0.701 |  | -0.24 (-0.55, 0.07) | 0.135 | 0.759 |
| 95 | Other nervous system disorders |  | 1,218,357 |  | 0.04 (-0.17, 0.25) | 0.703 | 0.882 |  | 0.02 (-0.19, 0.24) | 0.822 | 0.968 |
| 96 | Heart valve disorders |  | 361,550 |  | -0.14 (-0.47, 0.19) | 0.414 | 0.794 |  | -0.19 (-0.55, 0.16) | 0.287 | 0.819 |
| 97 | Peri-; endo-; and myocarditis; cardiomyopathy (except that caused by tuberculosis or sexually transmitted disease) |  | 539,506 |  | 0.22 (-0.08, 0.53) | 0.152 | 0.701 |  | 0.16 (-0.14, 0.47) | 0.298 | 0.819 |
| 98 | Essential hypertension |  | 1,932,066 |  | -0.24 (-0.44, -0.04) | 0.017* | 0.295 |  | -0.34 (-0.55, -0.14) | 0.001* | 0.036* |
| 99 | Hypertension with complications and secondary hypertension |  | 224,487 |  | -0.16 (-0.58, 0.27) | 0.476 | 0.811 |  | -0.24 (-0.69, 0.22) | 0.312 | 0.819 |
| 100 | Acute myocardial infarction |  | 905,773 |  | 0.17 (-0.05, 0.39) | 0.124 | 0.701 |  | 0.09 (-0.14, 0.33) | 0.441 | 0.895 |
| 101 | Coronary atherosclerosis and other heart disease |  | 5,602,675 |  | 0.16 (0.05, 0.28) | 0.005* | 0.104 |  | 0.07 (-0.05, 0.19) | 0.245 | 0.819 |
| 102 | Nonspecific chest pain |  | 33,587 |  | -1.00 (-2.09, 0.09) | 0.071 | 0.540 |  | -1.22 (-2.44, -0.01) | 0.049* | 0.513 |
| 103 | Pulmonary heart disease |  | 211,172 |  | 0.35 (-0.17, 0.86) | 0.184 | 0.701 |  | 0.07 (-0.44, 0.57) | 0.791 | 0.967 |
| 104 | Other and ill-defined heart disease |  | 38,582 |  | 0.29 (-0.80, 1.39) | 0.598 | 0.844 |  | 0.18 (-0.98, 1.35) | 0.756 | 0.947 |
| 105 | Conduction disorders |  | 127,505 |  | 0.05 (-0.53, 0.63) | 0.870 | 0.957 |  | 0.16 (-0.45, 0.76) | 0.611 | 0.947 |
| 106 | Cardiac dysrhythmias |  | 1,233,373 |  | 0.10 (-0.09, 0.29) | 0.290 | 0.782 |  | 0.08 (-0.12, 0.28) | 0.421 | 0.888 |
| 107 | Cardiac arrest and ventricular fibrillation |  | 36,199 |  | 0.40 (-0.65, 1.44) | 0.456 | 0.810 |  | 0.39 (-0.72, 1.50) | 0.493 | 0.926 |
| 108 | Congestive heart failure; nonhypertensive |  | 429,258 |  | 0.38 (0.06, 0.71) | 0.021* | 0.333 |  | 0.20 (-0.14, 0.55) | 0.244 | 0.819 |
| 109 | Acute cerebrovascular disease |  | 5,301,927 |  | -0.06 (-0.17, 0.06) | 0.328 | 0.782 |  | -0.12 (-0.24, 0.00) | 0.051 | 0.513 |
| 110 | Occlusion or stenosis of precerebral arteries |  | 87,918 |  | -0.19 (-1.01, 0.64) | 0.660 | 0.869 |  | -0.02 (-0.87, 0.83) | 0.962 | 0.979 |
| 111 | Other and ill-defined cerebrovascular disease |  | 922,625 |  | -0.07 (-0.31, 0.17) | 0.565 | 0.844 |  | -0.14 (-0.40, 0.12) | 0.301 | 0.819 |
| 112 | Transient cerebral ischemia |  | 1,632,182 |  | 0.07 (-0.10, 0.25) | 0.413 | 0.794 |  | 0.01 (-0.18, 0.20) | 0.901 | 0.979 |
| 113 | Late effects of cerebrovascular disease |  | 393,340 |  | 0.22 (-0.10, 0.55) | 0.181 | 0.701 |  | 0.09 (-0.26, 0.43) | 0.624 | 0.947 |
| 114 | Peripheral and visceral atherosclerosis |  | 228,819 |  | 0.17 (-0.26, 0.59) | 0.443 | 0.796 |  | 0.16 (-0.29, 0.61) | 0.492 | 0.926 |
| 115 | Aortic; peripheral; and visceral artery aneurysms |  | 199,457 |  | 0.09 (-0.35, 0.54) | 0.681 | 0.873 |  | 0.03 (-0.45, 0.50) | 0.909 | 0.979 |
| 116 | Aortic and peripheral arterial embolism or thrombosis |  | 49,080 |  | -0.43 (-1.36, 0.50) | 0.365 | 0.782 |  | -0.48 (-1.47, 0.52) | 0.345 | 0.819 |
| 117 | Other circulatory disease |  | 210,983 |  | 0.38 (-0.05, 0.80) | 0.080 | 0.556 |  | 0.38 (-0.06, 0.83) | 0.092 | 0.617 |
| 118 | Phlebitis; thrombophlebitis and thromboembolism |  | 222,791 |  | 0.09 (-0.35, 0.53) | 0.700 | 0.882 |  | -0.07 (-0.54, 0.39) | 0.754 | 0.947 |
| 119 | Varicose veins of lower extremity |  | 322,454 |  | 0.04 (-0.33, 0.41) | 0.819 | 0.945 |  | 0.06 (-0.33, 0.45) | 0.750 | 0.947 |
| 120 | Hemorrhoids |  | 582,380 |  | 0.12 (-0.16, 0.40) | 0.410 | 0.794 |  | 0.02 (-0.28, 0.31) | 0.918 | 0.979 |
| 121 | ther diseases of veins and lymphatics |  | 232,594 |  | -0.02 (-0.43, 0.39) | 0.922 | 0.974 |  | 0.13 (-0.31, 0.57) | 0.554 | 0.947 |
| 122 | Pneumonia (except that caused by tuberculosis or sexually transmitted disease) |  | 4,838,952 |  | 0.38 (0.26, 0.50) | <0.001* | <0.001* |  | 0.28 (0.14, 0.41) | <0.001* | 0.006* |
| 123 | Influenza |  | 18,139 |  | -0.95 (-2.76, 0.86) | 0.302 | 0.782 |  | -1.24 (-3.41, 0.92) | 0.261 | 0.819 |
| 124 | Acute and chronic tonsillitis |  | 1,098,734 |  | -0.06 (-0.28, 0.16) | 0.585 | 0.844 |  | -0.10 (-0.33, 0.13) | 0.416 | 0.888 |
| 125 | Acute bronchitis |  | 1,264,828 |  | 0.51 (0.27, 0.76) | <0.001* | 0.002* |  | 0.45 (0.19, 0.72) | <0.001* | 0.036* |
| 126 | Other upper respiratory infections |  | 1,578,611 |  | 0.32 (0.11, 0.53) | 0.003* | 0.070 |  | 0.26 (0.04, 0.48) | 0.019* | 0.336 |
| 127 | Chronic obstructive pulmonary disease and bronchiectasis |  | 2,752,048 |  | 0.48 (0.29, 0.66) | <0.001* | <0.001* |  | 0.32 (0.13, 0.50) | 0.001* | 0.036* |
| 128 | Asthma |  | 352,056 |  | 0.86 (0.48, 1.24) | <0.001* | <0.001* |  | 0.91 (0.52, 1.30) | <0.001* | 0.001* |
| 129 | Aspiration pneumonitis; food/vomitus |  | 37,475 |  | -0.44 (-1.44, 0.56) | 0.391 | 0.783 |  | -0.79 (-1.85, 0.28) | 0.149 | 0.759 |
| 130 | Pleurisy; pneumothorax; pulmonary collapse |  | 383,664 |  | 0.04 (-0.28, 0.37) | 0.789 | 0.939 |  | -0.02 (-0.38, 0.34) | 0.915 | 0.979 |
| 131 | Respiratory failure; insufficiency; arrest (adult) |  | 107,162 |  | 0.98 (0.32, 1.63) | 0.003* | 0.079 |  | 0.97 (0.28, 1.66) | 0.006* | 0.160 |
| 132 | Lung disease due to external agents |  | 88,708 |  | -0.60 (-1.52, 0.32) | 0.204 | 0.701 |  | -0.68 (-1.64, 0.27) | 0.162 | 0.782 |
| 133 | Other lower respiratory disease |  | 1,824,000 |  | 0.14 (-0.04, 0.32) | 0.118 | 0.692 |  | 0.02 (-0.17, 0.21) | 0.824 | 0.968 |
| 134 | Other upper respiratory disease |  | 1,196,109 |  | 0.24 (0.01, 0.47) | 0.038* | 0.405 |  | 0.09 (-0.12, 0.30) | 0.393 | 0.870 |
| 135 | Intestinal infection |  | 305,833 |  | 0.27 (-0.16, 0.69) | 0.215 | 0.701 |  | -0.05 (-0.47, 0.37) | 0.820 | 0.968 |
| 136 | Disorders of teeth and jaw |  | 280,120 |  | -0.25 (-0.67, 0.17) | 0.248 | 0.728 |  | -0.35 (-0.82, 0.12) | 0.145 | 0.759 |
| 137 | Diseases of mouth; excluding dental |  | 272,721 |  | 0.22 (-0.21, 0.66) | 0.308 | 0.782 |  | 0.13 (-0.33, 0.59) | 0.587 | 0.947 |
| 138 | Esophageal disorders |  | 330,857 |  | -0.12 (-0.52, 0.29) | 0.569 | 0.844 |  | -0.27 (-0.71, 0.17) | 0.233 | 0.819 |
| 139 | Gastroduodenal ulcer (except hemorrhage) |  | 384,425 |  | -0.30 (-0.63, 0.03) | 0.076 | 0.552 |  | -0.41 (-0.76, -0.07) | 0.020* | 0.336 |
| 140 | Gastritis and duodenitis |  | 1,183,930 |  | 0.12 (-0.07, 0.32) | 0.210 | 0.701 |  | 0.08 (-0.15, 0.32) | 0.489 | 0.926 |
| 141 | Other disorders of stomach and duodenum |  | 461,535 |  | 0.02 (-0.29, 0.33) | 0.899 | 0.969 |  | 0.01 (-0.34, 0.36) | 0.951 | 0.979 |
| 142 | Appendicitis and other appendiceal conditions |  | 1,005,200 |  | 0.06 (-0.16, 0.27) | 0.604 | 0.844 |  | 0.04 (-0.19, 0.26) | 0.730 | 0.947 |
| 143 | Abdominal hernia |  | 1,120,233 |  | -0.36 (-0.62, -0.11) | 0.006* | 0.110 |  | -0.37 (-0.65, -0.09) | 0.010* | 0.201 |
| 144 | Regional enteritis and ulcerative colitis |  | 159,991 |  | 0.32 (-0.15, 0.80) | 0.180 | 0.701 |  | 0.30 (-0.22, 0.83) | 0.257 | 0.819 |
| 145 | Intestinal obstruction without hernia |  | 637,056 |  | 0.10 (-0.16, 0.36) | 0.437 | 0.796 |  | 0.06 (-0.21, 0.33) | 0.670 | 0.947 |
| 146 | Diverticulosis and diverticulitis |  | 16,494 |  | -0.77 (-2.17, 0.63) | 0.282 | 0.782 |  | -0.70 (-2.20, 0.80) | 0.362 | 0.831 |
| 147 | Anal and rectal conditions |  | 613,938 |  | -0.11 (-0.41, 0.19) | 0.478 | 0.811 |  | -0.15 (-0.45, 0.16) | 0.346 | 0.819 |
| 148 | Peritonitis and intestinal abscess |  | 105,973 |  | 0.09 (-0.60, 0.77) | 0.807 | 0.942 |  | -0.23 (-0.95, 0.49) | 0.529 | 0.930 |
| 149 | Biliary tract disease |  | 2,535,207 |  | -0.09 (-0.26, 0.09) | 0.338 | 0.782 |  | -0.12 (-0.30, 0.06) | 0.201 | 0.819 |
| 150 | Liver disease; alcohol-related |  | 94,037 |  | -0.03 (-0.66, 0.60) | 0.929 | 0.976 |  | -0.28 (-0.95, 0.40) | 0.424 | 0.888 |
| 151 | Other liver diseases |  | 1,398,024 |  | 0.19 (-0.00, 0.39) | 0.051 | 0.449 |  | 0.07 (-0.13, 0.27) | 0.504 | 0.929 |
| 152 | Pancreatic disorders (not diabetes) |  | 574,406 |  | -0.25 (-0.57, 0.08) | 0.140 | 0.701 |  | -0.22 (-0.56, 0.12) | 0.212 | 0.819 |
| 153 | Gastrointestinal hemorrhage |  | 811,588 |  | 0.15 (-0.08, 0.38) | 0.194 | 0.701 |  | 0.05 (-0.20, 0.30) | 0.711 | 0.947 |
| 154 | Noninfectious gastroenteritis |  | 690,820 |  | 0.18 (-0.10, 0.46) | 0.214 | 0.701 |  | 0.00 (-0.32, 0.32) | 0.999 | 0.999 |
| 155 | Other gastrointestinal disorders |  | 943,699 |  | -0.21 (-0.41, 0.00) | 0.053 | 0.449 |  | -0.21 (-0.44, 0.03) | 0.091 | 0.617 |
| 156 | Nephritis; nephrosis; renal sclerosis |  | 825,703 |  | 0.06 (-0.17, 0.28) | 0.626 | 0.853 |  | -0.01 (-0.25, 0.24) | 0.960 | 0.979 |
| 157 | Acute and unspecified renal failure |  | 204,743 |  | 0.55 (0.04, 1.06) | 0.034* | 0.405 |  | 0.61 (0.08, 1.14) | 0.024* | 0.371 |
| 158 | Chronic renal failure |  | 1,324,204 |  | 0.08 (-0.15, 0.31) | 0.500 | 0.836 |  | -0.04 (-0.28, 0.21) | 0.779 | 0.967 |
| 159 | Urinary tract infections |  | 478,841 |  | 0.13 (-0.18, 0.44) | 0.423 | 0.796 |  | 0.08 (-0.26, 0.42) | 0.640 | 0.947 |
| 160 | Calculus of urinary tract |  | 912,475 |  | 0.35 (0.13, 0.57) | 0.002* | 0.053 |  | 0.31 (0.09, 0.54) | 0.007* | 0.160 |
| 161 | Other diseases of kidney and ureters |  | 893,240 |  | 0.48 (0.25, 0.71) | <0.001* | 0.002* |  | 0.47 (0.21, 0.73) | <0.001* | 0.025* |
| 162 | Other diseases of bladder and urethra |  | 158,245 |  | 0.13 (-0.37, 0.63) | 0.605 | 0.844 |  | -0.02 (-0.54, 0.51) | 0.953 | 0.979 |
| 163 | Genitourinary symptoms and ill-defined conditions |  | 272,596 |  | 0.01 (-0.40, 0.42) | 0.960 | 0.998 |  | -0.04 (-0.45, 0.37) | 0.864 | 0.979 |
| 164 | Hyperplasia of prostate |  | 519,136 |  | -0.19 (-0.48, 0.09) | 0.182 | 0.701 |  | -0.22 (-0.52, 0.08) | 0.148 | 0.759 |
| 165 | Inflammatory conditions of male genital organs |  | 118,237 |  | -0.56 (-1.15, 0.03) | 0.063 | 0.518 |  | -0.48 (-1.10, 0.14) | 0.127 | 0.748 |
| 166 | Other male genital disorders |  | 510,115 |  | -0.42 (-0.80, -0.03) | 0.035* | 0.405 |  | -0.35 (-0.73, 0.04) | 0.076 | 0.569 |
| 167 | Nonmalignant breast conditions |  | 547,288 |  | -0.04 (-0.33, 0.25) | 0.772 | 0.930 |  | -0.05 (-0.36, 0.26) | 0.743 | 0.947 |
| 168 | Inflammatory diseases of female pelvic organs |  | 511,738 |  | 0.17 (-0.16, 0.50) | 0.314 | 0.782 |  | 0.20 (-0.17, 0.57) | 0.300 | 0.819 |
| 169 | Endometriosis |  | 423,108 |  | -0.16 (-0.50, 0.18) | 0.358 | 0.782 |  | -0.17 (-0.53, 0.18) | 0.341 | 0.819 |
| 170 | Prolapse of female genital organs |  | 124,311 |  | -0.36 (-0.93, 0.20) | 0.206 | 0.701 |  | -0.60 (-1.19, -0.00) | 0.050* | 0.513 |
| 171 | Menstrual disorders |  | 223,970 |  | 0.20 (-0.24, 0.63) | 0.372 | 0.782 |  | 0.18 (-0.28, 0.64) | 0.443 | 0.895 |
| 172 | Ovarian cyst |  | 226,405 |  | -0.40 (-0.86, 0.06) | 0.089 | 0.601 |  | -0.55 (-1.05, -0.05) | 0.031* | 0.415 |
| 173 | Menopausal disorders |  | 20,765 |  | -0.44 (-1.85, 0.97) | 0.539 | 0.844 |  | -0.77 (-2.26, 0.72) | 0.310 | 0.819 |
| 174 | Female infertility |  | 208,894 |  | 0.54 (-0.10, 1.18) | 0.096 | 0.602 |  | 0.61 (-0.15, 1.37) | 0.119 | 0.719 |
| 175 | Other female genital disorders |  | 1,005,624 |  | -0.00 (-0.23, 0.23) | 0.991 | 1.000 |  | -0.04 (-0.28, 0.19) | 0.723 | 0.947 |
| 197 | Skin and subcutaneous tissue infections |  | 352,100 |  | 0.16 (-0.16, 0.49) | 0.324 | 0.782 |  | 0.08 (-0.28, 0.43) | 0.668 | 0.947 |
| 198 | Other inflammatory condition of skin |  | 314,154 |  | 0.05 (-0.36, 0.46) | 0.811 | 0.942 |  | -0.07 (-0.48, 0.33) | 0.726 | 0.947 |
| 199 | Chronic ulcer of skin |  | 61,605 |  | 0.20 (-0.54, 0.93) | 0.600 | 0.844 |  | 0.04 (-0.75, 0.82) | 0.927 | 0.979 |
| 200 | Other skin disorders |  | 571,424 |  | 0.02 (-0.30, 0.34) | 0.911 | 0.974 |  | -0.08 (-0.39, 0.22) | 0.594 | 0.947 |
| 201 | Infective arthritis and osteomyelitis (except that caused by tuberculosis or sexually transmitted disease) |  | 161,280 |  | 0.01 (-0.50, 0.51) | 0.977 | 1.000 |  | -0.11 (-0.65, 0.42) | 0.684 | 0.947 |
| 202 | Rheumatoid arthritis and related disease |  | 412,276 |  | -0.17 (-0.50, 0.15) | 0.295 | 0.782 |  | -0.11 (-0.45, 0.23) | 0.527 | 0.930 |
| 203 | Osteoarthritis |  | 433,178 |  | -0.07 (-0.43, 0.28) | 0.678 | 0.873 |  | -0.15 (-0.52, 0.22) | 0.419 | 0.888 |
| 204 | Other non-traumatic joint disorders |  | 214,644 |  | 0.00 (-0.44, 0.44) | 0.998 | 1.000 |  | -0.08 (-0.54, 0.39) | 0.738 | 0.947 |
| 205 | Spondylosis; intervertebral disc disorders; other back problems |  | 2,187,055 |  | 0.11 (-0.07, 0.29) | 0.236 | 0.720 |  | 0.02 (-0.17, 0.20) | 0.864 | 0.979 |
| 206 | Osteoporosis |  | 140,678 |  | -0.11 (-0.62, 0.39) | 0.659 | 0.869 |  | -0.26 (-0.80, 0.28) | 0.347 | 0.819 |
| 207 | Pathological fracture |  | 114,539 |  | 0.23 (-0.33, 0.79) | 0.427 | 0.796 |  | -0.03 (-0.63, 0.57) | 0.912 | 0.979 |
| 208 | Acquired foot deformities |  | 15,801 |  | -0.37 (-1.99, 1.25) | 0.654 | 0.869 |  | 0.05 (-1.65, 1.74) | 0.958 | 0.979 |
| 209 | Other acquired deformities |  | 174,068 |  | 0.42 (-0.20, 1.04) | 0.187 | 0.701 |  | 0.42 (-0.27, 1.12) | 0.231 | 0.819 |
| 210 | Systemic lupus erythematosus and connective tissue disorders |  | 593,865 |  | -0.06 (-0.32, 0.20) | 0.663 | 0.869 |  | -0.10 (-0.41, 0.22) | 0.548 | 0.947 |
| 211 | Other connective tissue disease |  | 473,131 |  | 0.03 (-0.27, 0.32) | 0.855 | 0.957 |  | -0.02 (-0.34, 0.29) | 0.895 | 0.979 |
| 212 | Other bone disease and musculoskeletal deformities |  | 189,225 |  | -0.32 (-0.82, 0.18) | 0.211 | 0.701 |  | -0.38 (-0.90, 0.15) | 0.158 | 0.782 |

Results are presented as point estimates and 95% CIs of the percentage increase in daily hospital admissions associated with a 10-μg/m^3^ increase in O_3_. Minor disease categories are based on the Clinical Classifications Software (CCS). The 4-day moving average exposure (lag 0-3) was used as the exposure metric of O_3_. In single-pollutant models, the effects of O_3_ were estimated without adjustment for co-pollutants; in two-pollutant models, the effects of O_3_ were estimated after adjustment for PM_2.5_. The Benjamini-Hochberg procedure was applied to adjust the *P* values across 188 minor disease categories; both unadjusted and adjusted *P* values are reported.

* Statistically significant estimate (*P* < 0.05).
