# Supplementary material for: Ambient air pollution and cause-specific risk of hospital admission in China: A nationwide time-series study
Source: PLoS Med. 2020 Aug 6;17(8):e1003188. doi: 10.1371/journal.pmed.1003188 (PMC7410211; doi:10.1371/journal.pmed.1003188)
Supplement: S20 Table — (DOCX) [file pmed.1003188.s034.docx]

# S20 Table. Percent change in hospital admissions per 10-μg/m^3^ increase in PM_2.5_ and O_3_ for eight negative control outcomes, on average across all cities.

|  |  |  | Percent change in admissions per  10-μg/m^3^ increase in PM_2.5_, % | | | | |  | Percent change in admissions per  10-μg/m^3^ increase in O_3_, % | | | | |
| --- | --- | --- | --- | --- | --- | --- | --- | --- | --- | --- | --- | --- | --- |
|  |  |  | Single-pollutant model | |  | Two-pollutant model | |  | Single-pollutant model | |  | Two-pollutant model | |
| CCS code | Disease category |  | Point estimate  (95% CI) | *P* value |  | Point estimate  (95% CI) | *P* value |  | Point estimate  (95% CI) | *P* value |  | Point estimate  (95% CI) | *P* value |
| 223 | Birth trauma |  | -1.33 (-2.93, 0.26) | 0.101 |  | -1.80 (-3.71, 0.10) | 0.063 |  | -0.61 (-2.22, 1.01) | 0.461 |  | -0.82 (-2.63, 0.99) | 0.373 |
| 225 | Joint disorders and dislocations; trauma-related |  | -0.01 (-0.25, 0.24) | 0.960 |  | 0.02 (-0.27, 0.31) | 0.885 |  | 0.05 (-0.23, 0.34) | 0.713 |  | 0.15 (-0.15, 0.44) | 0.322 |
| 227 | Spinal cord injury |  | 0.09 (-0.47, 0.65) | 0.750 |  | -0.17 (-0.86, 0.52) | 0.623 |  | -0.01 (-0.60, 0.57) | 0.965 |  | -0.18 (-0.80, 0.44) | 0.563 |
| 232 | Sprains and strains |  | -0.03 (-0.42, 0.36) | 0.886 |  | 0.16 (-0.31, 0.62) | 0.506 |  | -0.03 (-0.51, 0.45) | 0.907 |  | 0.03 (-0.49, 0.55) | 0.916 |
| 241 | Poisoning by psychotropic agents |  | 0.61 (-0.22, 1.43) | 0.150 |  | 0.65 (-0.32, 1.62) | 0.190 |  | -0.22 (-1.22, 0.78) | 0.668 |  | -0.40 (-1.45, 0.65) | 0.455 |
| 242 | Poisoning by other medications and drugs |  | 0.21 (-0.38, 0.79) | 0.488 |  | 0.40 (-0.27, 1.08) | 0.245 |  | -0.24 (-0.97, 0.48) | 0.512 |  | -0.39 (-1.12, 0.33) | 0.288 |
| 254 | Rehabilitation care; fitting of prostheses; and adjustment of devices |  | 0.09 (-0.17, 0.36) | 0.497 |  | 0.07 (-0.30, 0.44) | 0.711 |  | -0.05 (-0.33, 0.23) | 0.728 |  | 0.02 (-0.28, 0.32) | 0.900 |
| 256 | Medical examination/evaluation |  | 0.00 (-0.76, 0.76) | 0.997 |  | 0.01 (-1.28, 1.29) | 0.992 |  | -0.03 (-0.86, 0.79) | 0.936 |  | -0.01 (-0.95, 0.92) | 0.975 |

Results are presented as point estimates and 95% CIs of the percentage increase in daily hospital admissions associated with a 10-μg/m^3^ increase in PM_2.5_ and O_3_. The single-day exposure on the same day (lag 0) was used as the exposure metric of PM_2.5_. The 2-day moving average exposure (lag 0-1) was used as the exposure metric of O_3_. In single-pollutant models, the effects of PM_2.5_ and O_3_ were estimated without adjustment for co-pollutants; in two-pollutant models, the effects of PM_2.5_ were estimated after adjustment for O_3_, and the effects of O_3_ were estimated after adjustment for PM_2.5_. The *P* values were not adjusted for multiple comparisons.
