# Supplementary material for: Ambient air pollution and cause-specific risk of hospital admission in China: A nationwide time-series study
Source: PLoS Med. 2020 Aug 6;17(8):e1003188. doi: 10.1371/journal.pmed.1003188 (PMC7410211; doi:10.1371/journal.pmed.1003188)
Supplement: Attachment — Submitted filename: gu.pdf [file pmed.1003188.s035.pdf]

This paper examines the association between air pollution measures and a range of diseases in urban China.

I was asked for a statistical report and I interpret that to include all aspects of the design and conduct of the study.

Points of detail

Page 6 I am not quite sure what the phrase 'same below' means. Is this saying that the other smooths were also cubic splines? If so perhaps a re–write would clarify. Supplementary Text S1 is clearer about this.

Page 6 The authors have categorised age into only two categories. Categorising an essentially continuous variable wastes information (Altman and Royston, 2006; Royston et al., 2006) and leads to models which are often implausible as they predict the effect remaining flat within categories and then jumping to a new value at the category boundary. Is useful information being lost here? Dividing an enormous country like China into only two regions seems rather wasteful too.

Page 7 On first read through I found some of the statistical methods quite hard to grasp but Supplementary Text S1 is much clearer so I think I might move the mention of S1 text further forward so people who miss a detail in the main text already know where to look.

Page 9 The issue of lag choice is an important one and one which bridges the divide between statistics and the underlying patho–physiology. From a statistical point of view choosing a common lag seems attractive but is it plausible given the mechanisms of causation of the diseases?

Supplementary Text 1, Page 3 I did not see the three pollutant models reported elsewhere. Given that there seem to be correlations between some of the pollutants (as the authors report in Table S3) perhaps the three pollutant models are of limited interest. Specifically only O_3 seems uncorrelated with the others.

Supplementary Text 1, Page 4 I think I would do a meta–regression here rather than stratifying but I would not want to insist on that.

Supplementary Text 1, Page 4 Perhaps cite the packages? It is the only credit package authors get.

Supplementary Figure 1 The boundaries between the regions are very faint so if they are important they need a bit more ink.

Supplementary Figures 2 and 3 I think the caption should tell us what Lag=0–1 and so on mean. I can see the authors reasoning that S2 shows lag 0 to be a reasonable choice but I would have to say that S3 is much less convincing to me.

For understandable reasons this is about urban China not all China. I am not sure whether this needs stressing more.

Point of more substance

I am not an expert in the role of air pollution but is it plausible that such a wide range of conditions should all be acutely affected in comparable ways at the same lag? I found Figure S9 interesting precisely because it showed limited evidence of a dose–response effect and the authors do mention in discussion the importance of negative controls. I know the authors have adjusted for multiplicity but even so are some of what remains chance findings? The use of negative controls is important and often neglected and could, I think, be made more of.

Summary

Mostly points for clarification.

Michael Dewey

References

D G Altman and P Royston. The cost of dichotomising continuous variables. *British Medical Journal*, 332:1080, 2006.

P Royston, D G Altman, and W Sauerbrei. Dichotomizing continuous predictors in multiple regression: a bad idea. *Statistics in Medicine*, 25:127–141, 2006.
