# Supplementary material for: Ambient air pollution and cause-specific risk of hospital admission in China: A nationwide time-series study
Source: PLoS Med. 2020 Aug 6;17(8):e1003188. doi: 10.1371/journal.pmed.1003188 (PMC7410211; doi:10.1371/journal.pmed.1003188)
Supplement: Attachment — Submitted filename: Response to Reviewers.docx [file pmed.1003188.s036.docx]

**Manuscript ID: PMEDICINE-D-19-04229**

**Title:** Ambient air pollution and cause-specific risk of hospital admissions in China: A nationwide time-series study

Jiangshao Gu; Ying Shi; Yifang Zhu; Ning Chen; Haibo Wang; Zongjiu Zhang; Ting Chen

**Response letter**

Dear Editor,

We would like to thank the Reviewers and Editors for the insightful review on our manuscript entitled “Ambient air pollution and cause-specific risk of hospital admissions in China: A nationwide time-series study” (PMEDICINE-D-19-04229). We have considered the comments and suggestions carefully, and made revisions to the manuscript. Detailed point-to-point replies to the comments are provided in next pages.

Thank you for the consideration of our revision.

Best regards,

Jiangshao Gu

Ting Chen

**Requests from the editors:**

**[The Editors, Comment #1]**

Abstract ‘risk of many diseases’ – would it be better to say non-communicable diseases? To be more specific; ‘2013 to 2017’ – please state the months; ‘Hospital Quality Monitoring System of China.’ – how many hospitals does this cover? And if it is only certain cities, please state which or at least say mostly in the East / West or whichever. Please add some summary demographic information to the abstract.

Data – please state what the restrictions are.

**[Response to The Editors, Comment #1]**

First of all, we would like to express our gratitude to the Editors for the insightful comments and suggestions.

We appreciate the helpful suggestion. We have revised the Abstract to provide all the information requested. We have provided the Data Availability Statement as follows:

[Data Availability Statement] “Researchers wishing to access the data of this study are required to submit a research proposal to the scientific committee of the Hospital Quality Monitoring System (HQMS) via the Scientific Project Management System (https://spms.hqms.org.cn/), under the regulation of Bureau of Medical Administration, National Health Commission of the People’s Republic of China. The research proposal should address: (1) the background and rationale of the proposed research project; (2) the scope of the relevant data (eg, the list of relevant data items, time range, hospital scope, and the list of relevant ICD-10 codes); (3) the significance of the expected results in terms of informing public health policy makers. The scientific committee of the HQMS will review the research proposal and the data will be available if the proposal is approved.”

**[The Editors, Comment #2]**

At this stage, we ask that you include a short, non-technical Author Summary of your research to make findings accessible to a wide audience that includes both scientists and non-scientists. The Author Summary should immediately follow the Abstract in your revised manuscript. This text is subject to editorial change and should be distinct from the scientific abstract.

**[Response to The Editors, Comment #2]**

We appreciate the helpful suggestion. We have included a Author Summary immediately following the Abstract.

**[The Editors, Comment #3]**

Methods – was written consent provided?

Table 1 – please remove roman numerals as not needed in a table

For non Chinese readers please classify what CCS is and their use

Please provide a table in the main text with demographic information of the participants.

I would remove ‘but also eliminate the publication bias that occurs when 340 multiple outcomes are examined and significant associations are selectively reported’

Did your study have a prospective protocol or analysis plan? Please state this (either way) early in the Methods section.

**[Response to The Editors, Comment #3]**

We appreciate the helpful suggestion. We have explained why this study did not need Institutional Review Board approval or written consent as follows:

[Page 7, Lines 162-165 (the numbers refer to the revised manuscript without marked changes)] “All data used were anonymized and de-identified prior to analysis, under the supervision of Bureau of Medical Administration, National Health Commission of the People’s Republic of China. Because the data were analyzed at the aggregate level with no individual identifiers involved, Institutional Review Board approval or written consent was not required for this study.”

We have removed Roman numerals from all tables and figures.

We have clarified what CCS is and its use as follows:

[Page 7, Lines 155-157] “The CCS is a comprehensive categorization scheme for aggregating diagnosis codes into a manageable number of disease categories on the basis of clinical homogeneity, and has been widely used to examine patterns of specific health conditions [1,2].”

We have provided a table in the main text (Table 1) with demographic information of the participants.

We have removed the second half of that sentence and re-written it in a softened tone as follows:

[Page 28, Lines 492-494] “First, by analyzing all causes of hospitalization rather than a few prespecified outcomes and reporting all results, this study provides evidence for the health effects of air pollution that may have been neglected due to lack of prior knowledge.”

We do not have a written document of analysis plan; however, the methods and models used in this study were determined before the analyses were conducted. We have indicated this in the Methods section (Page 8, Lines 182-183), and transparently described the changes in the analysis in response to peer review comments (Page 9, Lines 212-215, for the additional analyses in six regions of China; Page 9, Lines 225-231, for the additional analyses on eight negative control outcomes).

**Reviewer #1:** Gu and colleagues conducted a national time-series study on the association between air pollution and cause-specific hospital admissions in China. It is ambitious to include a vast number of cause-specific endpoints in one manuscript, but the rationale and biological plausibility should be better presented. The analytic approaches seems sound, and the draft is well prepared. Some of the major conclusions have just been reported in several recent publications (e.g. Tian et al, BMJ 2019, Plos Med 2019). I have several major concerns in data source and methodology, which need to be clarified further. There are other minor points that need to be fixed or improved. My detailed comments are as follows:

**[Reviewer #1, Comment #1]**

Line 90, more information is needed for the data collection, i.e. is it a public available dataset that can be accessed and supervised? The website or URL of it?

**[Response to Reviewer #1, Comment #1]**

First of all, we would like to express our gratitude to the Reviewer for the insightful comments and suggestions.

We appreciate the helpful suggestion. We have provided the Data Availability Statement (it should appear on the first few pages of the generated PDF, and will be published along with the article if accepted) as follows:

[Data Availability Statement] “Researchers wishing to access the data of this study are required to submit a research proposal to the scientific committee of the Hospital Quality Monitoring System (HQMS) via the Scientific Project Management System (https://spms.hqms.org.cn/), under the regulation of Bureau of Medical Administration, National Health Commission of the People’s Republic of China. The research proposal should address: (1) the background and rationale of the proposed research project; (2) the scope of the relevant data (eg, the list of relevant data items, time range, hospital scope, and the list of relevant ICD-10 codes); (3) the significance of the expected results in terms of informing public health policy makers. The scientific committee of the HQMS will review the research proposal and the data will be available if the proposal is approved.”

**[Reviewer #1, Comment #2]**

Line 97, how were the cases diagnosed in the first place? The diagnosed cause of diseases upon hospital admission may be from the discharge diagnosis. In addition, hospital admission is quite dependent on local medical resources (hospital beds, etc) and personal social economic status (health insurance for instance). These factors may introduce large uncertainty in the analysis and may eventually bias the results (such as lag pattern). The authors should add a through discussion on this and compare the situation between China and developed countries.

**[Response to Reviewer #1, Comment #2]**

We appreciate the helpful suggestion. The diagnosed cause of diseases upon hospital admission may be different from the discharge diagnosis. According to a previous survey, the consistency of the admission diagnosis and discharge diagnosis in class 3 hospitals in China in 2017 was 65.54%. The use of discharge diagnosis in this study is the same as previous nationwide studies in China and the US [2-7]. In Western countries, healthcare resource utilization is generally scheduled by appointment. However, there is no general practitioner-based referral system in China [8,9]. Regular outpatient visits and hospital admissions are generally unscheduled and are on a first-come, first-served basis. People usually go to hospital promptly when they develop symptoms and will be admitted immediately if necessary. 95% of the total Chinese population have been covered by social health insurance schemes by the end of 2017 [10]. Therefore, hospital admission records can provide reliable and timely information for the health status of a geographically defined population in China. We have added a thorough discussion on this and compare the situation between China and developed countries as follows:

[Page 6, Lines 127-148 (the numbers refer to the revised manuscript without marked changes)] “The daily hospital admission data during the period January 1, 2013, to December 31, 2017, came from the Hospital Quality Monitoring System (HQMS), a national registration database of electronic inpatient discharge records of class 3 hospitals in China, under the administration of the National Health Commission of the People’s Republic of China. Class 3 hospitals are the highest ranked medical institutions in China’s healthcare system, corresponding to tertiary hospitals in the US and Europe but also providing primary and secondary care to less serious patients. In Western countries, healthcare resource utilization is generally scheduled by appointment. However, there is no general practitioner-based referral system in China [8,9]. Regular outpatient visits and hospital admissions are generally unscheduled and are on a first-come, first-served basis. People usually go to hospital promptly when they develop symptoms and will be admitted immediately if necessary. 95% of the total Chinese population have been covered by social health insurance schemes by the end of 2017 [10]. Therefore, hospital admission records can provide reliable and timely information for the health status of a geographically defined population in China. Since January 1, 2013, class 3 hospitals in China have been mandated to automatically submit inpatient discharge records to the HQMS on a daily basis, in a nationally standardized format. By December 31, 2017, the HQMS has covered 1,001 (67.0%) class 3 hospitals and 1,011,375 (78.4%) hospital beds in 252 Chinese cities. The population, the total number of class 3 hospitals, the number of class 3 hospitals covered by the HQMS, the total number of hospital beds, the number of hospital beds covered by the HQMS, and the coverage rates of class 3 hospitals and hospital beds by the HQMS in each of the 252 cities are presented in S1 Table. Each record describes a hospital stay by 346 information items, from which we extracted the date of admission, sex, age, and the primary discharge diagnosis. The diagnosis is coded in the *International Classification of Diseases, 10th Revision* (ICD-10) by certified professional medical coders at each hospital. Quality control is automatically performed at the time of submission to guarantee the completeness, consistency, and accuracy of data. This database has been used to track the epidemiological situation and trends of several diseases in China [11-13].”

**[Reviewer #1, Comment #3]**

Line 102, my biggest concern for this analysis is the rationale and biological plausibility for the association between air pollution and the endpoints examined. Although the authors cited previous papers that examined the same endpoints or exposures, there's inadequate information provided on the specific mechanisms for all the major categories included in the analysis in both Introduction and Discussion. Please amend. Data availability and clinical meaningfulness is not a convincing objective.

**[Response to Reviewer #1, Comment #3]**

We appreciate the helpful suggestion. We have provided more information on the biological plausibility in the Introduction section and a thorough discussion on the specific mechanisms in the Discussion section as follows:

[Page 5, Lines 106-111] “There are at least three hypotheses to explain the extrapulmonary effects of air pollution. First, inhaled pollutants may induce pulmonary inflammation and oxidative stress, which is sufficient to cause systemic inflammation and oxidative stress [14,15]. Second, the lung autonomic nervous system (ANS) may be provoked by pulmonary exposure, which could then result in ANS imbalance [14]; the levels of stress hormones may also be altered [16]. Third, the pollutants may reach and directly interact with remote organs through some ways, eg, penetrating the alveoli and entering the circulation [17,18].”

[Pages 27-28, Lines 464-491] “The biological mechanisms underlying the associations between air pollution and cardiorespiratory diseases are relatively well understood. Epidemiological and toxicological studies have demonstrated that exposure to particulate air pollution may induce systemic inflammation and oxidative stress, cause autonomic imbalance favoring sympathetic tone, and activate hypothalamic-pituitary-adrenal axis, leading to vasoconstriction, hypertension, tachycardia, reduced heart rate variability, increased plasma viscosity, dyslipidemia, vascular endothelial dysfunction, atherosclerosis progression, platelet aggregation, hypercoagulability, and increased thrombogenesis [14,15,19]. One or more of these mechanistic pathways may be involved in explaining the extrapulmonary effects of air pollution [20,21]. Moreover, there are a series of hypotheses to explain the associations between air pollution and specific non-cardiorespiratory diseases, some of which have been supported by emerging clinical and experimental evidence. Short- and long-term exposures to PM_2.5_ and other air pollutants have been suggested to adversely affect glucose and insulin homeostasis, leading to glucose intolerance, decreased insulin sensitivity, increased serum lipid levels, and finally, a higher risk of diabetes mellitus as well as other metabolic diseases [16,22,23]. Air pollution exposure has been reported to be associated with several key factors in the pathophysiology of central nervous system diseases, including neuro-inflammation and oxidative stress, microglial activation, dopaminergic neuron damage, and cerebrovascular impairment [24,25]. These pathways may also play an important role in the pathogenesis of mental and behavioural disorders [26-28]. Air pollutants can be transported to the gastrointestinal tract by either mucociliary clearance from the lungs or ingestion of polluted food and water, where they may exert direct toxic effects on gastrointestinal epithelial cells and alter gut microbial composition, leading to increased intestinal permeability, altered intestinal immunity, and oxidative stress and inflammatory response [29-31]. The skin, the largest and outermost organ of the body, may be adversely affected by pollutants via direct exposure from the air or indirect exposure from the systemic circulation [32]. The kidney, a highly vascularized organ, is vulnerable to both macro- and micro-vascular dysfunction and thus is likely to be affected through the pathways of air pollution-related cardiovascular toxicity [33,34]. Finally, we postulate that air pollution exposure may increase the pain of various conditions (eg, renal colic caused by kidney stones and chronic back pain) through prostaglandin pathways. Prostaglandins are major proinflammatory mediators which activate and sensitize pain receptors [35,36]. Increased levels of prostaglandins cause more pain. Recent animal and human studies have linked excessive prostaglandins with short- and long-term exposures to PM_2.5_ and other air pollutants [35-37]. Nevertheless, the exact biological mechanisms underlying the reported associations remain elusive and deserve further research.”

**[Reviewer #1, Comment #4]**

Line 108, more information is need for the exposures. For example, is this based on fixed-site monitoring stations? How many monitors are there for each city? If such information is provided in the Appendix, please incorporate and indicate. How about missing rates? I assume that will be a lot of missing in exposure time-series, especially for the early years and the less-developed areas.

**[Response to Reviewer #1, Comment #4]**

We appreciate the helpful suggestion. We have provided more information for the exposures as follows:

[Page 7, Lines 166-177] “The daily air pollution data came from the National Air Pollution Monitoring System (http://106.37.208.233:20035/), administered by China’s Ministry of Ecology and Environment. The number of monitoring stations in a city ranges from 1 to 17, with a median of 4. These stations are mandated not to be situated in the neighborhood of distinct emission sources (including but not limited to traffic, industry, and open burning). All measurement procedures are in accordance with China’s Ambient Air Quality Standards (GB3095-2012). For each city, we derived daily 24-hour average concentrations of PM_2.5_, SO_2_, NO_2_, and CO and maximum 8-hour average concentrations of O_3_ on average across all valid monitoring sites, to represent the population exposure to ambient air pollution. We also collected daily mean temperature and mean relative humidity for each city from the China Meteorological Data Sharing Service System (http://data.cma.cn/). During the study period, the missing rates were 1.69% for PM_2.5_, 1.72% for O_3_, 1.68% for SO_2_, 1.67% for NO_2_, 1.72% for CO, 0.70% for temperature, and 0.70% for relative humidity, respectively. Days with missing monitoring measurements were excluded from analysis.”

The missing rates in different years were quite random. For example, the missing rates for PM_2.5_ were 0.46% in 2013, 0.65% in 2014, 4.33% in 2015, 0.05% in 2016, and 0.55% in 2017; while the missing rates for temperature were 0.57% in 2013, 1.68% in 2014, 0.72% in 2015, 0.40% in 2016, and 0.14% in 2017. All of these missing rates are quite acceptable for the time-series design.

**[Reviewer #1, Comment #5]**

Line 116, how were the statistical models determined? If modeling specification is based on previous studies, please cite them and explain the rationale to general readers. Why were the over dispersed Poisson family used, rather than the quasi-Poisson conjunction? What models exactly? Is linear regression models used?

**[Response to Reviewer #1, Comment #5]**

We appreciate the helpful suggestion. Our modeling specification was based on previous studies. We have clarified this as follows:

[Page 8, Lines 180-182] “We applied a two-stage analytic approach to assess the associations between short-term exposure to air pollutants and daily hospital admissions for each disease category, which has been widely used in previous multisite time-series studies [38-43].”

[Page 8, Lines 186-187] “Specifically, we introduced these covariates in the models, which were prespecified according to previously published studies [6,8,9,38-47]”

[Page 8, Lines 191-193] “The *df* values for calendar day, temperature, and relative humidity were selected based on the parameters used in previous published studies [6,8,9,38-47], and were further tested by sensitivity analyses.”

[Page 9, Lines 221-225] “As sensitivity analyses, we changed the *df* for the smooth function of calendar day in the range of 6 to 14 per year, for temperature in the range of 3 to 9, and for relative humidity in the range of 3 to 9. These ranges were chosen in consistency with previous nationwide studies [6,8,9,41,46,47]; we specially tested if the associations remained after more aggressive adjustment for potential confounding effects of the time trend and weather conditions (ie, using more *df*).”

The terms “overdispersed Poisson regression” and “quasi-Poisson regression” refer to exactly the same thing and have been used interchangeably in the literature [6,9,41-43,46,48,49]. We have replaced “overdispersed Poisson” with “quasi-Poisson” throughout the manuscript considering that the readers of *PLOS Medicine* may be more familiar with the term “quasi-Poisson”.

**[Reviewer #1, Comment #6]**

Line 118, how were the degree of freedom determined in the first place? By AIC, BIC or other model fitting statistics?

**[Response to Reviewer #1, Comment #6]**

We appreciate the helpful suggestion. We have clarified this as follows:

[Page 8, Lines 191-193] “The *df* values for calendar day, temperature, and relative humidity were selected based on the parameters used in previous published studies [6,8,9,38-47], and were further tested by sensitivity analyses.”

**[Reviewer #1, Comment #7]**

Line 121, why two terms of temperature and relative humidity were added? I assume this would introduce large collinearity in the models and cause overfitting. Is the best-of-fit modelling strategy applied? What model-fitting statistics were used?

**[Response to Reviewer #1, Comment #7]**

We appreciate the helpful suggestion. The use of two terms of temperature and relative humidity at lag 0 and lag 1-3 in this study is exactly the same as previous multisite time-series studies [38-40]. Using one term of temperature and relative humidity (eg, at lag 0 or lag 0-3) leads to virtually identical results, so it does not really matter. We have indicated this in the main text (Page 8, Lines 186-187).

**[Reviewer #1, Comment #8]**

Line 129, the main lag should be determined by how the models are fitted, especially for multi-center studies, rather than based on which to generate the largest effect estimate.

**[Response to Reviewer #1, Comment #8]**

We appreciate the helpful suggestion. According to all comments from Reviewers, it seems that the selection of the main lag is the most important issue. We would like to first explain why we decided to define a main lag as the lag that generally leaded to strongest effect estimates. There are three commonly used strategies for reporting the results of time-series studies of air pollution and health:

(1) Predefine a main lag according to previously published multisite studies. For example, see [6,41-43,48-50]. It should be noted that all these papers stated that they selected lag 0 or lag 0-1 as the main lag “because it often produced the largest effect estimate in previous studies”.

(2) Select the main lag using model fitting statistics like AIC or BIC. However, a statistical paper focusing on model choice in time-series studies suggested that “model selection methods (including AIC and BIC) which optimize prediction may not be suitable for obtaining an estimate with small bias”, since they are designed to choose the model that will lead to optimal prediction of the outcome series, not necessarily to accurate estimation of *β* (Peng, R. D., Dominici, F., & Louis, T. A. (2006). Model choice in time series studies of air pollution and mortality. Journal of the Royal Statistical Society: Series A (Statistics in Society), 169(2), 179-203.).

(3) Just report all results using all lag structures, without specifying a “main lag”.

In our study, we used the strategy 3 to report the results of 14 major disease categories, so as to provide our readers with as much information as possible. However, it is very difficult to do so for 188 minor disease categories, just because there are too many results. Nevertheless, it seems that our readers are very interested in these results. Therefore, in this revision, we have made substantial efforts to present all results of 188 minor disease categories using all lag structures in 14 tables (S6 to S19 Table), and summarized the overall results in the main text (Page 13, Lines 272-276). Consequently, “main lag” no longer exists in this revision. When we have to first specify a lag so that we can examine the data along another dimension (eg, in subgroup analyses and sensitivity analyses), we have used lag 0-1 for the analyses of O_3_’s effects instead of lag 1, according to the suggestion of Reviewers; however, the results are almost unchanged (the only change is the observation of a negative association between O_3_ and essential hypertension in two-pollutant models, which is supported by previous studies [51]).

**[Reviewer #1, Comment #9]**

Line 134, how were north and south regions separated in China, by latitude?

**[Response to Reviewer #1, Comment #9]**

We appreciate the helpful suggestion. The north and south regions are based on the Huai River-Qinling Mountain line (ie, the 33th parallel of north latitude). We have indicated this in the Methods section (Page 9, Lines 209-210). Moreover, according to the suggestion of Reviewers, we further divided China into six regions based on geography, climate, and culture (Middle-north, Northeast, East, Middle-south, Southwest, and Northwest), and repeated the main analyses in each region to obtain the regional average estimates. S1 Fig has been repainted to illustrate the two- and six-region divisions and the number of cities in each region.

**[Reviewer #1, Comment #10]**

Line 137, there's too little information on how the exposure-response curves were pooled, please provide a brief introduction. In particular, did the authors use a linear model for plotting, how is non-linearity considered for the associations between exposure and outcome?

**[Response to Reviewer #1, Comment #10]**

We appreciate the helpful suggestion. We have provided a brief introduction to the method we used to plot the exposure-response curves as follows:

[Page 9, Lines 215-220] “We developed the exposure-response curves of the relationship between air pollution and hospitalization at the national level as was done in previous studies [52,53]. Briefly, we replaced the linear term for air pollutant in the first-stage regression model by a natural cubic spline smoother with 3 *df* (technically, the coefficient of log RR was replaced by three coefficients used to represent the spline, and the standard error of log RR was replaced by a 3 × 3 variance-covariance matrix). We then conducted a multivariate random-effect meta-analysis to generate the national average estimate of the exposure-response curve.”

**[Reviewer #1, Comment #11]**

Line 138, please indicate what dfs were used for each parameters, and why did the authors select these dfs rather than other ranges? For example, why only 8 to 12 for year, while other studies have reported 4 to 12. This should be stated for temperature and relative humidity as well.

**[Response to Reviewer #1, Comment #11]**

We appreciate the helpful suggestion. In this revision, we have reported the results of sensitivity analyses with the degrees of freedom (*df*) for the time trend ranging from 6 to 14 per year. We have indicated what *df* were used for each parameter, and why we selected these *df* rather than other ranges, as follows:

[Page 9, Lines 221-225] “As sensitivity analyses, we changed the *df* for the smooth function of calendar day in the range of 6 to 14 per year, for temperature in the range of 3 to 9, and for relative humidity in the range of 3 to 9. These ranges were chosen in consistency with previous nationwide studies [6,8,9,41,46,47]; we specially tested if the associations remained after more aggressive adjustment for potential confounding effects of the time trend and weather conditions (ie, using more *df*).”

**[Reviewer #1, Comment #12]**

Line 140, in the disease burden calculation, one critical problem is that this was inherently a time-series study on the short-term associations, thus it is not applicable to estimate the annual (long-term) reduction in hospital admissions. The authors should better clarify this. How is the information acquired on the average cost for each hospitalization in China? I assume this would vary a lot by cities.

**[Response to Reviewer #1, Comment #12]**

We appreciate the helpful suggestion. The words we used here are the same as previous nationwide time-series studies [9,39-41,46]. We have clarified this issue better as follows:

[Page 27, Lines 459-463] “It should be noted that our estimates are based on short-term associations between air pollution and daily hospital admissions, and thus are not comparable to the estimates based on long-term associations, eg, in the Global Burden of Diseases Study [54]; however, our estimates can be compared with those from time-series studies of the same design [41]. Therefore, the precise estimation of the morbidity burden attributable to air pollution in China and worldwide deserves further research.”

The average cost for a hospitalization for each major disease categories in China was collected from China Health and Family Planning Statistical Yearbook 2017. The national average cost per hospitalization was used in calculation (we do not have city-specific cost data). The calculation method we used is the same as previous nationwide time-series studies [9,39-41,46]. We have indicated this in the main text (Page 10, Lines 236-238).

**[Reviewer #1, Comment #13]**

Line 165, why to select different lags for PM2.5 and ozone? How to interpret the results if different lags were set?

**[Response to Reviewer #1, Comment #13]**

We appreciate the helpful suggestion. In view of the differences in physicochemical and toxicological characteristics of PM_2.5_ and O_3_, it is plausible that the lag patterns differ for these two pollutants. In this revision, we have made substantial efforts to present all results of 188 minor disease categories using all lag structures in 14 tables (S6 to S19 Table), and summarized the overall results in the main text (Page 13, Lines 272-276). Please refer to [Response to Reviewer #1, Comment #8] for a thorough discussion.

**[Reviewer #1, Comment #14]**

Line 282, why were the estimates of this study lower than those from developed countries?

**[Response to Reviewer #1, Comment #14]**

We appreciate the helpful suggestion. We have discussed why the estimates of this study were lower than those from developed countries as follows:

[Page 25, Lines 402-411] “The size of effect in this analysis was similar to that of recent multisite studies in China [6,9,41,46], and smaller than the estimates from North America and Europe [39,55,56]. The lower estimates of this study than those from high-income countries may have at least two possible explanations. First, the levels of particulate air pollution were much higher in China than in high-income countries. As observed in our study and previous multisite studies [9,41,46,56], the slopes of exposure-response relationships were generally steeper at lower concentrations than at higher concentrations, which may be partly attributable to larger exposure measurement errors on heavily polluted days when public health intervention would be reinforced and people would more actively take self-protection measures. Second, the chemical profile of PM_2.5_ was different between China and high-income countries. PM_2.5_ in China’s air has a higher proportion of crustal materials, which may have lower toxicity than the components originated from fossil fuel combustion [41,46].”

**[Reviewer #1, Comment #15]**

Line 286, why could this study provide evidence for the chronic health effects of air pollution?

**[Response to Reviewer #1, Comment #15]**

We appreciate the helpful suggestion. We have removed the term “chronic” to make our statement more accurate.

**[Reviewer #1, Comment #16]**

Line 293, Tian's study was on ER visits, thus may not be appropriate for this case.

**[Response to Reviewer #1, Comment #16]**

We appreciate the helpful suggestion. Hospital visits and admissions are generally unscheduled and are on a first-come, first-served basis in mainland China, somewhat similar to the scheme of emergency hospital admissions in Hong Kong. Since there is very limited literature on this topic, Tian’s study seems necessary to be introduced to general readers.

**[Reviewer #1, Comment #17]**

Again, please add through discussions on the rationales between air pollution and various endpoints.

**[Response to Reviewer #1, Comment #17]**

We appreciate the helpful suggestion. We have provided more information on the biological plausibility in the Introduction section and a thorough discussion on the specific mechanisms in the Discussion section. Please refer to [Response to Reviewer #1, Comment #3].

**[Reviewer #1, Comment #18]**

Line 337, the first strength of this study may stand only if there's solid supporting evidence for the hypothesized association.

**[Response to Reviewer #1, Comment #18]**

We appreciate the helpful suggestion. We have removed the second half of that sentence and re-written it in a softened tone as follows:

[Page 28, Lines 492-494] “First, by analyzing all causes of hospitalization rather than a few prespecified outcomes and reporting all results, this study provides evidence for the health effects of air pollution that may have been neglected due to lack of prior knowledge.”

**[Reviewer #1, Comment #19]**

Line 340, the generalizability of this study is largely dependent on whether the hospital admissions are representative enough for the local population. This should be mentioned before in the Methods, in that how many population (maybe in percentage) did the class 3 hospital serve? How many hospitals are there in each city, etc.

**[Response to Reviewer #1, Comment #19]**

We appreciate the helpful suggestion. We have mentioned this in the Methods section as follows:

[Page 6, Lines 138-143] “Since January 1, 2013, class 3 hospitals in China have been mandated to automatically submit inpatient discharge records to the HQMS on a daily basis, in a nationally standardized format. By December 31, 2017, the HQMS has covered 1,001 (67.0%) class 3 hospitals and 1,011,375 (78.4%) hospital beds in 252 Chinese cities. The population, the total number of class 3 hospitals, the number of class 3 hospitals covered by the HQMS, the total number of hospital beds, the number of hospital beds covered by the HQMS, and the coverage rates of class 3 hospitals and hospital beds by the HQMS in each of the 252 cities are presented in S1 Table.”

**Reviewer #2:** The manuscript describes a large multi-city time series study from China on air pollution exposure and numerous hospitalization (morbidity) endpoints. The study made use of national standardized reporting of hospitalization discharges. The data analysis methodology was generally state-of-the-art for multi-site time series and accounted for the multiple exploratory comparisons undertaken. Additional sensitivity analyses were important, although not as helpful in some respects as they might have been - see below. Finally, the manuscript was generally very well-written. I have some concerns, however.

**[Reviewer #2, Comment #1]**

An important first question, given the plethora of published time series studies, including multi-city time series studies, some of which are from China, is whether yet another one is needed. This study has some features to recommend it. The large array of outcomes, while not without issues (see points 3-5, below), is unique, and the large number of hospitalization counts ensures adequate power for addressing many of the outcomes. There may be others that the authors want to highlight to address the specific contributions that justify yet another time series study.

**[Response to Reviewer #2, Comment #1]**

First of all, we would like to express our gratitude to the Reviewer for the insightful comments and suggestions.

We appreciate the helpful suggestion. We deeply thank for the positive comment on our work. Considering all comments and suggestions from the Reviewers and Editors, we have revised the discussion about the strengths of this work as follows:

[Pages 28-29, Lines 492-505 (the numbers refer to the revised manuscript without marked changes)] “This study has some strengths. First, by analyzing all causes of hospitalization rather than a few prespecified outcomes and reporting all results, this study provides evidence for the health effects of air pollution that may have been neglected due to lack of prior knowledge. Second, this study defined the disease categories at two levels. The major disease categories were used to evaluate the overall health effects of air pollution on each organ system, while the minor disease categories were used to identify the specific diseases which can be triggered and/or exacerbated by air pollution exposure. Third, this study has the advantages of a large dataset with good internal consistency in data collection, good timeliness (2013-2017), and extensive geographical and population coverage (252 cities in China; all ages), ensuring reliable generalizability of our results to Chinese population or other populations in similar settings. Fourth, this study also provided the exposure-response relationships between air pollution and cause-specific hospital admissions, which support the hypothesis that there is no safety threshold for the health effects of PM_2.5_ exposure but there may be a safety threshold close to the background concentration of O_3_ for the effect of O_3_ exposure on respiratory diseases. The possible existence of a threshold for O_3_ has been suggested by several studies conducted in high-income countries [53,56], and should be further investigated in low- and middle-income countries where the evidence is still limited.”

**[Reviewer #2, Comment #2]**

Although I may have missed it, there was no mention that the hospitalizations included only those for unscheduled urgent admissions. Scheduled hospitalizations, such as those for "maintenance chemotherapy/radiotherapy," which interestingly showed an association with PM2.5, should not be included in this analysis.

**[Response to Reviewer #2, Comment #2]**

We appreciate the helpful suggestion. Our data cannot distinguish between scheduled and unscheduled hospitalizations, which is a limitation of this work. We have revised the discussion about the limitations of this work as follows:

[Page 29, Lines 513-517] “Third, our data cannot distinguish between scheduled and unscheduled hospitalizations. Including hospital admissions (eg, planned surgeries) unrelated to air pollution is expected to reduce the estimation precision. However, a general practitioner-based referral system is not available in China [8,9]. Hospital visits and admissions are generally unscheduled and are on a first-come, first-served basis. Therefore, the impact of scheduled hospitalizations on our results is expected to be minor.”

The association between PM_2.5_ and hospitalizations for “maintenance chemotherapy/radiotherapy” might be a false positive finding. However, it may be a true association in that the date of some scheduled hospitalizations may be advanced by several days if the patient’s condition (of cancer and/or cardiorespiratory co-morbidities) is exacerbated by short-term exposure to air pollution and needs to be addressed first before a maintenance chemotherapy/radiotherapy can be performed. This association is expected to be weak; however, the large sample size of this category (*n* = 12,173,649) may confer adequate power to detect a weak association. The association was significant only in the single-pollutant model (ie, not very robust), so we did not mention it in the main text. Because this association is not that useful, we may have excluded it from analysis. However, since we have conducted the analysis, maybe it is better to make all results transparent to our readers.

**[Reviewer #2, Comment #3]**

The authors argue that including the large array of hospitalization endpoints was a strength of the study, and have addressed concerns about multiple comparisons. However, with such a large array of outcomes showing statistically significant associations, the issue of biologic plausibility becomes a more serious concern. Interestingly, the authors raise the issue of negative controls when they note: "... negative controls such as female infertility, which has no plausible relation to air pollution" (line 280). One could argue, in fact, that many of the outcomes showing statistically significant associations could be characterized in the same way. For example, there would seem to be tenuous plausibility for calculus of the urinary tract, and certainly none for "maintenance chemotherapy/radiotherapy," as noted above. It's possible that all of the outcomes demonstrating significant effects in this analysis, as the authors suggest, are providing novel insights into the far more global impact of air pollution on health than previously thought, but this seems very unlikely. And while systemic inflammation and oxidative stress as a unifying "downstream" mechanism underlying all air pollution health effects covers a wide swath of diseases, there remain many outcomes for which even this is a tenuous argument, many that heretofore we might even have thought to include as negative controls. Although currently there is no solution to this dilemma, the authors should consider including some very convincing negative controls, such as accidents, for example, which were not included in the current analysis.

**[Response to Reviewer #2, Comment #3]**

We appreciate the helpful suggestion. We have provided more information on the biological plausibility in the Introduction section and a thorough discussion on the specific mechanisms in the Discussion section. Please refer to [Response to Reviewer #1, Comment #3].

For the plausibility of a possible association between PM_2.5_ and calculus of the urinary tract, another support is that Non-Steroidal Anti-Inflammatory Drugs (NSAIDs) alone or in combination with opioids have been used to treat renal colic pain caused by kidney stones for a long time. Analgesic effects of these medications are due to the inhibition of prostaglandin synthesis [57].

We have performed additional analyses on eight negative control outcomes and reported the results as follows:

[Page 9, Lines 225-231] “According to the suggestion of reviewers, we included eight outcomes (CCS categories “birth trauma”, “joint disorders and dislocations; trauma-related”, “spinal cord injury”, “sprains and strains”, “poisoning by psychotropic agents”, “poisoning by other medications and drugs”, “rehabilitation care; fitting of prostheses; and adjustment of devices”, and “medical examination/evaluation”) as negative controls [58], for which no biological or clinical evidence supports an association with air pollution. We repeated the main analyses on these negative control outcomes to examine if our results were substantially biased owing to residual confounding.”

[Page 19, Lines 353-355] “None of the eight negative control outcomes were associated with PM_2.5_ or O_3_ in single- or two-pollutant models, which provides further reassurance that our findings were not substantially confounded or biased (S20 Table).”

**[Reviewer #2, Comment #4]**

One of the hazards of including as many endpoints as were included in this study is the possibility that there may have been a very small number of daily counts for many of the endpoints, and in some cases no counts in some of the cities. This would result in very uncertain estimates of effect in those cases in the first stage of the analysis. The meta-analytic step presumably somewhat accounts for this by giving less weight to endpoints in cities with uncertain estimates. It would nevertheless be helpful to see the distribution of daily counts of each of the endpoints (not merely IQR as in Table 2) as a supplemental table in order to assess the magnitude of this issue.

**[Response to Reviewer #2, Comment #4]**

We appreciate the helpful suggestion. Considering all comments and suggestions from the Reviewers and Editors, we have split the original Table 1 into two new Tables (Table 1 and 2). The new Table 1 provides the demographic characteristics of hospital admissions, and the new Table 2 provides the summary statistics of annual-average daily counts of city-wide hospital admissions (ie, the distribution of daily counts of each of the endpoints). Overall, the daily counts of our study are larger than or comparable to those of many previous multisite time-series studies.

**[Reviewer #2, Comment #5]**

A related issue is that the more specific the diagnostic categories that are used, the greater the likelihood of outcome misclassification. That is, while diagnosis coding accuracy for a general endpoint such as cardiovascular disease is good, diagnosis coding accuracy for more specific endpoints such as pulmonary heart disease or acute bronchitis, for example, are much more suspect. Because of the focus on a large array of very specific outcomes in this study, the likelihood of misclassification of the outcomes is great. The impact of such errors in the outcome when the outcome is binary would be to bias to the null. The authors recognize this, but perhaps somewhat more elaboration in the Discussion is needed.

**[Response to Reviewer #2, Comment #5]**

We appreciate the helpful suggestion. We have revised the discussion about the limitations of this work as follows:

[Page 29, Lines 508-513] “Second, we cannot rule out the possibility of misclassifications resulting from diagnostic or coding errors in this large scale nationwide dataset. Generally, the more specific the diagnostic categories that are used, the greater the likelihood of outcome misclassification. The diagnosis coding accuracy for minor disease categories may thus be lower than that for major disease categories. Therefore, our results concerning minor disease categories should be interpreted with caution. Future studies based on medically reviewed outcome data are warranted to confirm our findings.”

**[Reviewer #2, Comment #6]**

It is well known that reporting the effect estimate from the single day with the largest estimate of effect is biased and results in overestimation of the true effects. The current preferred approach is to employ an unconstrained distributed lag model to estimate the unbiased net cumulative effect over several days, an approach which is arguably also more in line with current pathophysiologic understanding. However, in the current setting, for PM2.5 at least, it seems the choice to report only current day effects is not an unreasonable choice given the day lag structure presented for many of the endpoints (Figure S2). This is not the case for ozone, however. Figure S3 shows that there is no clear single lag that stands out, in contrast to the picture with PM2.5. Even for respiratory disease, the primary condition for which effects of ozone were observed, the choice of lag is not clear - positive effects are observed for day lags 0 through 3 with that for lag 1 being the largest, and with day lag 3 being the longest lag reported. For circulatory disease, alternatively, the current day has the largest estimate of ozone effect. The justification for reporting only lag 1 effects for ozone is therefore suspect. The results for ozone, at least, are an example of why employing a distributed lag model is preferable. Reporting only day 1 lag effects for ozone and respiratory disease, the lag with the largest effect estimate (Figure S3), is not recommended. For most of the other endpoints, reporting only the lag 1 effects for ozone (Fig S3) is an odd choice.

**[Response to Reviewer #2, Comment #6]**

We appreciate the helpful suggestion. In this revision, we have made substantial efforts to present all results of 188 minor disease categories using all lag structures in 14 tables (S6 to S19 Table), and summarized the overall results in the main text (Page 13, Lines 272-276). Please refer to [Response to Reviewer #1, Comment #8] for a thorough discussion.

**[Reviewer #2, Comment #7]**

The concentration-response function (CRF) plots (Figure 3) show 95% confidence bounds that are constrained to have bounds of zero at the point where the endpoint and the PM2.5 axes are both zero. This makes little sense given that there are likely no days with extremely low PM2.5 concentrations in Chinese cities. One expects the confidence bounds to be tightest around the median of the PM2.5 concentration distribution where most of the data lie. Admittedly, what the authors have shown is commonplace in the literature, but it's a practice that should be avoided because it is not informative.

**[Response to Reviewer #2, Comment #7]**

We appreciate the helpful suggestion. We have re-plotted the exposure-response curves as suggested (Fig 3, and S8 and S9 Fig). The vertical scale now represents the relative change from the mean effect of PM_2.5_/O_3_ on daily hospital admissions, rather than the relative change from the effect at an ideal concentration of 0. As expected, the 95% CI is narrower in the middle and wider at the lower/higher end. These E-R curves are definitely more informative than before.

**[Reviewer #2, Comment #8]**

The authors have repeated (line 230) what is commonly stated in the literature regarding the absence of an apparent threshold concentration where no effects can be identified. While this may be the case for PM2.5 and for most of the endpoints, importantly it does not appear to be the case for ozone and respiratory disease (Figure S9), the endpoint of most relevance for ozone. A threshold in the range of 40-50 ug/m3 of ozone seems to be present. The conclusions need to therefore be qualified to reflect this.

**[Response to Reviewer #2, Comment #8]**

We appreciate the helpful suggestion. As suggested, we have revised the Results section as follows:

[Page 19, Lines 340-345] “The results of exposure-response analyses are shown in Fig 3, and S8 and S9 Fig. The exposure-response curves between PM_2.5_ and hospital admissions generally showed steeper slopes at lower concentrations than at higher concentrations, with no safety threshold below which PM_2.5_ exposure had no health effect (Fig 3, and S8 Fig). A threshold in the range of 40-50 μg/m^3^ (nearing the background level of O_3_) was observed in the exposure-response curve between O_3_ and hospital admissions for diseases of the respiratory system (S9 Fig).”

We have revised the Discussion section as follows:

[Pages 28-29, Lines 500-505] “Fourth, this study also provided the exposure-response relationships between air pollution and cause-specific hospital admissions, which support the hypothesis that there is no safety threshold for the health effects of PM_2.5_ exposure but there may be a safety threshold close to the background concentration of O_3_ for the effect of O_3_ exposure on respiratory diseases. The possible existence of a threshold for O_3_ has been suggested by several studies conducted in high-income countries [53,56], and should be further investigated in low- and middle-income countries where the evidence is still limited.”

**[Reviewer #2, Comment #9]**

The results of the several sensitivity analyses were reassuring. However, the sensitivity analysis relating to the degrees of freedom (df) for the time trend is not adequate. Here the authors have reported results of varying the time trend df in the range from 8 to 12/year. This range is far too narrow to assess sensitivity of results to varying df and to be assured that the chosen df are adequate. Clearly, using too few df runs the risk of not adequately removing time trends, while it has been argued that using too many df runs the risk of encroaching on the effect of the air pollutant. Given that the time scale of interest for pollutant effects is from one to a few days, with 12 df/year that corresponds to a knot for approximately each month, there seems little danger that the pollutant effect would be encroached upon by even more than 12 df. The authors should be encouraged to report results of a sensitivity analysis with time trend df ranging from 6 to 18/year.

**[Response to Reviewer #2, Comment #9]**

We appreciate the helpful suggestion. In this revision, we have reported the results of sensitivity analyses with the degrees of freedom (*df*) for the time trend ranging from 6 to 14 per year (Page 19, Lines 346-353). The results of PM_2.5_ remained stable using alternative *df* for the smooth function of calendar day. The results of O_3_ would be substantially biased when using too few *df* for the smooth function of calendar day (eg, the association between O_3_ and hospital admissions for diseases of the respiratory system would even become negative when *df* = 6 per year), indicating that the confounding effects of seasonality and/or long-term trends were not controlled adequately in that case; however, the bias would disappear when *df* ≥ 8 per year, and the results remained stable with increasing *df* up to 14 per year, demonstrating that the models used in the main analyses had successfully removed the confounding effects. The results with *df* for the time trend ranging from 15 to 18 per year are not available temporarily, because of the unprecedented complex difficulties we are facing now against the epidemic of SARS-CoV-2 (2019-nCoV, COVID-19) coronavirus; these results will be available on request from the corresponding author when the epidemic is over. However, we consider that the robustness of our results has been demonstrated in view of the clear trend of stable estimates with increasing *df* from 8 to 14 per year.

**Reviewer #3:** This paper examines the association between air pollution measures and a range of diseases in urban China.

I was asked for a statistical report and I interpret that to include all aspects of the design and conduct of the study.

**[Reviewer #3, Comment #1]**

Page 6 I am not quite sure what the phrase `same below' means. Is this saying that the other smooths were also cubic splines? If so perhaps a re-write would clarify. Supplementary Text S1 is clearer about this.

**[Response to Reviewer #3, Comment #1]**

First of all, we would like to express our gratitude to the Reviewer for the insightful comments and suggestions.

We appreciate the helpful suggestion. We have re-written to clarify that all smooth functions were natural cubic spline smoothers (Page 8, Lines 185-191 [the numbers refer to the revised manuscript without marked changes]).

**[Reviewer #3, Comment #2]**

Page 6 The authors have categorised age into only two categories. Categorising an essentially continuous variable wastes information (Altman and Royston, 2006; Royston et al., 2006) and leads to models which are often implausible as they predict the effect remaining flat within categories and then jumping to a new value at the category boundary. Is useful information being lost here? Dividing an enormous country like China into only two regions seems rather wasteful too.

**[Response to Reviewer #3, Comment #2]**

We appreciate the helpful suggestion. In this revision, we categorised age into three categories (<65, 65-74, and ≥75 years), consistent with previous studies [6,41-43,48-50]. We generally observed higher risks related to PM_2.5_ in older people (65-74 or ≥75 years) than in younger people (<65 years). The difference between 65-74 age group and ≥75 age group was mixed and uncertain.

In this revision, we further divided China into six regions based on geography, climate, and culture (Middle-north, Northeast, East, Middle-south, Southwest, and Northwest) (S1 Fig), and repeated the main analyses in each region to obtain the regional average estimates. However, the results were quite random and we did not observe any useful pattern (S12 and S13 Fig).

**[Reviewer #3, Comment #3]**

Page 7 On first read through I found some of the statistical methods quite hard to grasp but Supplementary Text S1 is much clearer so I think I might move the mention of S1 text further forward so people who miss a detail in the main text already know where to look.

**[Response to Reviewer #3, Comment #3]**

We appreciate the helpful suggestion. According to the suggestion of Reviewers, we have incorporated all contents of S1 Text into the main text, and thus S1 Text no longer exists in this revision.

**[Reviewer #3, Comment #4]**

Page 9 The issue of lag choice is an important one and one which bridges the divide between statistics and the underlying patho-physiology. From a statistical point of view choosing a common lag seems attractive but is it plausible given the mechanisms of causation of the diseases?

**[Response to Reviewer #3, Comment #4]**

We appreciate the helpful suggestion. In this revision, we have made substantial efforts to present all results of 188 minor disease categories using all lag structures in 14 tables (S6 to S19 Table), and summarized the overall results in the main text (Page 13, Lines 272-276). Please refer to [Response to Reviewer #1, Comment #8] for a thorough discussion.

**[Reviewer #3, Comment #5]**

Supplementary Text 1, Page 3 I did not see the three pollutant models reported elsewhere. Given that there seem to be correlations between some of the pollutants (as the authors report in Table S3) perhaps the three pollutant models are of limited interest. Specifically only O3 seems uncorrelated with the others.

**[Response to Reviewer #3, Comment #5]**

We appreciate the helpful suggestion. Three-pollutant models were reported, for example, in a *NEJM* paper [38], which examined the associations between PM_10_ and O_3_ and mortality in 20 U.S. cities. (It should be noted that three-pollutant models were termed “trivariate models” in that paper, and PM_10_ was termed “fine particulate matter”, for historical reason.) In the area of air pollution epidemiology, if the effect of PM changes little and/or remains significant after adjustment for another traffic-related pollutant (eg, SO_2_, NO_2_, or CO), we usually consider that it confirms the robustness of association between PM and health outcomes (although there are moderate correlations between PM and other traffic-related pollutants). The explanation for the rareness of three-pollutant models in the literature might be that most studies treated only one pollutant (say, PM) as the pollutant of interest, in which case reporting two-pollutant models would be natural (say, “PM+SO_2_”, “PM+NO_2_”, “PM+CO”) [42,43,48,49]; while relatively few studies treated both PM and O_3_ as the pollutants of interest, in which case reporting three-pollutant models would be natural, like the *NEJM* paper and our work.

**[Reviewer #3, Comment #6]**

Supplementary Text 1, Page 4 I think I would do a meta-regression here rather than stratifying but I would not want to insist on that.

**[Response to Reviewer #3, Comment #6]**

We appreciate the helpful suggestion. It would be interesting to do a meta-regression. However, since the amount of data reported in this manuscript is already very huge and there are many novel associations that need to be interpreted, maybe it is better to leave meta-regression to our future work.

**[Reviewer #3, Comment #7]**

Supplementary Text 1, Page 4 Perhaps cite the packages? It is the only credit package authors get.

**[Response to Reviewer #3, Comment #7]**

We appreciate the helpful suggestion. We have cited the packages in the main text (Page 10, Lines 241-243).

**[Reviewer #3, Comment #8]**

Supplementary Figure 1 The boundaries between the regions are very faint so if they are important they need a bit more ink.

**[Response to Reviewer #3, Comment #8]**

We appreciate the helpful suggestion. S1 Fig has been greatly enhanced, and the boundaries are much clearer now.

**[Reviewer #3, Comment #9]**

Supplementary Figures 2 and 3 I think the caption should tell us what Lag=0-1 and so on mean. I can see the authors reasoning that S2 shows lag 0 to be a reasonable choice but I would have to say that S3 is much less convincing to me.

**[Response to Reviewer #3, Comment #9]**

We appreciate the helpful suggestion. We have revised the captions of S2 and S3 Fig to tell readers what “Lag 0-1” and so on mean. In this revision, we have made substantial efforts to present all results of 188 minor disease categories using all lag structures in 14 tables (S6 to S19 Table), and summarized the overall results in the main text (Page 13, Lines 272-276). Please refer to [Response to Reviewer #1, Comment #8] for a thorough discussion.

**[Reviewer #3, Comment #10]**

For understandable reasons this is about urban China not all China. I am not sure whether this needs stressing more.

**[Response to Reviewer #3, Comment #10]**

We appreciate the helpful suggestion. Several previous multisite studies on the topic of air pollution and health in China included only urban residents because their data came from the Urban Employee Basic Medical Insurance (UEBMI) [6,7,9,46,50], which covered only urban employed and retired individuals. In contrast, our data came from the Hospital Quality Monitoring System (HQMS), which covered both urban and rural individuals no matter what type of insurance they enrolled in. Previous survey has reported that rural residents accounted for 29.1% to 50.0% (varying by disease) of the cases recorded in the HQMS [12]. Therefore, our results should have generalizability to the total Chinese population.

In the original S1 Text, we used the word “urban” sometimes in the introduction to the National Air Pollution Monitoring System, which was to indicate that the data were at the city level but not that the data only covered urban areas (“city” and “urban” share the same Chinese word). We have corrected this misuse of word.

**[Reviewer #3, Comment #11]**

I am not an expert in the role of air pollution but is it plausible that such a wide range of conditions should all be acutely affected in comparable ways at the same lag? I found Figure S9 interesting precisely because it showed limited evidence of a dose-response effect and the authors do mention in discussion the importance of negative controls. I know the authors have adjusted for multiplicity but even so are some of what remains chance findings? The use of negative controls is important and often neglected and could, I think, be made more of.

**[Response to Reviewer #3, Comment #11]**

We appreciate the helpful suggestion. We have provided more information on the biological plausibility in the Introduction section and a thorough discussion on the specific mechanisms in the Discussion section. Please refer to [Response to Reviewer #1, Comment #3].

We have performed additional analyses on eight negative control outcomes and reported the results as follows:

[Page 9, Lines 225-231] “According to the suggestion of reviewers, we included eight outcomes (CCS categories “birth trauma”, “joint disorders and dislocations; trauma-related”, “spinal cord injury”, “sprains and strains”, “poisoning by psychotropic agents”, “poisoning by other medications and drugs”, “rehabilitation care; fitting of prostheses; and adjustment of devices”, and “medical examination/evaluation”) as negative controls [58], for which no biological or clinical evidence supports an association with air pollution. We repeated the main analyses on these negative control outcomes to examine if our results were substantially biased owing to residual confounding.”

[Page 19, Lines 353-355] “None of the eight negative control outcomes were associated with PM_2.5_ or O_3_ in single- or two-pollutant models, which provides further reassurance that our findings were not substantially confounded or biased (S20 Table).”

**References**

1.	Radley, D.C.; Gottlieb, D.J.; Fisher, E.S.; Tosteson, A.N. Comorbidity risk-adjustment strategies are comparable among persons with hip fracture. *J Clin Epidemiol* **2008**, *61*, 580-587, doi:10.1016/j.jclinepi.2007.08.001.

2.	Bobb, J.F.; Obermeyer, Z.; Wang, Y.; Dominici, F. Cause-specific risk of hospital admission related to extreme heat in older adults. *JAMA* **2014**, *312*, 2659-2667, doi:10.1001/jama.2014.15715.

3.	Liu, H.; Tian, Y.; Xu, Y.; Huang, Z.; Huang, C.; Hu, Y.; Zhang, J. Association between ambient air pollution and hospitalization for ischemic and hemorrhagic stroke in China: A multicity case-crossover study. *Environ Pollut* **2017**, *230*, 234-241, doi:10.1016/j.envpol.2017.06.057.

4.	Liu, H.; Tian, Y.; Xiang, X.; Juan, J.; Song, J.; Cao, Y.; Huang, C.; Li, M.; Hu, Y. Ambient Particulate Matter Concentrations and Hospital Admissions in 26 of China's Largest Cities: A Case-Crossover Study. *Epidemiology* **2018**, *29*, 649-657, doi:10.1097/EDE.0000000000000869.

5.	Liu, H.; Tian, Y.; Xiang, X.; Li, M.; Wu, Y.; Cao, Y.; Juan, J.; Song, J.; Wu, T.; Hu, Y. Association of short-term exposure to ambient carbon monoxide with hospital admissions in China. *Sci Rep* **2018**, *8*, 13336, doi:10.1038/s41598-018-31434-1.

6.	Tian, Y.; Liu, H.; Zhao, Z.; Xiang, X.; Li, M.; Juan, J.; Song, J.; Cao, Y.; Wang, X.; Chen, L., et al. Association between ambient air pollution and daily hospital admissions for ischemic stroke: A nationwide time-series analysis. *PLoS Med* **2018**, *15*, e1002668, doi:10.1371/journal.pmed.1002668.

7.	Tian, Y.; Liu, H.; Xiang, X.; Zhao, Z.; Juan, J.; Li, M.; Song, J.; Cao, Y.; Wu, Y.; Wang, X., et al. Ambient Coarse Particulate Matter and Hospital Admissions for Ischemic Stroke. *Stroke* **2019**, *50*, 813-819, doi:10.1161/STROKEAHA.118.022687.

8.	Tian, Y.; Xiang, X.; Juan, J.; Sun, K.; Song, J.; Cao, Y.; Hu, Y. Fine particulate air pollution and hospital visits for asthma in Beijing, China. *Environ Pollut* **2017**, *230*, 227-233, doi:10.1016/j.envpol.2017.06.029.

9.	Tian, Y.; Liu, H.; Liang, T.; Xiang, X.; Li, M.; Juan, J.; Song, J.; Cao, Y.; Wang, X.; Chen, L., et al. Fine particulate air pollution and adult hospital admissions in 200 Chinese cities: a time-series analysis. *Int J Epidemiol* **2019**, 10.1093/ije/dyz106, doi:10.1093/ije/dyz106.

10.	Meng, Q.; Mills, A.; Wang, L.; Han, Q. What can we learn from China's health system reform? *BMJ* **2019**, *365*, l2349, doi:10.1136/bmj.l2349.

11.	Zhang, L.; Long, J.; Jiang, W.; Shi, Y.; He, X.; Zhou, Z.; Li, Y.; Yeung, R.O.; Wang, J.; Matsushita, K., et al. Trends in Chronic Kidney Disease in China. *N Engl J Med* **2016**, *375*, 905-906, doi:10.1056/NEJMc1602469.

12.	Li, J.; Cui, Z.; Long, J.; Huang, W.; Wang, J.; Zhang, H.; Wang, H.; Zhang, L.; Ronco, P.; Zhao, M.H. Primary glomerular nephropathy among hospitalized patients in a national database in China. *Nephrol Dial Transplant* **2018**, *33*, 2173-2181, doi:10.1093/ndt/gfy022.

13.	Li, J.; Cui, Z.; Long, J.Y.; Huang, W.; Wang, J.W.; Wang, H.; Zhang, L.; Chen, M.; Zhao, M.H. The frequency of ANCA-associated vasculitis in a national database of hospitalized patients in China. *Arthritis Res Ther* **2018**, *20*, 226, doi:10.1186/s13075-018-1708-7.

14.	Chin, M.T. Basic mechanisms for adverse cardiovascular events associated with air pollution. *Heart* **2015**, *101*, 253-256, doi:10.1136/heartjnl-2014-306379.

15.	Rajagopalan, S.; Al-Kindi, S.G.; Brook, R.D. Air Pollution and Cardiovascular Disease: JACC State-of-the-Art Review. *J Am Coll Cardiol* **2018**, *72*, 2054-2070, doi:10.1016/j.jacc.2018.07.099.

16.	Li, H.; Cai, J.; Chen, R.; Zhao, Z.; Ying, Z.; Wang, L.; Chen, J.; Hao, K.; Kinney, P.L.; Chen, H., et al. Particulate Matter Exposure and Stress Hormone Levels: A Randomized, Double-Blind, Crossover Trial of Air Purification. *Circulation* **2017**, *136*, 618-627, doi:10.1161/CIRCULATIONAHA.116.026796.

17.	Miller, M.R.; Raftis, J.B.; Langrish, J.P.; McLean, S.G.; Samutrtai, P.; Connell, S.P.; Wilson, S.; Vesey, A.T.; Fokkens, P.H.B.; Boere, A.J.F., et al. Inhaled Nanoparticles Accumulate at Sites of Vascular Disease. *ACS Nano* **2017**, *11*, 4542-4552, doi:10.1021/acsnano.6b08551.

18.	Maher, B.A.; Ahmed, I.A.; Karloukovski, V.; MacLaren, D.A.; Foulds, P.G.; Allsop, D.; Mann, D.M.; Torres-Jardon, R.; Calderon-Garciduenas, L. Magnetite pollution nanoparticles in the human brain. *Proc Natl Acad Sci U S A* **2016**, *113*, 10797-10801, doi:10.1073/pnas.1605941113.

19.	North, C.M.; Rice, M.B.; Ferkol, T.; Gozal, D.; Hui, C.; Jung, S.H.; Kuribayashi, K.; McCormack, M.C.; Mishima, M.; Morimoto, Y., et al. Air Pollution in the Asia-Pacific Region: A Joint APSR/ATS Perspective. *Am J Respir Crit Care Med* **2018**, 10.1164/rccm.201804-0673PP, doi:10.1164/rccm.201804-0673PP.

20.	Schraufnagel, D.E.; Balmes, J.R.; Cowl, C.T.; De Matteis, S.; Jung, S.H.; Mortimer, K.; Perez-Padilla, R.; Rice, M.B.; Riojas-Rodriguez, H.; Sood, A., et al. Air Pollution and Noncommunicable Diseases: A Review by the Forum of International Respiratory Societies' Environmental Committee, Part 1: The Damaging Effects of Air Pollution. *Chest* **2019**, *155*, 409-416, doi:10.1016/j.chest.2018.10.042.

21.	Schraufnagel, D.E.; Balmes, J.R.; Cowl, C.T.; De Matteis, S.; Jung, S.H.; Mortimer, K.; Perez-Padilla, R.; Rice, M.B.; Riojas-Rodriguez, H.; Sood, A., et al. Air Pollution and Noncommunicable Diseases: A Review by the Forum of International Respiratory Societies' Environmental Committee, Part 2: Air Pollution and Organ Systems. *Chest* **2019**, *155*, 417-426, doi:10.1016/j.chest.2018.10.041.

22.	Zanobetti, A.; Dominici, F.; Wang, Y.; Schwartz, J.D. A national case-crossover analysis of the short-term effect of PM2.5 on hospitalizations and mortality in subjects with diabetes and neurological disorders. *Environ Health* **2014**, *13*, 38, doi:10.1186/1476-069X-13-38.

23.	Chen, Z.; Salam, M.T.; Toledo-Corral, C.; Watanabe, R.M.; Xiang, A.H.; Buchanan, T.A.; Habre, R.; Bastain, T.M.; Lurmann, F.; Wilson, J.P., et al. Ambient Air Pollutants Have Adverse Effects on Insulin and Glucose Homeostasis in Mexican Americans. *Diabetes Care* **2016**, *39*, 547-554, doi:10.2337/dc15-1795.

24.	Lee, H.; Myung, W.; Kim, D.K.; Kim, S.E.; Kim, C.T.; Kim, H. Short-term air pollution exposure aggravates Parkinson's disease in a population-based cohort. *Sci Rep* **2017**, *7*, 44741, doi:10.1038/srep44741.

25.	Kasdagli, M.I.; Katsouyanni, K.; Dimakopoulou, K.; Samoli, E. Air pollution and Parkinson's disease: A systematic review and meta-analysis up to 2018. *Int J Hyg Environ Health* **2018**, 10.1016/j.ijheh.2018.12.006, doi:10.1016/j.ijheh.2018.12.006.

26.	Power, M.C.; Kioumourtzoglou, M.A.; Hart, J.E.; Okereke, O.I.; Laden, F.; Weisskopf, M.G. The relation between past exposure to fine particulate air pollution and prevalent anxiety: observational cohort study. *BMJ* **2015**, *350*, h1111, doi:10.1136/bmj.h1111.

27.	Pun, V.C.; Manjourides, J.; Suh, H. Association of Ambient Air Pollution with Depressive and Anxiety Symptoms in Older Adults: Results from the NSHAP Study. *Environ Health Perspect* **2017**, *125*, 342-348, doi:10.1289/EHP494.

28.	Braithwaite, I.; Zhang, S.; Kirkbride, J.B.; Osborn, D.P.J.; Hayes, J.F. Air Pollution (Particulate Matter) Exposure and Associations with Depression, Anxiety, Bipolar, Psychosis and Suicide Risk: A Systematic Review and Meta-Analysis. *Environ Health Perspect* **2019**, *127*, 126002, doi:10.1289/EHP4595.

29.	Mutlu, E.A.; Engen, P.A.; Soberanes, S.; Urich, D.; Forsyth, C.B.; Nigdelioglu, R.; Chiarella, S.E.; Radigan, K.A.; Gonzalez, A.; Jakate, S., et al. Particulate matter air pollution causes oxidant-mediated increase in gut permeability in mice. *Part Fibre Toxicol* **2011**, *8*, 19, doi:10.1186/1743-8977-8-19.

30.	Kish, L.; Hotte, N.; Kaplan, G.G.; Vincent, R.; Tso, R.; Ganzle, M.; Rioux, K.P.; Thiesen, A.; Barkema, H.W.; Wine, E., et al. Environmental particulate matter induces murine intestinal inflammatory responses and alters the gut microbiome. *PLoS One* **2013**, *8*, e62220, doi:10.1371/journal.pone.0062220.

31.	Tian, L.; Qiu, H.; Sun, S.; Tsang, H.; Chan, K.P.; Leung, W.K. Association between emergency admission for peptic ulcer bleeding and air pollution: a case-crossover analysis in Hong Kong's elderly population. *Lancet Planet Health* **2017**, *1*, e74-e81, doi:10.1016/S2542-5196(17)30021-9.

32.	Wang, C.; Zhu, G.; Zhang, L.; Chen, K. Particulate matter pollution and hospital outpatient visits for endocrine, digestive, urological, and dermatological diseases in Nanjing, China. *Environ Pollut* **2020**, *261*, 114205, doi:10.1016/j.envpol.2020.114205.

33.	Yang, Y.R.; Chen, Y.M.; Chen, S.Y.; Chan, C.C. Associations between Long-Term Particulate Matter Exposure and Adult Renal Function in the Taipei Metropolis. *Environ Health Perspect* **2017**, *125*, 602-607, doi:10.1289/EHP302.

34.	Bowe, B.; Xie, Y.; Li, T.; Yan, Y.; Xian, H.; Al-Aly, Z. Particulate Matter Air Pollution and the Risk of Incident CKD and Progression to ESRD. *J Am Soc Nephrol* **2018**, *29*, 218-230, doi:10.1681/ASN.2017030253.

35.	Li, W.; Wilker, E.H.; Dorans, K.S.; Rice, M.B.; Schwartz, J.; Coull, B.A.; Koutrakis, P.; Gold, D.R.; Keaney, J.F., Jr.; Lin, H., et al. Short-Term Exposure to Air Pollution and Biomarkers of Oxidative Stress: The Framingham Heart Study. *J Am Heart Assoc* **2016**, *5*, doi:10.1161/JAHA.115.002742.

36.	Yan, Q.; Liew, Z.; Uppal, K.; Cui, X.; Ling, C.; Heck, J.E.; von Ehrenstein, O.S.; Wu, J.; Walker, D.I.; Jones, D.P., et al. Maternal serum metabolome and traffic-related air pollution exposure in pregnancy. *Environ Int* **2019**, *130*, 104872, doi:10.1016/j.envint.2019.05.066.

37.	Huang, W.; Wang, G.; Lu, S.E.; Kipen, H.; Wang, Y.; Hu, M.; Lin, W.; Rich, D.; Ohman-Strickland, P.; Diehl, S.R., et al. Inflammatory and oxidative stress responses of healthy young adults to changes in air quality during the Beijing Olympics. *Am J Respir Crit Care Med* **2012**, *186*, 1150-1159, doi:10.1164/rccm.201205-0850OC.

38.	Samet, J.M.; Dominici, F.; Curriero, F.C.; Coursac, I.; Zeger, S.L. Fine particulate air pollution and mortality in 20 U.S. cities, 1987-1994. *N Engl J Med* **2000**, *343*, 1742-1749, doi:10.1056/NEJM200012143432401.

39.	Dominici, F.; Peng, R.D.; Bell, M.L.; Pham, L.; McDermott, A.; Zeger, S.L.; Samet, J.M. Fine particulate air pollution and hospital admission for cardiovascular and respiratory diseases. *JAMA* **2006**, *295*, 1127-1134, doi:10.1001/jama.295.10.1127.

40.	Peng, R.D.; Chang, H.H.; Bell, M.L.; McDermott, A.; Zeger, S.L.; Samet, J.M.; Dominici, F. Coarse particulate matter air pollution and hospital admissions for cardiovascular and respiratory diseases among Medicare patients. *JAMA* **2008**, *299*, 2172-2179, doi:10.1001/jama.299.18.2172.

41.	Chen, R.; Yin, P.; Meng, X.; Liu, C.; Wang, L.; Xu, X.; Ross, J.A.; Tse, L.A.; Zhao, Z.; Kan, H., et al. Fine Particulate Air Pollution and Daily Mortality. A Nationwide Analysis in 272 Chinese Cities. *Am J Respir Crit Care Med* **2017**, *196*, 73-81, doi:10.1164/rccm.201609-1862OC.

42.	Liu, C.; Yin, P.; Chen, R.; Meng, X.; Wang, L.; Niu, Y.; Lin, Z.; Liu, Y.; Liu, J.; Qi, J., et al. Ambient carbon monoxide and cardiovascular mortality: a nationwide time-series analysis in 272 cities in China. *Lancet Planet Health* **2018**, *2*, e12-e18, doi:10.1016/S2542-5196(17)30181-X.

43.	Wang, L.; Liu, C.; Meng, X.; Niu, Y.; Lin, Z.; Liu, Y.; Liu, J.; Qi, J.; You, J.; Tse, L.A., et al. Associations between short-term exposure to ambient sulfur dioxide and increased cause-specific mortality in 272 Chinese cities. *Environ Int* **2018**, *117*, 33-39, doi:10.1016/j.envint.2018.04.019.

44.	Kan, H.; London, S.J.; Chen, G.; Zhang, Y.; Song, G.; Zhao, N.; Jiang, L.; Chen, B. Differentiating the effects of fine and coarse particles on daily mortality in Shanghai, China. *Environ Int* **2007**, *33*, 376-384, doi:10.1016/j.envint.2006.12.001.

45.	Xie, W.; Li, G.; Zhao, D.; Xie, X.; Wei, Z.; Wang, W.; Wang, M.; Li, G.; Liu, W.; Sun, J., et al. Relationship between fine particulate air pollution and ischaemic heart disease morbidity and mortality. *Heart* **2015**, *101*, 257-263, doi:10.1136/heartjnl-2014-306165.

46.	Tian, Y.; Liu, H.; Wu, Y.; Si, Y.; Song, J.; Cao, Y.; Li, M.; Wu, Y.; Wang, X.; Chen, L., et al. Association between ambient fine particulate pollution and hospital admissions for cause specific cardiovascular disease: time series study in 184 major Chinese cities. *BMJ* **2019**, *367*, l6572, doi:10.1136/bmj.l6572.

47.	Gu, J.; Shi, Y.; Chen, N.; Wang, H.; Chen, T. Ambient fine particulate matter and hospital admissions for ischemic and hemorrhagic strokes and transient ischemic attack in 248 Chinese cities. *Sci Total Environ* **2020**, *715*, 136896, doi:10.1016/j.scitotenv.2020.136896.

48.	Chen, R.; Yin, P.; Meng, X.; Wang, L.; Liu, C.; Niu, Y.; Lin, Z.; Liu, Y.; Liu, J.; Qi, J., et al. Associations Between Ambient Nitrogen Dioxide and Daily Cause-specific Mortality: Evidence from 272 Chinese Cities. *Epidemiology* **2018**, *29*, 482-489, doi:10.1097/EDE.0000000000000829.

49.	Chen, R.; Yin, P.; Meng, X.; Wang, L.; Liu, C.; Niu, Y.; Liu, Y.; Liu, J.; Qi, J.; You, J., et al. Associations between Coarse Particulate Matter Air Pollution and Cause-Specific Mortality: A Nationwide Analysis in 272 Chinese Cities. *Environ Health Perspect* **2019**, *127*, 17008, doi:10.1289/EHP2711.

50.	Tian, Y.; Liu, H.; Liang, T.; Xiang, X.; Li, M.; Juan, J.; Song, J.; Cao, Y.; Wang, X.; Chen, L., et al. Ambient air pollution and daily hospital admissions: A nationwide study in 218 Chinese cities. *Environ Pollut* **2018**, *242*, 1042-1049, doi:10.1016/j.envpol.2018.07.116.

51.	Hoffmann, B.; Luttmann-Gibson, H.; Cohen, A.; Zanobetti, A.; de Souza, C.; Foley, C.; Suh, H.H.; Coull, B.A.; Schwartz, J.; Mittleman, M., et al. Opposing effects of particle pollution, ozone, and ambient temperature on arterial blood pressure. *Environ Health Perspect* **2012**, *120*, 241-246, doi:10.1289/ehp.1103647.

52.	Samoli, E.; Analitis, A.; Touloumi, G.; Schwartz, J.; Anderson, H.R.; Sunyer, J.; Bisanti, L.; Zmirou, D.; Vonk, J.M.; Pekkanen, J., et al. Estimating the exposure-response relationships between particulate matter and mortality within the APHEA multicity project. *Environ Health Perspect* **2005**, *113*, 88-95, doi:10.1289/ehp.7387.

53.	Bell, M.L.; Peng, R.D.; Dominici, F. The exposure-response curve for ozone and risk of mortality and the adequacy of current ozone regulations. *Environ Health Perspect* **2006**, *114*, 532-536, doi:10.1289/ehp.8816.

54.	Cohen, A.J.; Brauer, M.; Burnett, R.; Anderson, H.R.; Frostad, J.; Estep, K.; Balakrishnan, K.; Brunekreef, B.; Dandona, L.; Dandona, R., et al. Estimates and 25-year trends of the global burden of disease attributable to ambient air pollution: an analysis of data from the Global Burden of Diseases Study 2015. *Lancet* **2017**, *389*, 1907-1918, doi:10.1016/S0140-6736(17)30505-6.

55.	Lanzinger, S.; Schneider, A.; Breitner, S.; Stafoggia, M.; Erzen, I.; Dostal, M.; Pastorkova, A.; Bastian, S.; Cyrys, J.; Zscheppang, A., et al. Ultrafine and Fine Particles and Hospital Admissions in Central Europe. Results from the UFIREG Study. *Am J Respir Crit Care Med* **2016**, *194*, 1233-1241, doi:10.1164/rccm.201510-2042OC.

56.	Di, Q.; Dai, L.; Wang, Y.; Zanobetti, A.; Choirat, C.; Schwartz, J.D.; Dominici, F. Association of Short-term Exposure to Air Pollution With Mortality in Older Adults. *JAMA* **2017**, *318*, 2446-2456, doi:10.1001/jama.2017.17923.

57.	Golzari, S.E.; Soleimanpour, H.; Rahmani, F.; Zamani Mehr, N.; Safari, S.; Heshmat, Y.; Ebrahimi Bakhtavar, H. Therapeutic approaches for renal colic in the emergency department: a review article. *Anesth Pain Med* **2014**, *4*, e16222, doi:10.5812/aapm.16222.

58.	Lipsitch, M.; Tchetgen Tchetgen, E.; Cohen, T. Negative controls: a tool for detecting confounding and bias in observational studies. *Epidemiology* **2010**, *21*, 383-388, doi:10.1097/EDE.0b013e3181d61eeb.
