# Supplementary material for: Ambient air pollution and cause-specific risk of hospital admission in China: A nationwide time-series study
Source: PLoS Med. 2020 Aug 6;17(8):e1003188. doi: 10.1371/journal.pmed.1003188 (PMC7410211; doi:10.1371/journal.pmed.1003188)
Supplement: Attachment — Submitted filename: Response letter.docx [file pmed.1003188.s037.docx]

**Manuscript ID: PMEDICINE-D-19-04229R2**

**Title:** Ambient air pollution and cause-specific risk of hospital admissions in China: A nationwide time-series study

Jiangshao Gu; Ying Shi; Yifang Zhu; Ning Chen; Haibo Wang; Zongjiu Zhang; Ting Chen

**Response letter**

Dear Editor,

We would like to thank the Reviewers and Editors for the insightful review on our manuscript entitled “Ambient air pollution and cause-specific risk of hospital admissions in China: A nationwide time-series study” (PMEDICINE-D-19-04229R2). We have considered the comments and suggestions carefully, and made revisions to the manuscript. Detailed point-to-point replies to the comments are provided in next pages.

Thank you for the consideration of our revision.

Best regards,

Jiangshao Gu

Ting Chen

**Requests from Editors:**

**[The Editors, Comment #1]**

At line 526 I think you mean to say risk of admission (rather "risk of disease")? Please amend.

**[Response to The Editors, Comment #1]**

We appreciate the helpful suggestion. We have revised the sentence as “Our analysis showed that air pollution exposure was associated with increased risk of hospitalization for diseases of multiple organ systems, including certain diseases of the digestive, musculoskeletal, and genitourinary systems, many of which are important but still not fully recognized.”

**[The Editors, Comment #2]**

Can you please check the given link for data as this doesn’t seem to retrieve the suggested link, for me at least ((https://spms.hqms.org.cn/).

**[Response to The Editors, Comment #2]**

We appreciate the helpful suggestion. The link for the Scientific Project Management System (https://spms.hqms.org.cn/) is available now. We have revised the Data Availability Statement as follows:

[Data Availability Statement] “Air pollution data can be accessed from the National Air Pollution Monitoring System (http://106.37.208.233:20035/). Meteorological data can be accessed from the China Meteorological Data Sharing Service System (http://data.cma.cn/). Hospital admission data were obtained from the Hospital Quality Monitoring System (HQMS) (https://www.hqms.org.cn/) and cannot be made publicly available due to ethical and legal restrictions. These routinely collected healthcare data, though anonymized and de-identified, contain potentially identifying or sensitive patient information. Data that are not directly identifying, including the date of admission, clinical diagnosis, and demographic information, can become identifying in combination. According to the Personal Information Protection Law in the People’s Republic of China, these data cannot be shared publicly. The data are available upon request for researchers who meet the criteria for access to confidential data. Data requesters are required to submit a research proposal to the scientific committee of the HQMS via the Scientific Project Management System (https://spms.hqms.org.cn/), under the regulation of Bureau of Medical Administration, National Health Commission of the People’s Republic of China. The research proposal should address: (1) the background and rationale of the proposed research project; (2) the scope of the relevant data (eg, the list of relevant data items, time range, hospital scope, and the list of relevant ICD-10 codes); (3) the significance of the expected results in terms of informing public health policy makers. The scientific committee of the HQMS will review the research proposal to ensure the appropriateness of its intended use, and the data will be available if the proposal is approved. For more information and for technical assistance, please contact the HQMS staff (service@hqms.org.cn).”

**[The Editors, Comment #3]**

Please use the "Vancouver" style for reference formatting, and see our website for other reference guidelines https://journals.plos.org/plosmedicine/s/submission-guidelines#loc-references (specifically, no bold or ital font and only the first 6 authors names followed by et al)

**[Response to The Editors, Comment #3]**

We appreciate the helpful suggestion. We have re-formatted the references accordingly.

**Production requirements:**

**[FIGURES and GRAPHICS]**

1. Please remove embedded figures from your manuscript file and ensure the most up to date versions of your figures are uploaded to our submission system as separate TIF or EPS files. Please do not remove the figure legends and captions.

2. Please ensure that all main figure files are cited in ascending numerical order in the main text of the article.

3. Please ensure that all main figures are referenced in the manuscript as Fig 1, Fig 2, etc. (including capitalization), rather than Figure 1, Figure 2, etc. Please note, however, that the file names themselves must not include the space, i.e. Fig1.tif, Fig2.tif, etc.

4. Please ensure that all main figure legends are placed after the paragraph containing their first in-text citation.

5. Please note that your figures do not currently comply with our publication requirements. For further instructions to assist with your Figure revisions, please refer to our Figure Guidelines at: http://journals.plos.org/plosbiology/s/figures.

If you need assistance, please contact us directly at graphics@jjeditorial.com so we can further help with this matter. Please send any relevant source files and tell us what application(s) and what operating system you are using.

-Figure 1 is low resolution and will appear blurry when published. This error cannot be fixed by simply increasing the resolution of your figures with image editing software. Instead, please carefully review the PLOS Figure Guidelines and provide figure files that were originally created at a minimum of 300 pixels/inch (ppi) and that are sized between 6.68cm - 19.05cm wide, and no more than 22.225cm tall (excluding any white space around your artwork). Additionally, please ensure your figures are in RGB colour mode.

-Figure 1 exceeds the dimension limits for figures. Figures must be less than 19.05 cm in width, and less than 22.225cm in height (excluding white space around your artwork). Please make the necessary adjustments and re-upload your figure(s).

-In Photoshop: Image→Image Size. Make sure the Resample Image checkbox is NOT checked. If the Width is over 19.05cm, type 19.05 in the Width box (19.05cm is our maximum allowable width for figures).

-In GIMP: Image→Scale Image. Set the units of measurement, in the pull down menu next to Height, to millimeters. If the Width is over 190.5mm, type 190.5 in the Width box (19.05cm is our maximum allowable width for figures) and hit Tab. The new Height of the figure will appear, scaled proportionately to the change in Width.

-Figure 1 has been submitted in a layered TIF file format which we don’t accept for figures. To ensure that your figures publish properly, please flatten the layers in each figure file (Layer→Flatten Image in Photoshop/GIMP) and re-upload. Make sure to save your TIF files with LZW Compression turned on.

-Figures 2 and 3 are good for publication.

**[Response]**

Thanks. We have updated FIGURES and GRAPHICS accordingly. Figure 1 has been fixed.

**[TABLES]**

1. Tables 3 and 4 use returns to control text wrapping. This is indicated by a [¶ or ↵] symbol when "Show Paragraph Marks" is turned on in Word. Please either split those cells into separate cells to achieve alignment or remove the returns.

**[Response]**

Thanks. We have updated TABLES accordingly. The returns have been removed.

**[METADATA]**

1. Please ensure that the funders and grant numbers match between the Financial Disclosure field and the Funding Information tab in your submission form. Note that the funders must be provided in the same order in both places as well.

2. In accordance with PLOS' data policy, please ensure that your Data Availability Statement in the submission form clearly identifies how readers can access your data. Data cannot be available on request, or only accessible by contacting one of the authors. Note that your Data Availability Statement will be typeset as it is written, so please ensure it is in complete sentences and appears as you would like it to in the published version.

For details on our data policy, please see http://journals.plos.org/plosmedicine/s/data-availability

3. In the online submission form, you indicated that "Researchers wishing to access the data of this study are required to submit a research proposal to the scientific committee of the Hospital Quality Monitoring System (HQMS) via the Scientific Project Management System (https://spms.hqms.org.cn/), under the regulation of Bureau of Medical Administration, National Health Commission of the People’s Republic of China. The research proposal should address: (1) the background and rationale of the proposed research project; (2) the scope of the relevant data (eg, the list of relevant data items, time range, hospital scope, and the list of relevant ICD-10 codes); (3) the significance of the expected results in terms of informing public health policy makers. The scientific committee of the HQMS will review the research proposal and the data will be available if the proposal is approved. For more information and for technical assistance, please contact the HQMS staff (service@hqms.org.cn)." All PLOS journals now require all data underlying the findings described in their manuscript to be freely available to other researchers, either 1. In a public repository, 2. Within the manuscript itself, or 3. Uploaded as supplementary information.

This policy applies to all data except where public deposition would breach compliance with the protocol approved by your research ethics board. If your data cannot be made publicly available for ethical or legal reasons (e.g., public availability would compromise patient privacy), please explain this by return email and staff will assist you with completing your Data Availability Statement.

At this time, we ask that you please explain, with as much detail as possible, why you are unable to make your data available in one of the three places mentioned above so we can further assess whether or not this complies with PLOS’ Data Policy and determine how we can assist you in this matter.

**[Response]**

Thanks. We have updated METADATA accordingly. The revised Data Availability Statement has been approved by Publishing Editor of the journal.

**[ARTICLE FILE]**

1. Please indicate the corresponding author in the byline by placing an asterisk (*) after their affiliation number.

2. Please include the corresponding author's email address on the title page of your manuscript, indicated by an asterisk (*). Only an asterisk and the email address itself are required.

**[Response]**

Thanks. We have updated ARTICLE FILE accordingly.

**[REFERENCES]**

1. Please ensure your references are in the style of PLOS Medicine (ICMJE/Vancouver style). The reference style guide can be found here: http://journals.plos.org/plosmedicine/s/submission-guidelines#loc-references. You can also find a downloadable EndNote file in these guidelines to assist you. Please do a manual check as well, as the EndNote file does not catch everything. Please note that the references will be typeset as they appear so please ensure the format is consistent.

2. Please put all reference citations in square brackets separated by either commas or dashes with no spaces, e.g. [1,2,3] or [1-3].

3. Please make sure all references are cited in ascending numerical order in the text.

**[Response]**

Thanks. We have updated REFERENCES accordingly.

**[SUPPORTING INFORMATION]**

1. Please upload your supporting information as individual files, and use the following naming format: S1 Text; S1 Fig; S1 Table, S1 Data, etc. Each file type should start as S1 and increase numerically: S1 Text, S2 Text, etc. Note that all relevant in-text citations and captions will need to be updated accordingly.

2. Please ensure that all Supporting Information files use the following format verbatim throughout the manuscript (including legends and all in-text citations): S1 Fig, S1 Table, S1 Text, S1 Data, etc. Each file type should start at 1 and increase numerically. Please note, however, that the file names themselves must use an underscore rather than a space, i.e. S1_Fig.tif, S1_Table.xlsx, S1_Text.doc, S1_Data.xlsx, S1_PRISMA_Checklist.docx, etc.

3. Please ensure that each supporting information file has a legend at the end of the manuscript file, after the Reference list.

4. Please note that your submission cannot contain any references to generic "supporting information" files and instead must refer to specific files using our naming convention (S1 Fig, S1 Table, S1 Text, S1 Data, S1 PRISMA Checklist, etc.)

5. Please include the location from which you obtained the base map used in S1 Fig in the legends for this figure. Please note that, because all PLOS articles are published under a CC BY license (creativecommons.org/licenses/by/4.0/), we cannot publish proprietary maps such as Google Maps, Mapquest or other copyrighted maps. If your map was obtained from a copyrighted source please amend the figure so that the base map used is from an openly available source.

The following websites are good examples of where you can source open access or public domain maps:

* World of Maps - Many, but not all, of the maps are in the public domain so please check the usage rights before downloading. (http://www.worldofmaps.net)

* U.S. Geological Survey (USGS) - All maps are in the public domain. (http://www.usgs.gov)

* PlaniGlobe - All maps are published under a Creative Commons license so please cite “PlaniGlobe, http://www.planiglobe.com, CC BY 2.0” in the image credit after the caption. (http://www.planiglobe.com/?lang=enl)

* Natural Earth - All maps are public domain. (http://www.naturalearthdata.com/about/terms-of-use/)

**[Response]**

Thanks. We have updated SUPPORTING INFORMATION accordingly. The base map for S1 Fig was created using the open source R package *maps*, which is an openly available source for publication under a CC BY license, commonly used in previous *PLOS Medicine* articles.
